# Corticosteroids for Treating Sepsis in Adult Patients: A Systematic Review and Meta-Analysis

**DOI:** 10.3389/fimmu.2021.709155

**Published:** 2021-08-16

**Authors:** Huoyan Liang, Heng Song, Ruiqing Zhai, Gaofei Song, Hongyi Li, Xianfei Ding, Quancheng Kan, Tongwen Sun

**Affiliations:** ^1^ General ICU, The First Affiliated Hospital of Zhengzhou University, Henan Key Laboratory of Critical Care Medicine, Zhengzhou Key Laboratory of Sepsis, Henan Engineering Research Center for Critical Care Medicine, Zhengzhou, China; ^2^ Academy of Medical Sciences, Zhengzhou University, Zhengzhou, China; ^3^ College of Bioinformatics Science and Technology, Harbin Medical University, Harbin, China; ^4^ Department of Pharmacy, The First Affiliated Hospital of Zhengzhou University, Zhengzhou, China

**Keywords:** corticosteroids, sepsis, mortality, meta-analysis, systematic review

## Abstract

**Objective:**

Corticosteroids are a common option used in sepsis treatment. However, the efficacy and potential risk of corticosteroids in septic patients have not been well assessed. This review was performed to assess the efficacy and safety of corticosteroids in patients with sepsis.

**Methods:**

PubMed, Embase, and Cochrane library databases were searched from inception to March 2021. Randomized controlled trials (RCTs) that evaluated the effect of corticosteroids on patients with sepsis were included. The quality of outcomes in the included articles was evaluated using the Grading of Recommendations Assessment, Development, and Evaluation methodology. The data were pooled by using risk ratio (RR) and mean difference (MD). The random-effects model was used to evaluate the pooled MD or RR and 95% confidence intervals (CIs).

**Results:**

Fifty RCTs that included 12,304 patients with sepsis were identified. Corticosteroids were not associated with the mortality in 28-day (RR, 0.94; 95% CI, 0.87–1.02; evidence rank, moderate) and long-term mortality (>60 days) (RR, 0.96; 95% CI, 0.88–1.05) in patients with sepsis (evidence rank, low). However, corticosteroids may exert a significant effect on the mortality in the intensive care unit (ICU) (RR, 0.9; 95% CI, 0.83–0.97), in-hospital (RR, 0.9; 95% CI, 0.82–0.99; evidence rank, moderate) in patients with sepsis or septic shock (evidence rank, low). Furthermore, corticosteroids probably achieved a tiny reduction in the length of hospital stay and ICU. Corticosteroids were associated with a higher risk of hypernatremia and hyperglycemia; furthermore, they appear to have no significant effect on superinfection and gastroduodenal bleeding.

**Conclusions:**

Corticosteroids had no significant effect on the 28-day and long-term mortality; however, they decreased the ICU and hospital mortality. The findings suggest that the clinical corticosteroids may be an effective therapy for patients with sepsis during the short time.

**Systematic Review Registration:**

https://inplasy.com/wp-content/uploads/2021/05/INPLASY-Protocol-1074-4.pdf

## Introduction

Sepsis is a life-threatening organ dysfunction, which is caused by a dysregulated host response to infection (1, 2) that culminates in systematic hypoperfusion and considerable organ dysfunction. The main therapies to treat sepsis in the early phase are antibiotic administration and perfusion restoration (3). Early and aggressive treatment is associated with a mortality rate of 30%–50% in critically ill patients admitted to the intensive care unit (ICU) and induces more than 5 million deaths each year across the world (3, 4). Therefore, further investigation for the treatment of sepsis is crucial.

The pathology of sepsis is marked by a dysregulated host response to infection; therefore, immunomodulatory therapies have been used in sepsis treatment that may be effective (5). Doctors have started using corticosteroids as an adjuvant therapy for sepsis since the middle of the twentieth century (3). Corticosteroids were used to treat sepsis, especially the septic shock therapy; numerous randomized clinical trials (RCTs) were performed to evaluate the safety and efficacy of corticosteroids. However, the results of these RCTs varied. Thus, many systematic reviews have been performed to assess the safety and efficacy of corticosteroids in patients with sepsis. However, the results of the most recent reviews remain controversial (6, 7). Subsequently, several studies have further assessed whether the combination of corticosteroids, vitamin C, and thiamine as compared with corticosteroids or placebo improved the survival duration, increased the vasopressor-free time over 7 days, and reduced organ injury (8, 9). These results suggest that the use of corticosteroids in combination with other drugs did not affect the safety and efficacy of corticosteroids in patients with sepsis. Hence, resolution of this controversy regarding the latest reviews that have assessed the efficacy of corticosteroids in patients with sepsis is currently the primary problem in sepsis treatment. Therefore, this systematic review and meta-analysis were performed based on the latest reviews to reintegrate the relevant data to evaluate the effects and safety of corticosteroids in patients with sepsis.

## Methods

The protocol of this systematic review and meta-analysis was registered on INPLASY (ID: INPLASY2020110122). The methodology of this study was according to items of the Cochrane Collaboration, and each outcome was assessed using the Grading of Recommendations Assessment, Development, and Evaluation (GRADE) guidelines (10).

### Study Searches

**Figure 1:**
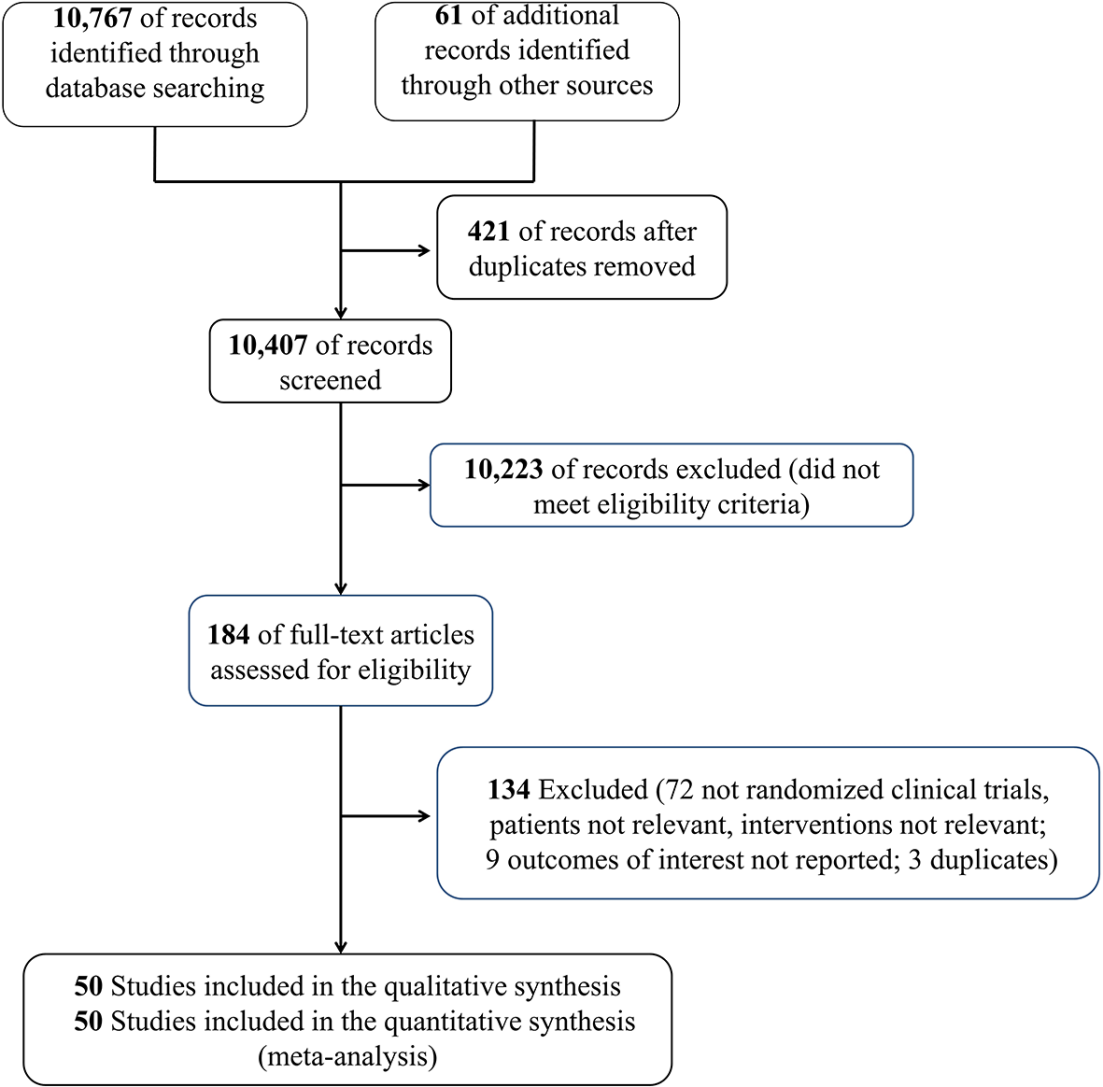
Flowchart of the search strategy in this meta-analysis.

This meta-analysis was performed based on the Preferred Reporting Items for Systematic Reviews and Meta-analyses (PRISMA) criteria. Moreover, the PRISMA 2020 checklist is shown in **Supplemental Table 1**. PubMed, Embase, and Cochrane library databases were searched for relevant data from inception to March 2021, update to 5 July 2021, to identify RCTs that have evaluated the effect of corticosteroids on patients with sepsis. The MeSH/Emtree and title/abstract keyword combination were used to identify the eligible articles; the keyword search terms used for the English literature included the words corticosteroids and sepsis (detailed search strategy in **Supplemental Table 2**). It is noteworthy that we also conducted a manual search for the references of the relevant articles (study search flowchart in **Figure 1**).

### Study Selection

Before the potential articles were searched and screened, the eligibility criteria and exclusion criteria were identified. Articles may be eligible according to the inclusion criteria in this study if they meet all of the following conditions: (1) adult patients diagnosed with sepsis, severe sepsis, or septic shock, as per the inclusion criteria during the study (11–13) [studies reporting adult patients with acute respiratory distress syndrome (ARDS) and sepsis were included]; (2) the study compared the use of corticosteroids (including hydrocortisone, methylprednisolone, betamethasone, fludrocortisone, and dexamethasone) with no use of corticosteroids; (3) the study measured and reported the outcomes in terms of 28-day and long-term mortality (>60 days), ICU mortality, in-hospital mortality, length of stay in hospital and ICU, vasopressor-free days, ventilation-free time, shock reversal at days 7 and 28, time for resolution of shock, Sequential Organ Failure Assessment (SOFA) scores at day 7, hypernatremia, hyperglycemia, superinfection, and gastroduodenal bleeding; (4) the study was an RCT or abstract and was published in English. Furthermore, the study design including case reports, case series, and observational studies or the previous unpublished studies that required the author to be contacted were excluded. All the available articles were searched by two searchers, respectively, and when disagreements occurred during the process, the third investigator should resolve these disagreements. Reviewers performed reviews in pairs to screen all relevant citations and references as per the search strategy, and the screening process included the following two stages: initial evaluation of titles and abstracts and skimming of the full text to identify the eligible studies.

### Data Extraction

Researchers conducted data extraction, respectively, and in duplicate based on the eligibility and exclusion criteria. In case of disagreements, the third reviewer resolved the issue. Relevant data, including the study title, first author, study type, study period, the therapy in treatment and control groups, reported outcomes, sepsis definition, and so forth were collected. The data only for the studies that we searched including the previous review (6) were abstracted. The risk of bias for this meta-analysis was assessed by two investigators independently for every abstracted data of each article based on the Cochrane Collaboration (14) and domains including allocation concealment, blinding of participants and staff, blinding of outcome assessors, incomplete outcome data, selective outcome reporting, and other biases. Additionally, the GRADE framework was used to evaluate the overall evidence rank for every outcome (15). The studies with more than six, four, to six and fewer than six items were considered high, fair, and poor quality, respectively. Importantly, the GRADE was used to assess the evidence rank of mortality and adverse events. According to the risk of bias, inconsistency, indirectness, imprecision, and publication bias, the studies were evaluated as low, moderate, or high quality.

### Statistical Analyses

Mantel–Haenszel (M-H) or DerSimonian Laird (DL) methods with random-effects meta-analyses were conducted for the eligible RCTs. All the relevant data were assessed using the Review Manager (RevMan), version 5.3 (Cochrane Collaboration), STATA 16.0 (StataCorp, College Station, TX, USA). Risk ratio (RR) and mean difference (MD) were used to present the dichotomous and continuous outcomes, with 95% CI. Moreover, a Funnel plot was used to examine the potential for some small effects if the outcome included more than 10 trials, and the possibility of publication bias was assessed using the Funnel plot and Egger regression test (16). The chi-square test, *I* ^2^, and visual inspection of the forest plots were used to evaluate heterogeneity among the eligible studies; when *I* ^2^ was >50%, the heterogeneity was considered substantial. In addition, we performed the subgroup analyses based on the following variables: sepsis subtype [sepsis, septic shock, sepsis and ARDS, sepsis and community-acquired pneumonia (CAP), and severe COVID-19], type of corticosteroids (hydrocortisone or hydrocortisone plus fludrocortisone or methylprednisolone or prednisone or betamethasone or dexamethasone), and type of ICU [surgical, medical (internal) or surgical/medical ICU], searching the source of heterogeneity. Additionally, as the unit dose of the corticosteroids varied, relevant included studies about the use of corticosteroids were collected based on catecholamine use for qualitative analysis.

## Results

### Characteristics of Eligible Studies

We initially identified 10,828 records, and 10,407 citations remained after the duplicate trials were removed; 184 RCTs were eligible after preliminary screening by title and abstract. Finally, 50 RCTs (17–66) that included 12,304 patients with sepsis were included in this meta-analysis (**Figure 1**). The characteristics of the included RCTs are listed in **Table 1**. Twenty-five RCTs (18, 20–23, 25–29, 34, 35, 37, 39, 40, 43, 45, 51, 54, 56–60, 64) on 8,400 patients with septic shock, 8 RCTs (36, 38, 40, 42, 47, 52, 65, 66) on 936 patients with sepsis, 4 RCTs (17, 44, 46, 62) on 390 patients with sepsis and ARDS, 8 RCTs (24, 30, 33, 48, 50, 53, 55, 63) on 1,699 patients with sepsis and community-acquired pneumonia, and 4 RCTs (19, 31, 32, 61) on 748 patients with severe COVID-19 were included. Additionally, 27 RCTs (19, 23, 25, 27, 28, 30–32, 34, 35, 37–41, 43, 45, 47, 49–53, 56, 58, 59, 64) (6,981 patients) of which were treated with hydrocortisone, 4 RCTs (18, 20–22) (2,082 patients) with hydrocortisone plus fludrocortisone, 10 RCTs (17, 26, 32, 33, 36, 44, 46, 54, 57, 63) (1,245 patients) with methylprednisolone, 4 RCTs (33, 55, 65, 66) (364 patients) with prednisolone, 3 RCTs (29, 48, 61) (408 patients) with dexamethasone, and only 1 RCT (42) with betamethasone (85 patients). Furthermore, 6 RCTs (39, 51, 60, 62, 65, 66) recruited eligible patients into the medical (internal) ICU and 3 studies (23, 29, 54) into the surgical ICU, and the remaining 40 studies were reported in the medical/surgical ICU or ICU. Moreover, 24 studies (17, 20–23, 25, 27–29, 31, 34, 35, 39, 41, 43, 45, 47, 48, 51, 56–58, 60, 61) showed corticosteroids use based on catecholamine in patients with septic shock. Specifically, 16 RCTs (20–23, 31, 34, 35, 39, 41, 43, 45, 56, 58, 60, 61) reported corticosteroids dose to be not more than 200 mg/day or 50 mg every 6 h; 8 RCTs (17, 25, 27–29, 47, 48, 51) showed a dose more than 200 mg/day (most of which were 200–300 mg/day). In addition, 21 studies (26, 27, 32, 33, 35–37, 39, 42, 44, 45, 47, 48, 51, 54, 55, 57, 62, 63, 65, 66) showed the time of corticosteroids administration in patients with sepsis, 16 RCTs (26, 27, 32, 33, 35–37, 39, 42, 44, 45, 51, 54, 55, 57, 65) of which reported the time of corticosteroids administration within 2 h for prognosis or randomization or as soon as possible and 5 RCTs (47, 48, 62, 63, 66) at 12 h or more after admission.

**Table 1:** Characteristics of the included studies in the in adult patients with sepsis.

Study	Study Type	S/M Center	Study Period	Total Patients/Patients in CS No.	Mean Age, Years	Female/Male of Patient No.	Type of Patient Population	Sepsis or Septic Shock Definition		The time of CS Administration	Experimental Intervention	Reported Outcomes		
Annane et al. (21)	RCT	M	NA	1241/614	CS: 66 PC: 66	415/826	Septic shock	Sepsis-3		NA	50 mg/6 h hydrocortisone intravenously + fludrocortisone 50 µg for 7 days	28, 90, and 180 days, ICU and hospital discharge, etc.		
Venkatesh et al. (64)	RCT	M	03/2013 −04/2017	3,658/1,832	CS: 62.3 PC: 62.7	1,399/2,259	Septic shock	Sepsis-3		NA	200 mg/day hydrocortisone intravenous infusion for 7 days	90- and 28-day mortality; ICU/hospital stay time, etc.		
Annane et al. (22)	RCT	M	10/1995 −02/1999	300/151	CS: 62 PC: 60	200/100	Septic shock	Sepsis-2		NA	50 mg/6 h hydrocortisone bolus and 50 μg fludrocortisone orally/24 h for 7 days	28-day mortality		
Lv et al. (45)	RCT	S	09/2015–09/2016	118/58	CS: 68.8 PC: 64.8	70/68	Septic shock	NA		With vasoactive drugs initiating	200 mg/day hydrocortisone for 6 days	28-day mortality; reversal of shock; hospital mortality; ICU/hospital stay		
Klastersky et al. (42)	RCT	S	NA	85/46	NA	47/38	Severe sepsis	NA		With antibacterial agents	Betamethasone 0.5 mg/kg every 12 h for 3 days	30-day mortality		
Bone et al. (26)	RCT	M	11/1982–12/1985	382/191	CS:53.0 PC: 53.6	147/235	Septic shock	NA	2 h from entry		Methylprednisolone bolus (30 mg/kg) repeated every 6 h for 24 h	Shock incidence; shock reversal; overall mortality; 14-day mortality		
Schumer et al. (54)	RCT	S	1967–1975	172/86	50	5/167	Septic shock	NA	At the time of diagnosis		Methylprednisolone (30 mg/kg) dose was repeated once in both groups after 4 h	Mortality; shock associated mortality; organ injury associated mortality		
Sprung et al. (57)	RCT	M	08/1979–02/1982	59/43	CS: 58 PC: 55	13/46	Septic shock	NA	After the onset of shock		Methylprednisolone (30 mg/kg);	Shock reversal; hospital mortality; blood cultures; adverse events		
Yildiz et al. (65)	RCT	S	05/1997–04/1999	40/20	CS: 57.8 PC: 56.5	16/24	Sepsis	Sepsis-1	Within 2 h after randomization		Prednisolone intravenous blouses 2 times/day at 6:00 a.m. (5 mg) and at 6:00 p.m. (2.5 mg) for 10 days	28-day mortality; sepsis-related organ dysfunction		
Vasscsg et al. (36)	RCT	M	10/1983–04/1986	223/112	CS: 60.9 PC: 60.6	NA	Sepsis	NA	Within 2 h of diagnosis		Methylprednisolone bolus (30 mg/kg) repeated every 6 h for 24 h	14-day mortality; adverse occurrences		
Luce et al. (44)	RCT	S	09/1983–08/1986	75/38	NA	NA	Sepsis and ARDS	NA	After patients inclusion		Methylprednisolone (30 mg/kg) every 6 h, 4 times	ARDS incidence; all-cause mortality; adverse events		
Bollaert et al. (25)	RCT	M	NA	41/22	CS: 66 PC: 56	14/27	Septic shock	Sepsis-1	NA		Hydrocortisone bolus (100 mg) every 8 h for 5 days, then tapered over 6 days	7 days reversal of shock; 28 days reversal of shock; 28-day mortality; adverse events		
Tilouch et al. (60)	RCT	S	04/2013–06/2016	70/33	CS (continuous infusion): 69 CS (bolus): 70	43/27	Septic shock	NA	NA		Hydrocortisone 200 mg/days by continuous infusion for 7 days; Hydrocortisone 50 mg intravenously every 6 h for 7 days	Shock reversal at day 7; 28-day mortality; vasopressor-free days; ICU and hospital length of stay; occurrence of superinfection		
Huang et al. (38)	RCT	S	12/2010–12/2012	60/20	CS: 53.9 PC: 55.7	25/35	Sepsis	Sepsis-2	NA		Hydrocortisone (300 mg) daily as a continuous infusion for 7 days	28-day mortality; 3 days shock reversal		
Briegel et al. (27)	RCT	S	NA	40/20	CS: 47 PC: 51	19/21	Septic shock	Sepsis-1	Within 30 min		Hydrocortisone bolus (100 mg), followed by a continuous infusion of 0.18 mg/kg per hour until shock reversal, then tapered off	Shock reversal; hemodynamic variables; MOSD	
Chawla et al. (28)	RCT	S	NA	44/23	NA	NA	Septic shock	NA	NA		Hydrocortisone (100 mg)/8 h for 3 days	Shock reversal	
Loisa et al. (43)	RCT	S	07/2005–04/2006	48/NA	NA	NA	Septic shock	Sepsis-2	NA		Hydrocortisone intravenous bolus of 50 mg/6 h for 5 days	Reversal of shock	
Confalonieri et al. (30)	RCT	M	07/2000–03/2003	46/23	CS: 60.4 PC: 66.6	14/32	Sepsis and CAP	NA	NA		Hydrocortisone bolus (200 mg), followed by a continuous infusion of 10 mg/h for 7 days, then tapered off over 4 days	MODS score by Study Day 8 and development of delayed septic shock; duration of mechanical ventilation; length of ICU/RIU hospital stay; survival to hospital discharge and to 60 days	
Sprung et al. (56)	RCT	M	03/2002–11/2005	499/251	CS: 63 PC: 63	167/332	Septic shock	Sepsis-1	NA		Hydrocortisone (50 mg)/6 h for 5 days, then 50 mg/12 h for 3 days, then 50 mg/day for 3 days	28-day mortality	
Keh et al. (40)	RCT	S	03/1997–09/2000	40/20	52	26/14	Septic shock	Sepsis-1	NA		Hydrocortisone bolus (100 mg) followed by a continuous infusion of 10 mg/h for 3 days	Plasma cortisol	
Arabi et al. (23)	RCT	S	04/2004–10/2007	75/39	CS: 44 PC: 44	33/42	Cirrhosis and septic shock	Sepsis-2	NA		Hydrocortisone bolus (50 mg)/6 h until shock resolution	28-day mortality;shock reversal; ventilation-free days; length of stay in ICU; length of stay in hospital	
Fernandez-Serrano et al. (33)	RCT	S	NA	56/28	CS: 61 PC: 66	NA	Severe CAP	NA	30 min before starting the antibiotic treatment		Methylprednisolone as an intravenous bolus of 200 mg administered 30 min followed by 29 mg/6 h for 3 days, then 20 mg/12 h for 3 days, and finally 20 mg/days for another 3 days	Need for mechanical ventilation; time to resolution of morbidity score ICU length of stay; hospital length of stay	
Rinaldi et al. (52)	RCT	S	NA	40/20	CS: 68 PC: 66	NA	Severe sepsis	Sepsis-1	NA		Hydrocortisone (300 mg/day) continuous infusion for 6 days	Mortality; SOFA score	
Cicarelli et al. (29)	RCT	S	11/2004–12/2005	29/14	CS: 69 PC: 61	16/13	Septic shock	NA	NA		Dexamethasone (0.2 mg/kg) given 3 times at 36 h	SOFA score 7-day mortality; 28-day mortality lactate evolution	
Aboab et al. (18)	RCT	S	NA	23/10	CS: 55 PC: 56	9/14	Septic shock	Sepsis-1	NA		Hydrocortisone bolus (50 mg) 6 h and fludrocortisone (50 μg) for 7 days	Blood pressure, etc.	
Annane et al. (20)	RCT	M	01/2006–01/2009	518/264	CS: 63.9 PC: 64.3	195/323	Septic shock	NA	NA		Hydrocortisone 50 mg/6 h; fludrocortisone orally in 50 µg tablets/day, each for 7 days	In-hospital mortality mechanical ventilation-free within 28 days; length of stay in ICU	
Yildiz et al. (66)	RCT	S	04/2005–05/2008	55/27	CS: 75 PC: 64	36/19	Sepsis	Sepsis-1	Within 24 h after admission		Prednisolone intravenous boluses 3 times daily at 6 a.m. (10 mg), 2 p.m. (5 mg), and 10 p.m. (5 mg) for 10 days	28-day mortality; hospital stay	
Meduri et al. (46)	RCT	M	04/1997–04/2002	91/63	CS: 59.1 PC: 54.5	44/47	ARDS and sepsis	NA	NA		Methylprednisolone loading dose of 1 mg/kg, followed by continuous infusion of 1 mg/kg per day then 0.5 mg/kg per day from 15–21 days	Length of ICU stay; hospital stay; ICU mortality; hospital mortality	
Snijders et al. (55)	RCT	S	08/2005–07/2008	213/104	CS: 63.0 PC: 64.0	89/124	Sepsis and CAP	NA	After randomization		Prednisolone (40 mg) intravenous once daily for 7 days	Treatment failure at 7 and 30 days; treatment cure at 7 and 30 days	
Meijvis et al. (48)	RCT	M	11/2007–09/2010	304/151	CS: 64.5 PC: 62.5	133/171	Sepsis and CAP	NA	Within a maximum of 12 h from admission		Dexamethasone (5 mg) intravenously for 4 days	Length of hospital stay; hospital mortality; adverse events	
Mirea et al. (49)	RCT	S	NA	112/54	CS (200): 64.3 CS(300): 65.1	NA	Septic shock	NA	NA		Hydrocortisone 50 mg intravenous bolus per 6 h or 200 mg per day as a continuous infusion for a maximum of 7 days	Mean serum sodium values over 7 days; short-term mortality, etc.	
Rezk et al. (17)	RCT	S	10/2011–10/2012	27/18	NA	4/23	Sepsis and ARDS	NA	NA		1 mg/kg methylprednisolone, followed by continuous infusion of 1 mg/kg per day from day 1 to 14, 0.5 mg/kg per day from day 15 to 21, 0.25 mg/kg per day from day 22–25, and 0.125 mg/kg per day from day 26 to 28	Mortality; extubation from mechanical ventilation	
Gordon et al. (34)	RCT	M	10/2010–03/2012	61/31	CS: 61 PC: 60	25/36	Septic shock	Sepsis-1	NA		Hydrocortisone (50 mg) every 6 h for the first 5 days, 50 mg every 12 h for the next 3 days	Mortality; organ failure-free days	
Tongyoo et al. (62)	RCT	S	12/2010–12/2014	197/98	CS: 64.5 PC: 64.3	96/101	Sepsis and ARDS	Sepsis-1	Within 12 h		Hydrocortisone (50 mg) every 6 h for 7 days	28-day mortality; mechanical ventilation-free days; 60-day mortality; adverse events	
Blum et al. (24)	RCT	M	12/2009–05/2014	800/400	CS: 74 PC: 73	NA	CAP	NA	NA		Prednisone 50 mg per day for 7 days	All-cause mortality 30 and 180 days	
Oppert et al. (51)	RCT	S	NA	41/18	CS: 59 PC: 47	9/32	Septic shock	Sepsis-1	After inclusion		Hydrocortisone bolus (50 mg), followed by continuous infusion of 0.18 mg/kg per hour up to cessation of vasopressor for ≥1 h	Vasopressor-free time; 28 days survival; SOFA score; adrenal reserve
Angus et al. (19)	RCT	M	03/2020–06/2020	379/278	CS: 60.4 PC: 65.9	111/273	Severe COVID-19	NA	NA		Intravenous hydrocortisone (50 mg or 100 mg every 6 h) for 7 days	Organ support-free and mortality within 21 days
Torres et al. (63)	RCT	M	06/2004–02/2012	120/61	CS: 64.5 PC: 66.1	46/74	CAP and sepsis	NA	Within 36 h of hospital admission		Methylprednisolone intravenous bolus of 0.5 mg/kg/12 h for 5 days started within 36 h of hospital admission	Length of ICU and hospital; in-hospital mortality
Gordon et al. (35)	RCT	M	02/2013–05/2015	409/202	66	171/238	Septic shock	Sepsis-1	After inclusion		Hydrocortisone (50 mg) every 6 h for the first 5 days	Mortality; serious adverse events
Edalatifard et al. (32)	RCT	S	04/2020–06/2020	62/34	CS: 55.8 PC: 61.7	23/39	Severe COVID-19	NA	After inclusion		Methylprednisolone intravenous injection, 250 mg/day for 3 days	Time of clinical improvement or death
Keh et al. (41)	RCT	M	01/2009–08/2013	353/177	CS: 65.5 PC: 64.6	124/229	Severe sepsis	Sepsi-2	NA		Hydrocortisone bolus (50 mg) followed by a continuous infusion of 200 mg daily for 3 days	Mortality in ICU or hospital; adverse events
Doluee et al. (58)	RCT	S	08/2014–04/2015	160/NA	NA	NA	Septic shock	NA	NA		Hydrocortisone (50 mg intravenous bolus every 6 h for 7 days)	28-day mortality shock termination
Nafae et al. (50)	RCT	S	NA	80/NA	NA	NA	Severe CAP	NA	NA		Hydrocortisone 200 mg as intravenous bolus followed by infusion at 10 mg/h for 7 days	In-hospital mortality; serious adverse events
Dequin et al. (31)	RCT	M	03/2020–06/2020	149/76	CS: 63.1 PC: 66.3	45/104	Severe COVID-19	NA	NA		Hydrocortisone at aninitial dose of 200 mg/day continued at 200 mg/day until day 7	Duration of mechanical ventilation; hospital length of stay
Meduri et al. (47)	RCT	S	NA	80/NA	NA	NA	Sepsis	NA	Severe sepsis <48 h ICU entry		Hydrocortisone as a bolus of 300 mg	Mortality at 7 days and at 28 days, etc.
Tandan et al. (59)	RCT	S	NA	51	51	NA	Septic shock and adrenal insufficiency	NA	NA		Hydrocortisone (stated low dose but actual dose and duration NR)	28-day mortality; the survival of hospital discharge
Hyvernat et al. (39)	RCT	M	11/2008–07/2010	122/63	CS(200): 64.3 CS(300): 65.1	80/42	Septic shock	Sepsis-2	When patients presenting septic shock		Hydrocortisone 50 mg/6 h for 5 days	28 days all-cause mortality; free of vasopressor; free of mechanical ventilation
Tomazini et al. (61)	RCT	M	04/2020–07/2020	299/151	CS: 60.1 PC: 62.7	112/187	COVID-19-associated ARDS	NA	NA		20 mg dexamethasone for 5 days, 10 mg dexamethasone for 5 days or until ICU discharge	Ventilator-free and 28 days all-cause mortality; ICU-free days
Hu et al. (37)	RCT	S	02/2007–01/2009	77/34	CS: 56 PC: 54	48/29	Septic shock	Sepsis-2	After randomization		Hydrocortisone 50 mg/6 h for the first 7 days, 50 mg every 8 h for the next 3 days	Mortality; length of ICU stay
Sabry et al. (53)	RCT	M	07/2010–01/2011	80/40	63	22/58	Sepsis and CAP	NA	NA		Hydrocortisone bolus (200 mg) followed by intravenous dose of 300 mg daily for 7 days	Duration of the mechanical ventilation

RCT, randomized controlled trial; M, multicenter; S, single-center; ARDS, acute respiratory distress syndrome; CS, corticosteroids; PC, placebo or control; ICU, intensive care unit; NA, not acquired; MODS, multiple organ dysfunction syndrome; SOFA, sepsis-related organ failure assessment; IL, interleukin; COVID-19, coronavirus disease 2019; CAP, community-acquired pneumonia.

### Primary Outcome

**Figure 2:**
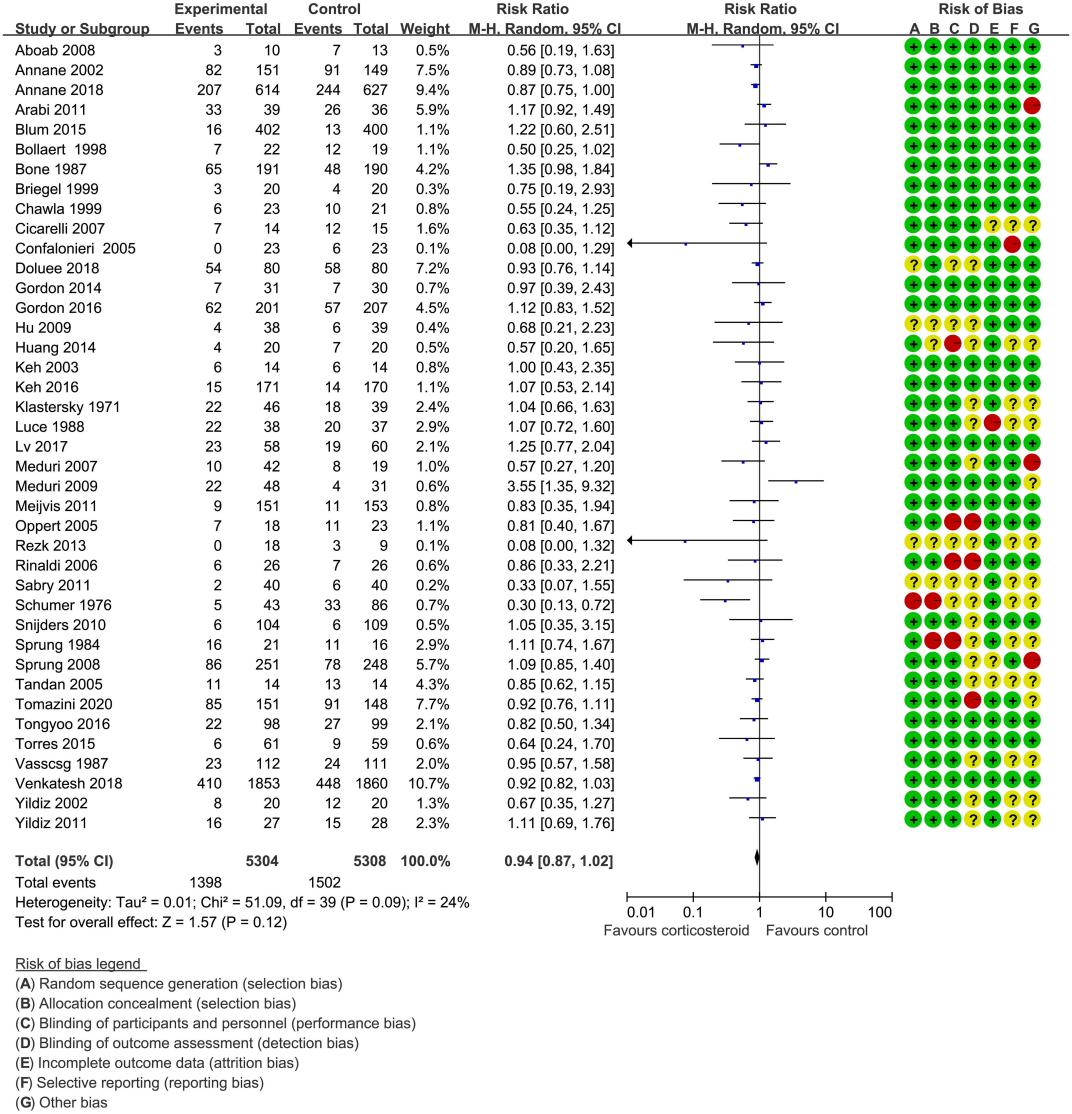
The 28-day mortality of patients with sepsis based on the corticosteroids treatment. The pooled effects in the forest plot were calculated by the M-H method with the random-effects model.

**Figure 3:**
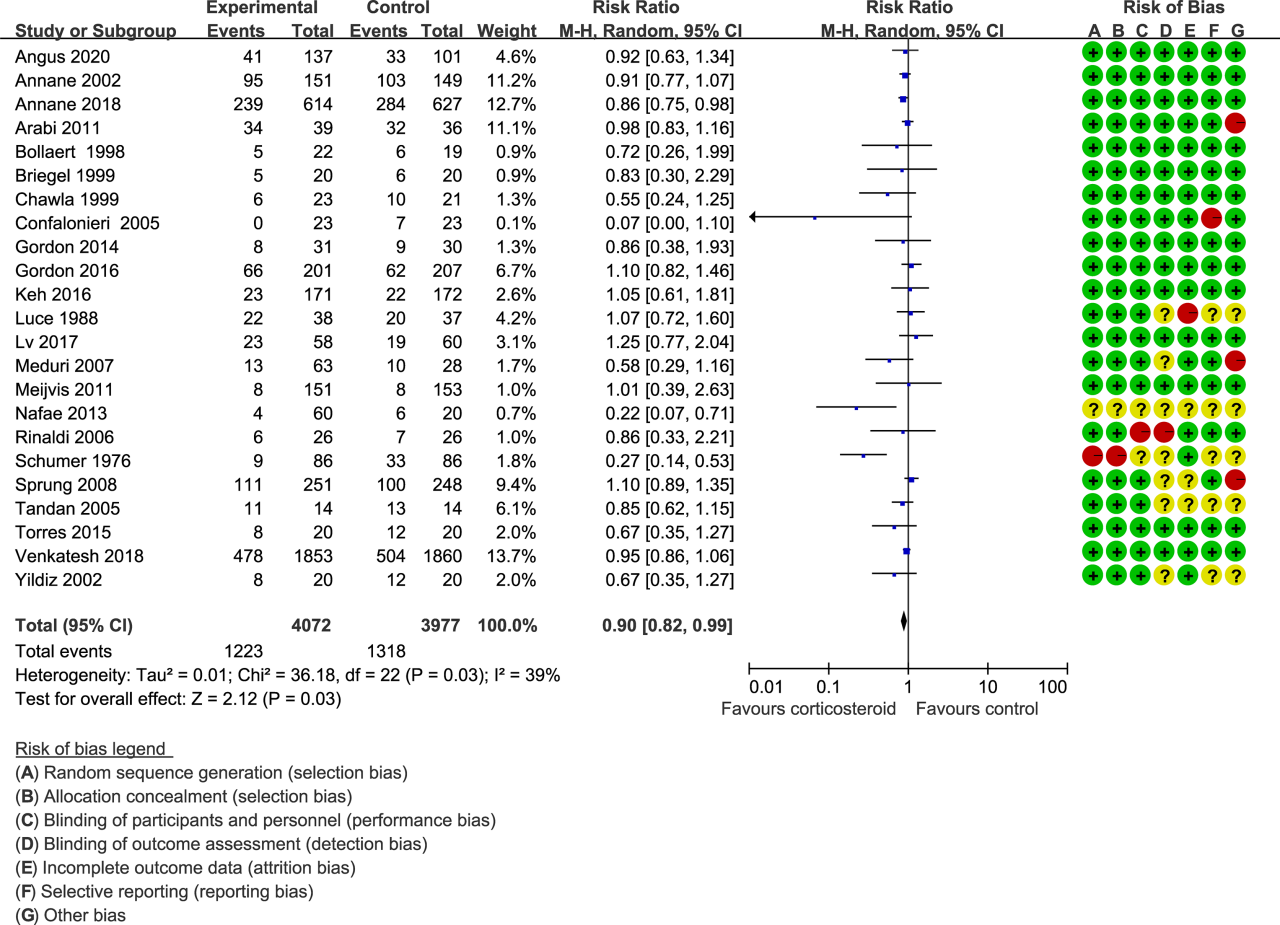
In-hospital mortality of patients with sepsis based on the corticosteroids treatment. The pooled effects in the forest plot were calculated by the M-H method with the random-effects model.

**Figure 4:**
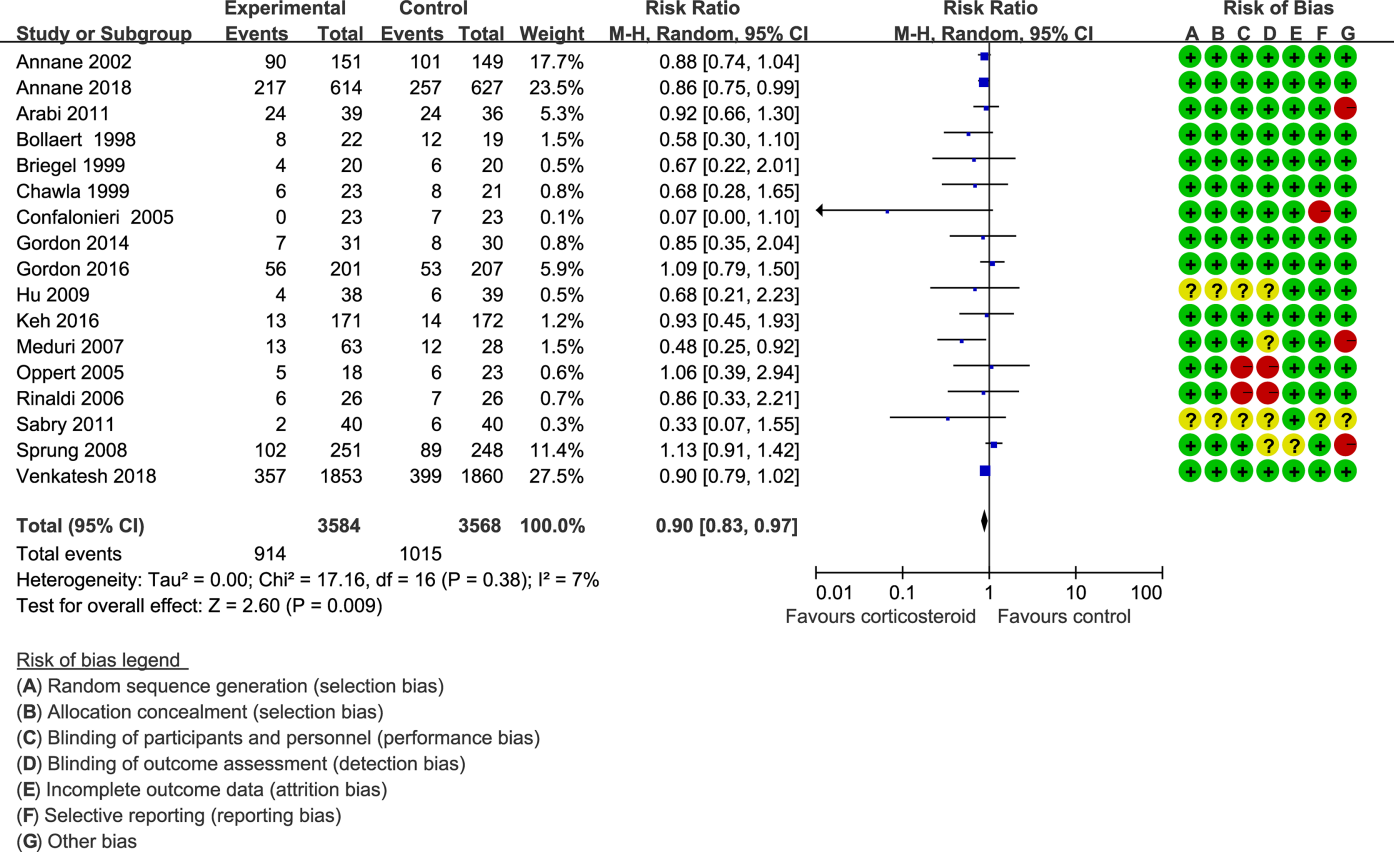
ICU mortality of patients with sepsis based on the corticosteroids treatment. The pooled effects in the forest plot were calculated by the M-H method with the random-effects model.

Forty trials (17, 18, 21–30, 34–38, 40–42, 44–48, 51–59, 61–66) (10,612 patients), 23 trials (21–23, 25, 27, 28, 30, 33–35, 41, 44–48, 50, 52, 56, 59, 63–65) (11,579 patients), and 17 trials (21–23, 25, 27, 28, 30, 34, 35, 37, 41, 46, 52, 53, 56, 63, 64) (7,175 patients) were included in this meta-analysis for assessing the 28-day mortality, in-hospital mortality, and ICU mortality, respectively. We used the random-effects model with RRs to assess the pooled results. Corticosteroids therapy showed no difference in the 28-day mortality (RR, 0.94; 95% CI, 0.87–1.02; evidence rank, moderate; **Figure 2**), with low heterogeneity among the trials (*I* ^2^ = 24%). However, corticosteroids treatment resulted in a significant decrease in the in-hospital mortality (RR, 0.90; 95% CI, 0.82–0.99; evidence rank, moderate; **Figure 3**) and ICU mortality (RR, 0.90; 95% CI, 0.83–0.97; evidence rank, high; **Figure 4**) with low heterogeneity (*I* ^2^ = 39% and *I* ^2^ = 7%, respectively). The Funnel plot and Egger test showed no publication bias in the 28-day mortality (*p* = 0.11), but in-hospital mortality (*p* = 0.028) and ICU mortality (*p* = 0.054) showed potential publication bias (**Supplementary Figures 1**–**3**). The results of sensitivity analysis showed that the models of the 28-day mortality, in-hospital mortality, and ICU mortality were credible (**Supplementary Figures 4**–**6**). Furthermore, L’Abbé plot reported that the mortality in the placebo group increased significantly than the corticosteroids group, suggesting the potential effects of corticosteroids in patients with sepsis (**Supplementary Figures 7**–**9**).

### Secondary Outcomes

**Supplementary Figures 10**–**22** present the assessment of the secondary outcomes. Corticosteroids achieved a small reduction in length of stay in hospital (MD, −1.38; 95% CI, −2.28 to −0.49; *I* ^2^ = 5%; evidence rank, high), SOFA scores at day 7 (MD, −0.90; 95% CI, −1.72 to −0.09; *I* ^2^ = 93%; evidence rank, low), and time to resolution of shock (MD, −1.35; 95% CI, −1.79 to −0.92; *I* ^2^ = 68%; evidence rank, low) for patients with sepsis. Conversely, corticosteroids resulted in higher risk of hypernatremia (RR, 1.51; 95% CI, 1.10–2.07; *I* ^2^ = 0%; evidence rank, moderate) and hyperglycemia (RR, 1.19; 95% CI, 1.10–1.29; *I* ^2^ = 49%; evidence rank, high). Furthermore, corticosteroids increased the vasopressor-free days (MD, 1.93; 95% CI, 0.76–3.09; *I* ^2^ = 0%; evidence rank, moderate), ventilation-free time (MD, 1.46; 95% CI, 0.27–2.65; *I* ^2^ = 21%; evidence rank, moderate), and shock reversal at day 7 (RR, 1.16; 95% CI, 1.06–1.27; *I* ^2^ = 72%; evidence rank, moderate) and day 28 (RR, 1.07; 95% CI, 1.01–1.13; *I* ^2^ = 12%; evidence rank, moderate). Additionally, corticosteroids achieve no reduction in the long-term mortality (>60 days) (RR, 0.96; 95% CI, 0.88–1.05; *I* ^2^ = 54%; evidence rank, low), length of stay in ICU (MD, −0.89; 95% CI, −1.80–0.03; *I* ^2^ = 47%; evidence rank, moderate), superinfection (RR, 1.06; 95% CI, 0.92–1.22; *I* ^2^ = 13%; evidence rank, moderate), and gastroduodenal bleeding (RR, 1.07; 95% CI, 0.85–1.36; *I* ^2^ = 0%; evidence rank, high).

The Funnel plot and Egger test showed no publication bias in the length of stay in hospital (*p* = 0.99), SOFA scores at day 7 (*p* = 0.86), hyperglycemia (*p* = 0.98), the shock reversal at day 7 (*p* = 0.285), length of stay in ICU (*p* = 0.334), superinfection (*p* = 0.231), gastroduodenal bleeding (*p* = 0.867), and shock reversal at day 28 (*p* = 0.414) (**Supplementary Figures 23**–**30**). The results of the sensitivity analysis showed that the models of the abovementioned outcomes, including length of stay in hospital, SOFA scores at day 7, hyperglycemia, shock reversal at day 7, length of stay in ICU, superinfection, gastroduodenal bleeding, and shock reversal at day 28 were credible (**Supplementary Figures 31**–**38**).

Importantly, the risk of bias was reported in the first plot of each outcome, and the evidence rank is shown in **Table 2**.

**Table 2:** The findings and evidence rank of the included studies in patients with sepsis.

Pooled results	No. of Patients (No. of Studies)	Relative Effect, RR, or MD (95% CI)	Heterogeneity *I*^2^,%	Absolute effect (95%CI)	Evidence rank
**Primary outcomes**					
28 d mortality	10,612 (40)	0.94 (0.87, 1.02)	24	17 fewer per 1000 (from 37 fewer to 6 more)	Moderate^1^
In-hospital mortality	8049 (23)	0.90 (0.82, 0.99)	39	33 fewer per 1000 (from 3 fewer to 60 fewer)	Moderate^1^
ICU mortality	7,152 (17)	0.90 (0.83,0.97)	7	28 fewer per 1000 (from 9 fewer to 48 fewer)	High
**Secondary outcomes**					
Long-term mortality	6,254 (9)	0.96 (0.88, 1.05)	54	24 fewer per 1000 (from 48 fewer to 20 more)	Low^2,3^
Shock reversal at 7 d	6,738 (16)	1.16 (1.06,1.27)	72	105 more per 1000 (from 39 more to 178 more)	Moderate^2^
Shock reversal at 28 d	2,526 (12)	1.07 (1.01,1.13)	12	48 more per 1000 (from 7 fewer to 89 more)	Moderate^2^
Gastroduodenal bleeding	5,128 (24)	1.07 (0.85,1.36)	0	3 more per 1000 (from 7 fewer to 17 more)	High
Superinfection	5,375 (24)	1.06 (0.92, 1.22)	13%	10 more per 1000 (from 13 fewer to 36 more)	Moderate^2^
Hypernatremia	4,569 (3)	1.51 (1.10,2.07)	0	12 more per 1000 (from 2 more to 24 more)	Moderate^2^
Hyperglycemia	8,787 (20)	1.19 (1.10,1.29)	49%	49 more per 1000 (from 24 more to 76 more)	High
Vasopressor-free days	1,316 (2)	1.93 (0.76, 3.09)	0	1.93 more per 1000 (from 0.76 more to 3.09 more)	Moderate^2^
Ventilation-free days	1,812 (4)	1.46 (0.27, 2.65)	21	1.46 more per 1000 (from 0.27 more to 2.65 more)	Moderate^2^
Length of stay in hospital	8,383 (19)	-1.38(-2.28, -0.49)	5	1.38 fewer per 1000 (from 2.28fewer to 0.49 fewer)	High
Length of stay in ICU	8,166 (22)	-0.89 (-1.80, 0.03)	47	0.89 fewer per 1000 (from 1.8 fewer to 0.03 more)	High
Time to resolution of shock	4,091 (5)	-1.35(-1.79, -0.92)	68	1.35 fewer per 1000 (from 1.79 fewer to 0.92 fewer)	Low^2,3^
SOFA score at day 7	3,076 (13)	-0.90 (-1.72, -0.09)	93	0.9 fewer per 1000 (from 1.72 fewer to 0.08 fewer)	Low^2,3^

RR, risk ratio; MD, mean difference; ICU, intensive care unit.

^1^Inconsistencies. ^2^Imprecisions. ^3^Risk of bias.

### Subgroup Analysis

We performed subgroup analysis based on the sepsis subtype or type of corticosteroids used for the primary outcomes or *I* ^2^ >75% in the secondary outcomes with more than 10 trials for each outcome. The results of the subgroup analysis showed no effect on the 28-day mortality; however, the in-hospital and ICU mortality were significantly improved in the hydrocortisone plus fludrocortisone treatment and in the patients with septic shock, sepsis, and community-acquired pneumonia (**Supplementary Figures 39**–**44**). Moreover, the result of the subgroup in SOFA scores at day 7 represented that the main original heterogeneity may be from the trials with smaller samples who were given hydrocortisone treatment or trials on patients with sepsis shock (**Supplementary Figures 45** and **46**). Additionally, the subgroup based on the patients that were recruited into the surgical, medical, or surgical/medical ICU showed that corticosteroids were not associated with a 28-day mortality, SOFA scores at day 7, and in-hospital mortality but were related to lower ICU mortality in surgical/medical ICU patients (**Supplementary Table 3**). Importantly, the subgroup in the corticosteroids based on catecholamine use for qualitative analysis showed that 19 RCTs (21–23, 25, 28, 29, 34, 35, 39–41, 43, 45, 51, 56–58, 60, 61) reported 28-day mortality, but it was not associated with the reduced 28-day mortality, no matter what the corticosteroids dose. Moreover, 11 RCTs (21–23, 25, 27, 28, 34, 35, 41, 45, 56) showed in-hospital mortality, 8 RCTs (21–23, 34, 35, 41, 45, 56) of which reported that the dose of corticosteroids was 200 mg/day or 50 mg every 6 h; only 1 (21) showed that corticosteroids may be associated with lower in-hospital mortality. Eleven RCTs (21–23, 25, 27, 28, 34, 35, 41, 51, 56) reported ICU mortality, seven studies (21–23, 34, 35, 41, 56) of which reported the corticosteroids dose was 200 mg/day or 50 mg every 6 h; only one (21) study showed that corticosteroids may be associated with the lower ICU mortality. Furthermore, three RCTs (25, 27, 28) showed that the corticosteroids dose was more than 200 mg/day; corticosteroids was not associated with the ICU mortality. However, two RCTs (21, 23) showed that corticosteroids dose of 200 mg/day or 50 mg every 6 h may reduce the time of vasopressors use.

## Discussion

This meta-analysis included 50 RCTs (12,304 patients) and demonstrated that corticosteroids failed to improve the 28-day and long-term mortality; however, there was a small reduction in the in-hospital mortality and ICU mortality. To our knowledge, this systematic review and meta-analysis is the most comprehensive review of many new RCTs; the precision of the pooled effect estimates how sepsis could be increased substantially.

We found that the corticosteroids therapy for sepsis increased the incidence of the vasopressor-free days, ventilation-free time, shock reversal at days 7 and 28, and adverse events, such as hyperglycemia and hypernatremia. Corticosteroids were associated with a decreased risk of the time for shock resolution and length of stay in the hospital. However, our study failed to report a decreased risk of corticosteroids on the length of ICU stay and adverse events, such as superinfection and gastroduodenal bleeding. Ascertainment of the adverse events in the eligible trials was also vulnerable, which may induce the evidence rank to be low. Moreover, a quantitative analysis for the effect of the time of corticosteroids administration on septic patients was made. The effect of the different time of corticosteroid administration on septic patients cannot be compared because the time of corticosteroid administration was indistinct in the included studies. Therefore, further clinical studies should explore the time of corticosteroid administration for septic patients and ensure whether it is the same as antibiotics, which is the earlier the better.

Subgroup analyses in this review showed that the results did not identify any credible effect of modification in sepsis subtype and type of corticosteroids used. Much evidence comes from the trials with hydrocortisone or methylprednisolone treatment. Our subgroup analysis results showed that the efficacy of corticosteroids on in-hospital, ICU, and short-time mortality was mainly due to the hydrocortisone plus fludrocortisone.

Mechanistically, corticosteroids could inhibit the nuclear factor kappa B (NF-κB) activation and the extensive inflammatory factors release, finally improving the inflammatory response of sepsis or pneumonia. Our previous studies reported that corticosteroids were associated with a decreased risk of ARDS and length of the disease in patients with CAP (67). Previous reviews have assessed the efficacy and safety of corticosteroids in patients with sepsis. Unfortunately, the conclusions were contradictory owing to the small number of trials included. One meta-analysis included 20 RCTs and showed no reduction in the 28-day mortality, hospital mortality, and ICU mortality in patients with severe sepsis and sepsis shock on corticosteroids treatment (68). Subsequently, a Cochrane systematic review further conducted to search the effect of corticosteroids on mortality of patients with sepsis, including a total of 33 RCTs, found a small reduction in 28-day mortality on the corticosteroids treatment (69). Simultaneously, another study included 35 RCTs and showed a converse result that corticosteroids failed to decrease the mortality (70). In 2018, Rochwerg et al. (7) examined 42 RCTs including 10,194 patients, wherein corticosteroids achieved no reduction in the short-term (28–31 days) mortality and may have a little effect on the long-term mortality. In 2019, Fang et al. (71) included 37 RCTs; this trial suggested that corticosteroids use was associated with a decrease in the 28-day mortality, ICU mortality, and in-hospital mortality. In parallel, Annane et al. (6) published a Cochrane systematic review on 40 RCTs and achieved a reduction in the 28-day mortality in patients with sepsis on the corticosteroids therapy.

The results of this meta-analysis showed that corticosteroids treatment failed to improve the 28-day mortality, in contrast with results from the previous meta-analysis. The difference in part may be due to the result reported by Annane et al. (6), in which corticosteroids therapy showed an increased risk of 28-day mortality, while the CI contained the null effect line, suggesting that corticosteroids had no effect on sepsis based on the statistics. More importantly, we included four RCTs about severe COVID-19 and showed that there was no significant difference in 28-day mortality with corticosteroids use. The data were extracted from the latest RCTs and may have helped in reinforcing the conclusions, decreasing the heterogeneity among the studies, and improving the precision with more comprehensive assessment for the therapeutic effects of corticosteroids treatment.

In this meta-analysis, the result of the qualitative analysis showed that 200 mg/day or less may have a clinical benefit, such as increasing the vasopressor-free time, improving tissue oxygen supply, and restoring circulatory homeostasis in catecholamine-dependent septic shock (21, 23, 37). More importantly, the earlier study (25) reported that supraphysiological doses of hydrocortisone could improve hemodynamics and enhance the vascular sensitivity to catecholamines, thereby reducing the dose of catecholamine (dopamine >10 µg/kg/min) in the patients with septic shock. The subsequent studies (40) also showed that under the dose of dopamine ≥6 µg/kg/min, low-dose hydrocortisone could be a better maintenance of hemodynamics by increasing vascular sensitivity to catecholamines. Furthermore, Ibarra-Estrada et al. (72) suggested that compared with bolus infusion of hydrocortisone, continuous infusion may restore the vascular sensitivity to catecholamines better. Similarly, an experimental study (73) found that fludrocortisone combined with hydrocortisone therapy dose dependently increased phenylephrine with cumulative increasing concentrations, which caused concentration-dependent contraction of isolated mesenteric arteries from septic rats. Contrarily, a prospective cohort study (74) showed that after hydrocortisone therapy, there was a significant reduction in norepinephrine in survivors, whereas higher catecholamine dosages were required for the non-survivors. However, a latest retrospective cohort study (75) showed that higher norepinephrine (24.6 mcg/min) in early hydrocortisone therapy could improve reduction in ICU mortality compared with the late hydrocortisone therapy with norepinephrine (21.3 mcg/min) in patients with sepsis shock. Based on the abovementioned results, the potential mechanisms of corticosteroids restoring the vascular sensitivity to catecholamines have been reported as follows: (1) in the septic shock, as the excess production of nitric oxide causes host catecholamine resistance (76, 77), corticosteroids could inhibit inducible NOS formation and production restoring the vascular sensitivity to catecholamines; (2) desensitization and/or downregulation of β-adrenergic receptors (78) and possibly α-adrenergic receptors (79) maybe due to the downregulation by endogenous catecholamine production in septic shock, whereas the corticosteroids may reverse receptor desensitization (80) and further allow reduced catecholamine dosage (25). Given that the evidence of the relationship between catecholamines and corticosteroids is currently inconsistent, future clinical studies should be conducted to further research the dependence of catecholamine administration on the effect of cortisone administration.

Additionally, to explore which septic patients were more responsive to the corticosteroids therapy, the ICU subgroup analysis after the type of disease and corticosteroids subgroup was conducted. The results showed that with corticosteroids use, there was no difference in the 28-day mortality, in-hospital mortality, and SOFA scores at day 7 among the surgical ICU, medical ICU, and surgical/medical ICU, but there was lower ICU mortality in patients with sepsis from surgical/medical ICU. The results of the subgroup analysis may not provide useful information mainly because ICU description is too vague in the included studies. Thus, details cannot be determined. Therefore, future clinical research should distinguish patients based on ICU type (e.g., surgical or medical ICU) to explore which sepsis primary cause is the corticosteroids therapy effective.

Corticosteroids have already been used for adjuvant therapy in sepsis for more than half a century. However, credible evidence is still lacking to guide the choice of patients, the time of corticosteroids administration, or the dose of corticosteroids for catecholamine-dependent patients. With the definition of sepsis that varies from sepsis-1.0 to sepsis-3.0, the accuracy of sepsis diagnosis has significantly improved. However, corticosteroids use also varied from sepsis-1.0 to sepsis-3.0. Specifically, only patients with septic shock used corticosteroids and suggested the use of flumetasone (50 µg/day) in sepsis-1.0 (81). The use of hydrocortisone was suggested only in children with suspected or confirmed absolute adrenal insufficiency, which was a more stringent use of corticosteroids compared with sepsis-1, in sepsis-2.0 (82), and the use of hydrocortisone (200 mg/day) was suggested only in patients with refractory septic shock wherein appropriate fluid resuscitation and vasopressor therapy cannot restore hemodynamic stability in sepsis-3.0 (3), The proposals from sepsis-1.0, sepsis-2.0, and sepsis-3.0 lack credible evidence to support the clinical use of corticosteroids. Analysis of all relevant data from available RCTs showed that the effect of corticosteroids therapy for septic patients was not consistent. However, the latest studies showed that corticosteroids may not reduce mortality in septic patients compared with the control group. Importantly, this study suggests that corticosteroids administration may not reduce the 28-day mortality, long-term mortality, and length of ICU stay but may be associated with ICU mortality, in-hospital mortality, length of hospital stay, SOFA scores at day 7, and time to shock resolution, and increased shock reversal at days 7 and 28 and vasopressor- and ventilation-free days. Furthermore, this study suggested that corticosteroids may be an effective therapy with a low dose and long-term course. However, future studies need to appropriately study the time of corticosteroids administration, the primary infection source, and dose of corticosteroids use for septic shock patients who are dependent on catecholamine in the treatment of sepsis.

This meta-analysis has several strengths. First, this study is the most comprehensive trial to assess the efficacy of corticosteroids treatment on patients with sepsis to date. Second, we performed a thorough literature search including unpublished sources, using the GRADE methodology, to evaluate the evidence rank in overall RR, a predefined illustration of potential effect variables including direction of effect and subsequent subgroup analysis to search the effect variables, and illustration including the relative and absolute effects. Third, the primary outcomes showed low or no heterogeneity among the studies, suggesting that the results were not variable. Furthermore, the heterogeneity of SOFA scores on day 7 was high, and the subgroup analysis showed that the source of heterogeneity may be the inclusion of trials with small size on patients who were given hydrocortisone treatment. Finally, the results of the sensitivity analysis for this study suggested that these conclusions were robust and reliable.

However, this meta-analysis also has certain limitations, including the significant methodological or clinical heterogeneity among the included studies, especially with respect to the SOFA score on day 7. All the included RCTs enrolled patients with sepsis as per the previous sepsis definition criteria; however, we do not know whether the efficacy and safety of corticosteroids would change using the Sepsis-3 definition criteria. Hence, the defined mortality may be essential, but the certainty is limited due to the imprecision of the included studies.

## Conclusions

This is the most comprehensive systematic review and meta-analysis to describe the efficacy and safety of corticosteroids in patients with sepsis. The findings demonstrate that corticosteroids failed to reduce the 28-day, 90-day, and long-term mortalities; however, they could reduce the in-hospital and ICU mortalities. Importantly, our subgroup analyses results indicated that this efficacy of corticosteroids in patients with sepsis may be associated with the hydrocortisone plus fludrocortisone treatment. Therefore, the results suggest that corticosteroids could not improve the 28-day mortality in adult patients with sepsis.

## Data Availability Statement

The original contributions presented in the study are included in the article/**Supplementary Material**. Further inquiries can be directed to the corresponding authors.

## Author Contributions

All the authors contributed equally to the work presented in this article. TS, HuL conceived the idea of this study. XD, HoL, and GS contributed to the data extraction. SH, RZ, and XD computed and evaluated the pooled outcomes. HuL and SH contributed to the study protocol and wrote the article. QK and TS revised the article. QK and TS had full access to all of the data, and the final responsibility for the decision to submit this article for publication. All authors contributed to the article and approved the submitted version.

## Funding

This study was supported by the United Fund of National Natural Science Foundation of China (Grant No. U2004110) and Leading Talents Fund in Science and Technology Innovation in Henan Province (Grant No. 194200510017).

## Conflict of Interest

The authors declare that the research was conducted in the absence of any commercial or financial relationships that could be construed as a potential conflict of interest.

## Publisher’s Note

All claims expressed in this article are solely those of the authors and do not necessarily represent those of their affiliated organizations, or those of the publisher, the editors and the reviewers. Any product that may be evaluated in this article, or claim that may be made by its manufacturer, is not guaranteed or endorsed by the publisher.

## Supplementary Material

Supplementary Figure 1 The Funnel plot assessed the potential publication bias of pooled effect in 28-day mortality for corticosteroids *vs*. placebo treatment in patients with sepsis.

Supplementary Figure 2 The Funnel plot assessed the potential publication bias of pooled effect in in-hospital mortality for corticosteroids *vs*. placebo treatment in patients with sepsis.

Supplementary Figure 3 The Funnel plot assessed the potential publication bias of pooled effect in ICU mortality for corticosteroids *vs*. placebo treatment in patients with sepsis.

Supplementary Figure 4 The sensitivity analysis evaluated the robustness of the pooled effect model in 28-day mortality for this meta-analysis.

Supplementary Figure 5 The sensitivity analysis evaluated the robustness of the pooled effect model in in-hospital mortality for this meta-analysis.

Supplementary Figure 6 The sensitivity analysis evaluated the robustness of the pooled effect model in ICU mortality for this meta-analysis.

Supplementary Figure 7 L’Abbe plot according to the corticosteroids therapy. 40 RCTs of corticosteroids and 28-day mortality in patients with sepsis were presented in a L’Abbe plot.

Supplementary Figure 8 L’Abbe plot according to the corticosteroids therapy. 23 RCTs of corticosteroids and in-hospital mortality in patients with sepsis were presented in a L’Abbe plot.

Supplementary Figure 9 L’Abbe plot according to the corticosteroid therapy. 17 RCTs of corticosteroids and ICU mortality in patients with sepsis were presented in a L’Abbe plot.

Supplementary Figure 10 The Forest plot showed the pooled effect of length of stay in hospital in patients with sepsis based on the corticosteroids treatment for this meta-analysis.

Supplementary Figure 11 The Forest plot showed the pooled effect of SOFA scores at day 7 in patients with sepsis based on the corticosteroids treatment for this meta-analysis.

Supplementary Figure 12 The Forest plot showed the pooled effect of time to resolution of shock in patients with sepsis based on the corticosteroids treatment for this meta-analysis.

Supplementary Figure 13 The Forest plot showed the pooled effect of hypernatremia in patients with sepsis based on the corticosteroids treatment for this meta-analysis.

Supplementary Figure 14 The Forest plot showed the pooled effect of hyperglycemia in patients with sepsis based on the corticosteroids treatment for this meta-analysis.

Supplementary Figure 15 The Forest plot showed the pooled effect of vasopressor-free days in patients with sepsis based on the corticosteroids treatment for this meta-analysis.

Supplementary Figure 16 The Forest plot showed the pooled effect of ventilation-free time in patients with sepsis based on the corticosteroids treatment for this meta-analysis.

Supplementary Figure 17 The Forest plot showed the pooled effect of shock reversal at day 7 in patients with sepsis based on the corticosteroids treatment for this meta-analysis.

Supplementary Figure 18 The Forest plot showed the pooled effect of shock reversal at day 28 in patients with sepsis based on the corticosteroids treatment for this meta-analysis.

Supplementary Figure 19 The Forest plot showed the pooled effect of long-term mortality in patients with sepsis based on the corticosteroids treatment for this meta-analysis.

Supplementary Figure 20 The Forest plot showed the pooled effect of length of stay in ICU in patients with sepsis based on the corticosteroids treatment for this meta-analysis.

Supplementary Figure 21 The Forest plot showed the pooled effect of superinfection in patients with sepsis based on the corticosteroids treatment for this meta-analysis.

Supplementary Figure 22 The Forest plot showed the pooled effect of gastroduodenal bleeding in patients with sepsis based on the corticosteroids treatment for this meta-analysis.

Supplementary Figure 23 The Funnel plot assessed the potential publication bias of pooled effect in length of stay in hospital in patients with sepsis for this meta-analysis.

Supplementary Figure 24 The Funnel plot assessed the potential publication bias of pooled effect in SOFA scores at day 7 in patients with sepsis for this meta-analysis.

Supplementary Figure 25 The Funnel plot assessed the potential publication bias of pooled effect in hyperglycemia in-hospital in patients with sepsis for this meta-analysis.

Supplementary Figure 26 The Funnel plot assessed the potential publication bias of pooled effect in the shock reversal at day 7 in patients with sepsis for this meta-analysis.

Supplementary Figure 27 The Funnel plot assessed the potential publication bias of pooled effect in length of stay in ICU in patients with sepsis for this meta-analysis.

Supplementary Figure 28 The Funnel plot assessed the potential publication bias of pooled effect in superinfection in patients with sepsis for this meta-analysis.

Supplementary Figure 29 The Funnel plot assessed the potential publication bias of pooled effect in gastroduodenal bleeding in patients with sepsis for this meta-analysis.

Supplementary Figure 30 The Funnel plot assessed the potential publication bias of pooled effect in shock reversal at day 28 in patients with sepsis for this meta-analysis.

Supplementary Figure 31 The sensitivity analysis evaluated the robustness of pooled effect model in length of stay in hospital for this meta-analysis.

Supplementary Figure 32 The sensitivity analysis evaluated the robustness of pooled effect model in SOFA scores at day 7 for this meta-analysis.

Supplementary Figure 33 The sensitivity analysis evaluated the robustness of pooled effect model in hyperglycemia for this meta-analysis.

Supplementary Figure 34 The sensitivity analysis evaluated the robustness of pooled effect model in shock reversal at day 7 for this meta-analysis.

Supplementary Figure 35 The sensitivity analysis evaluated the robustness of pooled effect model in length of stay in ICU for this meta-analysis.

Supplementary Figure 36 The sensitivity analysis evaluated the robustness of pooled effect model in superinfection for this meta-analysis.

Supplementary Figure 37 The sensitivity analysis evaluated the robustness of pooled effect model in gastroduodenal bleeding for this meta-analysis.

Supplementary Figure 38 The sensitivity analysis evaluated the robustness of pooled effect model in shock reversal at day 28 for this meta-analysis.

Supplementary Figure 39 Subgroup analysis of 28-day mortality of patients with sepsis based on the corticosteroids type.

Supplementary Figure 40 Subgroup analysis of in-hospital mortality of patients with sepsis based on the corticosteroids type.

Supplementary Figure 41 Subgroup analysis of ICU mortality of patients with sepsis based on the corticosteroids type.

Supplementary Figure 42 Subgroup analysis of 28-day mortality of patients with sepsis based on the sepsis subtype.

Supplementary Figure 43 Subgroup analysis of in-hospital mortality of patients with sepsis based on the sepsis subtype.

Supplementary Figure 44 Subgroup analysis of ICU mortality of patients with sepsis based on the sepsis subtype.

Supplementary Figure 45 Subgroup analysis of SOFA scores at day 7 of patients with sepsis based on the corticosteroids type.

Supplementary Figure 46 Subgroup analysis of SOFA scores at day 7 of patients with sepsis based on the sepsis subtype.

## Abbreviations

RR, risk ratio; CI, confidence interval; RCTs, randomized controlled trials; MD, mean difference; CAP, community-acquired pneumonia; ICU, intensive care unit; ARDS, acute respiratory distress syndrome; GRADE, Grading of Recommendations Assessment, Development and Evaluation; SOFA, Sequential Organ Failure Assessment.
