# Corrigendum: Corticosteroids for Treating Sepsis in Adult Patients: A Systematic Review and Meta-Analysis

**DOI:** 10.3389/fimmu.2021.771779

**Published:** 2021-11-05

**Authors:** Huoyan Liang, Heng Song, Ruiqing Zhai, Gaofei Song, Hongyi Li, Xianfei Ding, Quancheng Kan, Tongwen Sun

**Affiliations:** ^1^General ICU, The First Affiliated Hospital of Zhengzhou University, Henan Key Laboratory of Critical Care Medicine, Zhengzhou Key Laboratory of Sepsis, Henan Engineering Research Center for Critical Care Medicine, Zhengzhou, China; ^2^Academy of Medical Sciences, Zhengzhou University, Zhengzhou, China; ^3^College of Bioinformatics Science and Technology, Harbin Medical University, Harbin, China; ^4^Department of Pharmacy, The First Affiliated Hospital of Zhengzhou University, Zhengzhou, China

**Keywords:** corticosteroids, sepsis, mortality, systematic review, meta-analysis

In the original article, there was a mistake in **Figure 2**, **Supplemental Figures 40-41, 43, 45-46** and **Table 2** as published. For **Figure 2**, we mistakenly adopted the fixed effect model, resulting in the difference between the picture and the actual results. In practice, we need to use the random-effect model to calculate the effect quantity and its 95% CIs, which is the most reasonable. In both the method part and the result part, we describe the calculation using the random effect model. For **Supplemental Figures 40-41, 43, 45-46** and **Table 2**, as we reworked all the figures, we made mistakes in uploading in the revised manuscript. The corrected **Figure 2**, **Supplemental Figures 40-41, 43, 45-46** and **Table 2** appear below.

**Figure 2:**
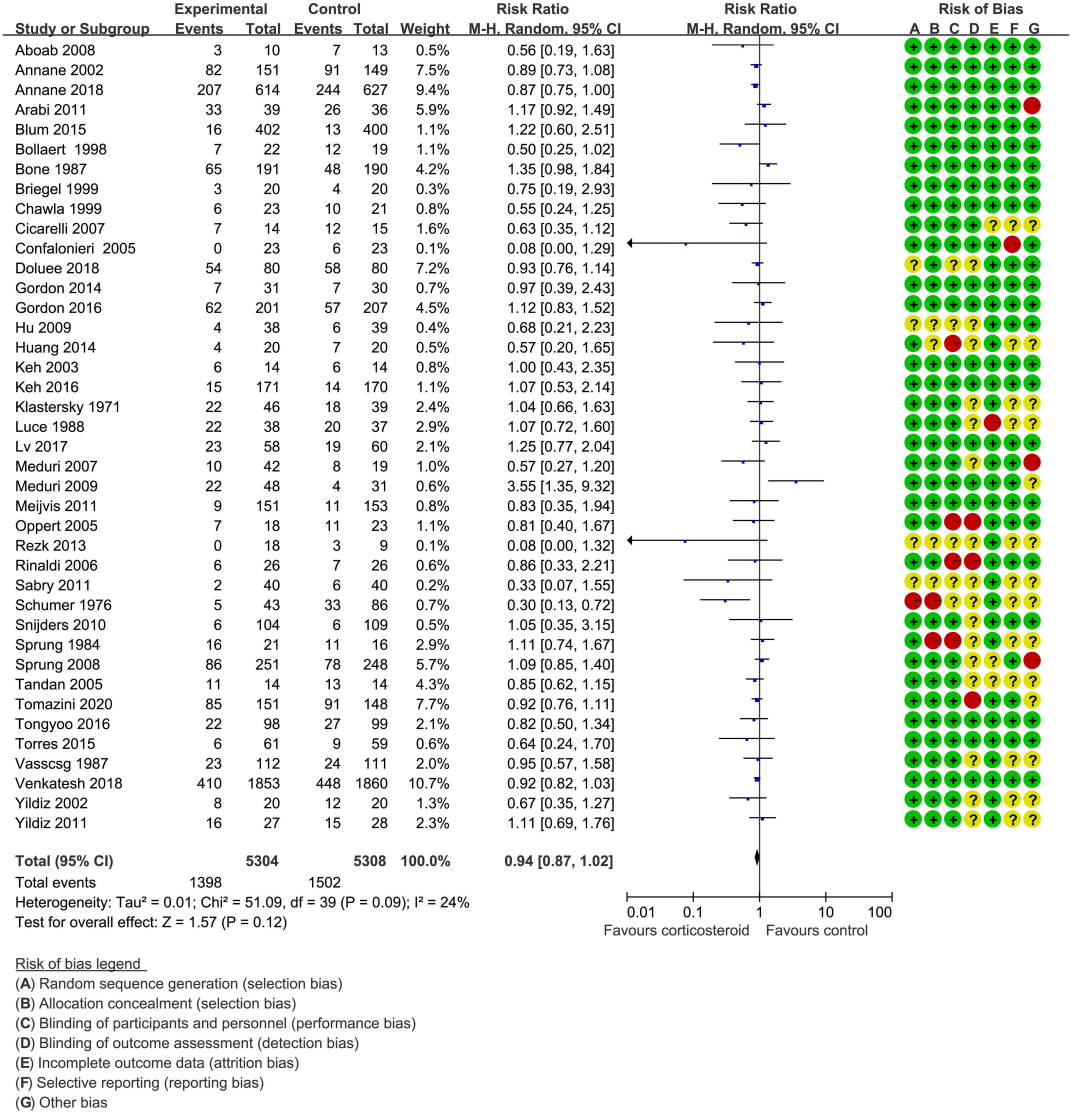
The 28-day mortality of patients with sepsis based on the corticosteroids treatment. The pooled effects in the forest plot were calculated by the M-H method with the random-effects model.

**Table 2:** The findings and evidence rank of the included studies in patients with sepsis.

Pooled results	No. of Patients (No. of Studies)	Relative Effect, RR, or MD (95% CI)	Heterogeneity *I*^2^,%	Absolute effect (95%CI)	Evidence rank
**Primary outcomes**					
28 d mortality	10,612 (40)	0.94 (0.87, 1.02)	24	17 fewer per 1000 (from 37 fewer to 6 more)	Moderate^1^
In-hospital mortality	8049 (23)	0.90 (0.82, 0.99)	39	33 fewer per 1000 (from 3 fewer to 60 fewer)	Moderate^1^
ICU mortality	7,152 (17)	0.90 (0.83,0.97)	7	28 fewer per 1000 (from 9 fewer to 48 fewer)	High
**Secondary outcomes**					
Long-term mortality	6,254 (9)	0.96 (0.88, 1.05)	54	24 fewer per 1000 (from 48 fewer to 20 more)	Low^2,3^
Shock reversal at 7 d	6,738 (16)	1.16 (1.06,1.27)	72	105 more per 1000 (from 39 more to 178 more)	Moderate^2^
Shock reversal at 28 d	2,526 (12)	1.07 (1.01,1.13)	12	48 more per 1000 (from 7 fewer to 89 more)	Moderate^2^
Gastroduodenal bleeding	5,128 (24)	1.07 (0.85,1.36)	0	3 more per 1000 (from 7 fewer to 17 more)	High
Superinfection	5,375 (24)	1.06 (0.92, 1.22)	13%	10 more per 1000 (from 13 fewer to 36 more)	Moderate^2^
Hypernatremia	4,569 (3)	1.51 (1.10,2.07)	0	12 more per 1000 (from 2 more to 24 more)	Moderate^2^
Hyperglycemia	8,787 (20)	1.19 (1.10,1.29)	49%	49 more per 1000 (from 24 more to 76 more)	High
Vasopressor-free days	1,316 (2)	1.93 (0.76, 3.09)	0	1.93 more per 1000 (from 0.76 more to 3.09 more)	Moderate^2^
Ventilation-free days	1,812 (4)	1.46 (0.27, 2.65)	21	1.46 more per 1000 (from 0.27 more to 2.65 more)	Moderate^2^
Length of stay in hospital	8,383 (19)	-1.38(-2.28, -0.49)	5	1.38 fewer per 1000 (from 2.28fewer to 0.49 fewer)	High
Length of stay in ICU	8,166 (22)	-0.89 (-1.80, 0.03)	47	0.89 fewer per 1000 (from 1.8 fewer to 0.03 more)	High
Time to resolution of shock	4,091 (5)	-1.35(-1.79, -0.92)	68	1.35 fewer per 1000 (from 1.79 fewer to 0.92 fewer)	Low^2,3^
SOFA score at day 7	3,076 (13)	-0.90 (-1.72, -0.09)	93	0.9 fewer per 1000 (from 1.72 fewer to 0.08 fewer)	Low^2,3^

RR, risk ratio; MD, mean difference; ICU, intensive care unit.

^1^Inconsistencies. ^2^Imprecisions. ^3^Risk of bias.

In the original article, there was an error.** As the outcomes for Hypernatremia, Hyperglycemia and SOFA at day 7 were revised several times, and the pooled effects were not changed, but the up or low 95% CI had a little change. When we modified, we ignored the modifications in the two places.**

A correction has been made to ***RESULTS*, *Secondary Outcomes of RESULTS*, *Paragraph 1***:

**“****Supplementary Figures 10–22** **present the assessment of the secondary outcomes. Corticosteroids achieved a small reduction in length of stay in hospital (MD, −1.38; 95% CI, −2.28 to −0.49; I^2^ = 5%; evidence rank, high), SOFA scores at day 7 (MD, −0.90; 95% CI, −1.72 to −0.09; I^2^ = 93%; evidence rank, low), and time to resolution of shock (MD, −1.35; 95% CI, −1.79 to −0.92; I^2 =^ 68%; evidence rank, low) for patients with sepsis. Conversely, corticosteroids resulted in higher risk of hypernatremia (RR, 1.51; 95% CI, 1.10–2.07; I^2^ = 0%; evidence rank, moderate) and hyperglycemia (RR, 1.19; 95% CI, 1.10–1.29; I^2 =^ 49%; evidence rank, high).”**

The authors apologize for these errors and state that they do not change the scientific conclusions of the article in any way. The original article has been updated.

## Publisher’s Note

All claims expressed in this article are solely those of the authors and do not necessarily represent those of their affiliated organizations, or those of the publisher, the editors and the reviewers. Any product that may be evaluated in this article, or claim that may be made by its manufacturer, is not guaranteed or endorsed by the publisher.

