# Supplementary material for: Corticosteroids for Treating Sepsis in Adult Patients: A Systematic Review and Meta-Analysis
Source: Front Immunol. 2021 Aug 16;12:709155. doi: 10.3389/fimmu.2021.709155 (PMC8415513; doi:10.3389/fimmu.2021.709155)
Supplement: Supplementary Figure 1 — The Funnel plot assessed the potential publication bias of pooled effect in 28-day mortality for corticosteroids vs. placebo treatment in patients with sepsis. [file DataSheet_1.zip › Data Sheet 2/Supplemental Table 2.DOCX]

**Supplemental Table 2. The detailed search strategy**

| **Electronic databases** | **Search** | **Search strategy** | **Results** |
| --- | --- | --- | --- |
| **Pudmed** | #1 | (((sepsis[MeSH Terms]) OR (sepsis[Title/Abstract])) OR (critically ill patients[Title/Abstract])) OR (critically ill patients[MeSH Terms]) | 217,481 |
|  | #2 | (((((((corticosteroids[MeSH Terms]) OR (corticosteroids[Title/Abstract])) OR (hydrocortisone[Title/Abstract])) OR (steroid[Title/Abstract])) OR (betamethasone[Title/Abstract])) OR (methylprednisolone[Title/Abstract])) OR (prednisolone[Title/Abstract])) OR (dexamethasone[Title/Abstract]) | 511,744 |
|  | #3  #4  #5 | (COVID-19[MeSH Terms]) OR (COVID-19[Title/Abstract])  ((COVID-19[MeSH Terms]) OR (COVID-19[Title/Abstract])) OR ((((sepsis[MeSH Terms]) OR (sepsis[Title/Abstract])) OR (critically ill patients[Title/Abstract])) OR (critically ill patients[MeSH Terms]))  (((COVID-19[MeSH Terms]) OR (COVID-19[Title/Abstract])) OR ((((sepsis[MeSH Terms]) OR (sepsis[Title/Abstract])) OR (critically ill patients[Title/Abstract])) OR (critically ill patients[MeSH Terms]))) AND ((((((((corticosteroids[MeSH Terms]) OR (corticosteroids[Title/Abstract])) OR (hydrocortisone[Title/Abstract])) OR (steroid[Title/Abstract])) OR (betamethasone[Title/Abstract])) OR (methylprednisolone[Title/Abstract])) OR (prednisolone[Title/Abstract])) OR (dexamethasone[Title/Abstract])) | 145,267  360,217  **8,602** |
| **EMBASE** | #1 | ('corticosteroid'/de OR corticosteroid:ab,ti OR hydrocortisone:ab,ti OR betamethasone:ab,ti OR methylprednisolone:ab,ti OR prednisolone:ab,ti OR dexamethasone:ab,ti) AND ([randomized controlled trial]/lim) | 18,790 |
|  | #2 | ('coronavirus disease 2019':ab,ti OR 'coronavirus disease 2019'/de OR 'sepsis'/de OR sepsis:ab,ti OR 'critical illness':ab,ti OR 'critical illness'/de) AND ([randomized controlled trial]/lim) | 16,863 |
|  | #3 | #1 AND #2 | **1,025** |
| **Cochrane CENTRAL** | #1  #2  #3  #4  #5  #6  #7  #8  #9  #10  #11 | MeSH descriptor: [Sepsis] this term only  MeSH descriptor: [Critical Illness] this term only  ("sepsis" or "critically ill patients"):ti,ab,kw  MeSH descriptor: [COVID-19] this term only  ("COVID-19"):ti,ab,kw  #1 OR #2 OR #3 OR #4 OR #5  MeSH descriptor: [Adrenal Cortex Hormones] explode all trees  (corticosteroids):ti,ab,kw OR (hydrocortisone):ti,ab,kw OR (steroid):ti,ab,kw OR (fludrocortisone):ti,ab,kw OR (dexamethasone):ti,ab,kw  (methylprednisolone):ti,ab,kw OR (prednisolone):ti,ab,kw OR (betamethasone):ti,ab,kw  #7 OR #8 OR #9  #6 AND #10 | 2,152  2,394  17,362  467  5,915  19,294  46,502  53,917  13,870  59,024  **1140** |
|  |  |  |  |
|  |  |  |  |
