# Supplementary figures and images for: Corticosteroids for Treating Sepsis in Adult Patients: A Systematic Review and Meta-Analysis
Source: Front Immunol. 2021 Aug 16;12:709155. doi: 10.3389/fimmu.2021.709155 (PMC8415513; doi:10.3389/fimmu.2021.709155)

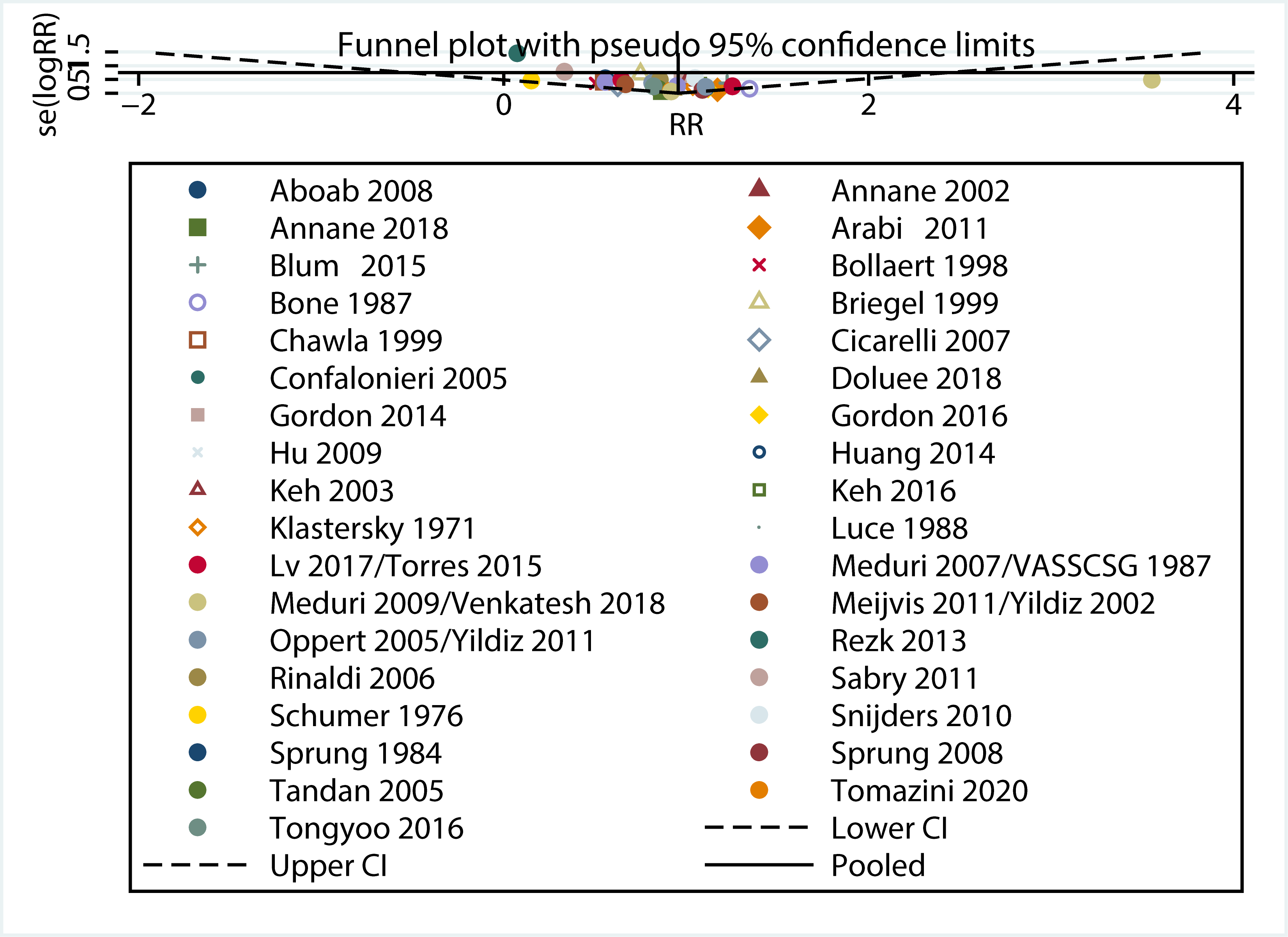

Supplement: Supplementary Figure 1 — The Funnel plot assessed the potential publication bias of pooled effect in 28-day mortality for corticosteroids vs. placebo treatment in patients with sepsis. [file DataSheet_1.zip › Data Sheet 2/All supplemental figures/Supplemental Figure 1..tif]

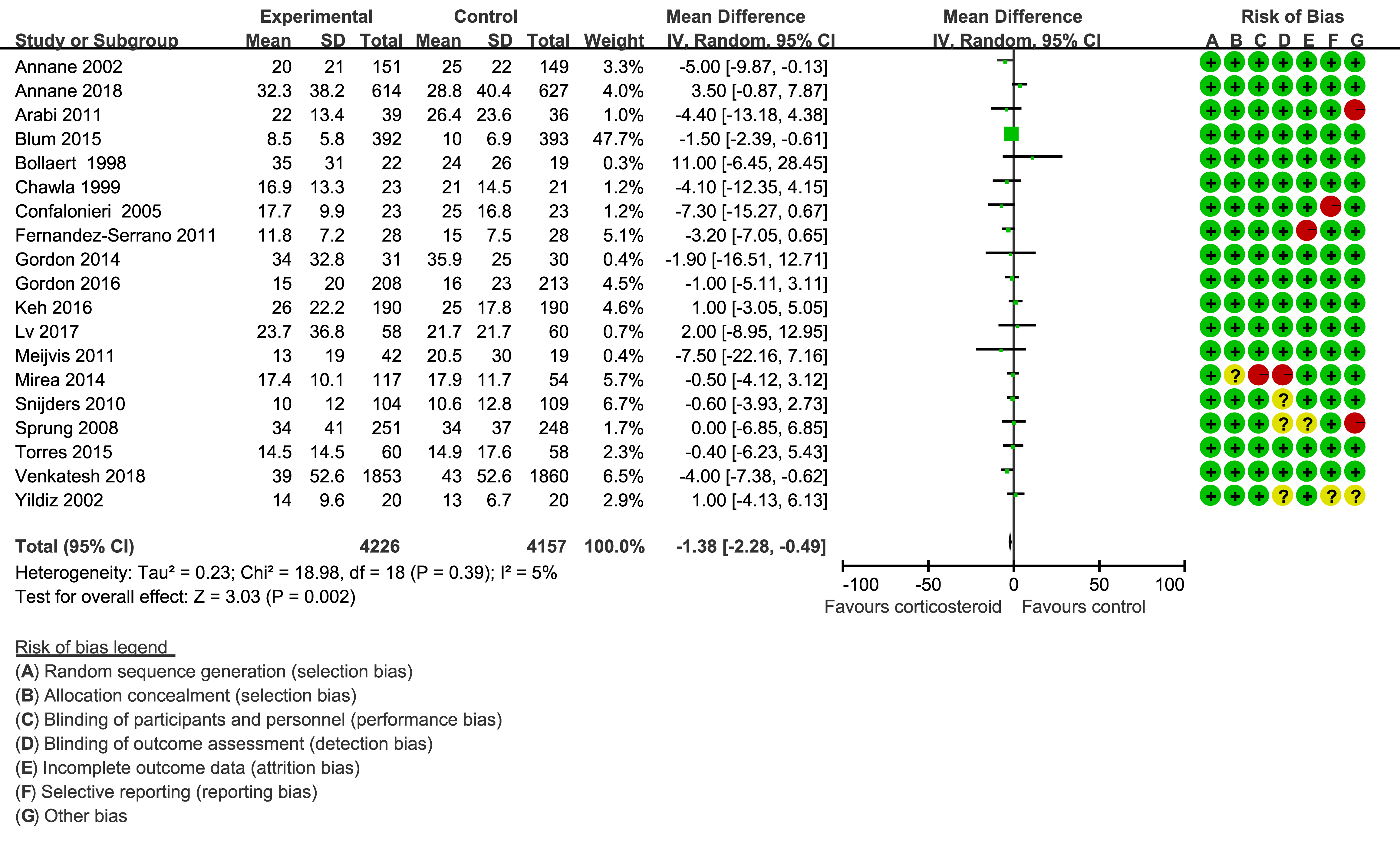

Supplement: Supplementary Figure 1 — The Funnel plot assessed the potential publication bias of pooled effect in 28-day mortality for corticosteroids vs. placebo treatment in patients with sepsis. [file DataSheet_1.zip › Data Sheet 2/All supplemental figures/Supplemental Figure 10.tif]

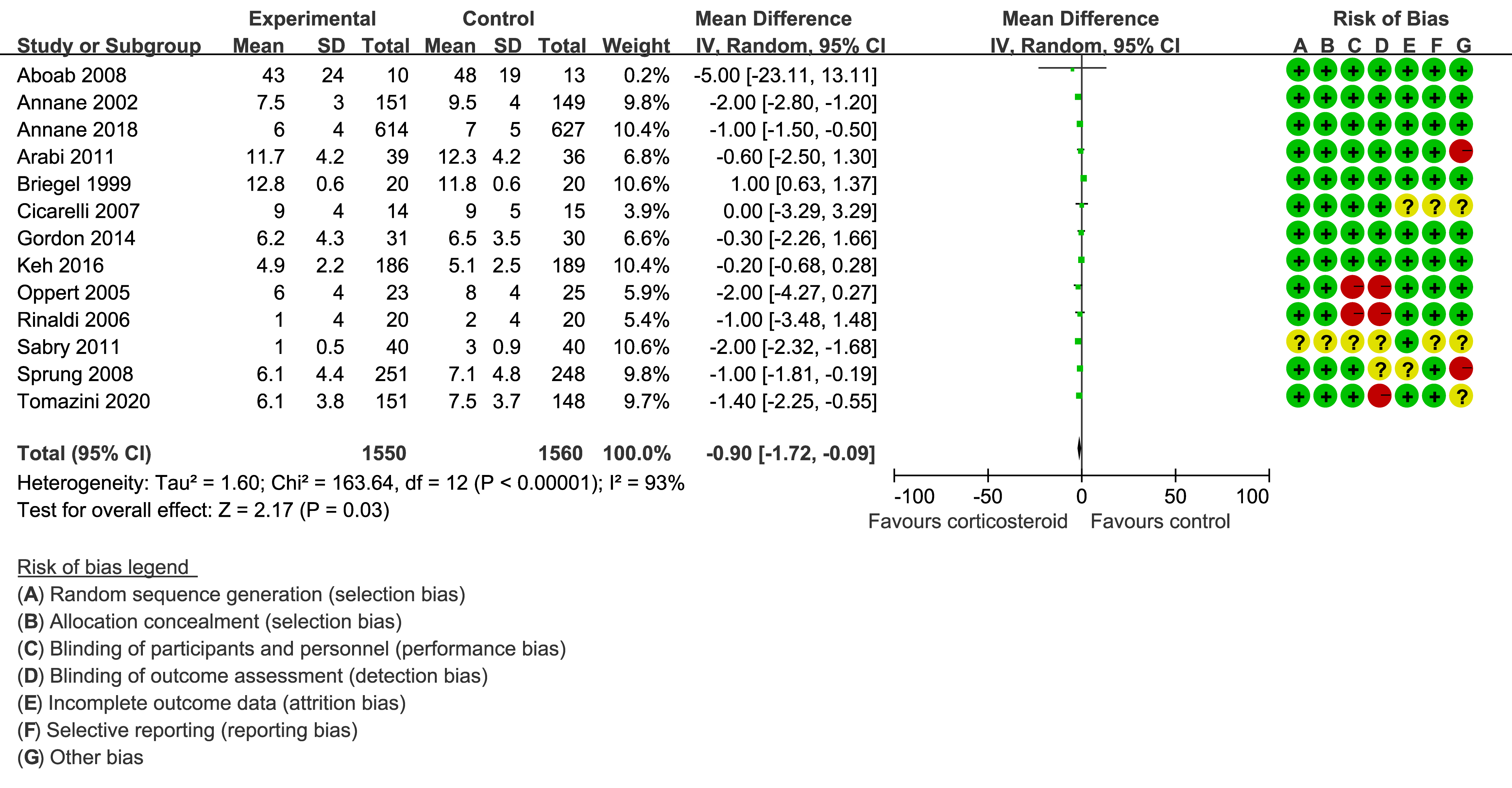

Supplement: Supplementary Figure 1 — The Funnel plot assessed the potential publication bias of pooled effect in 28-day mortality for corticosteroids vs. placebo treatment in patients with sepsis. [file DataSheet_1.zip › Data Sheet 2/All supplemental figures/Supplemental Figure 11.tif]

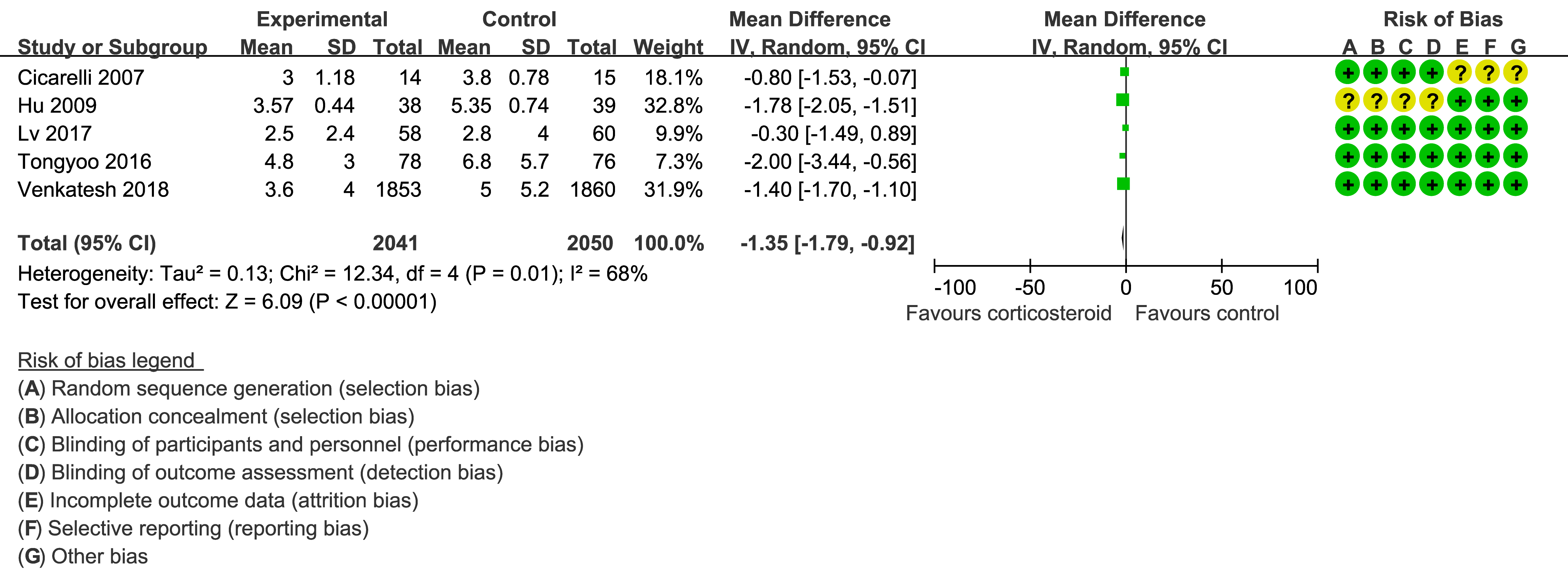

Supplement: Supplementary Figure 1 — The Funnel plot assessed the potential publication bias of pooled effect in 28-day mortality for corticosteroids vs. placebo treatment in patients with sepsis. [file DataSheet_1.zip › Data Sheet 2/All supplemental figures/Supplemental Figure 12.tif]

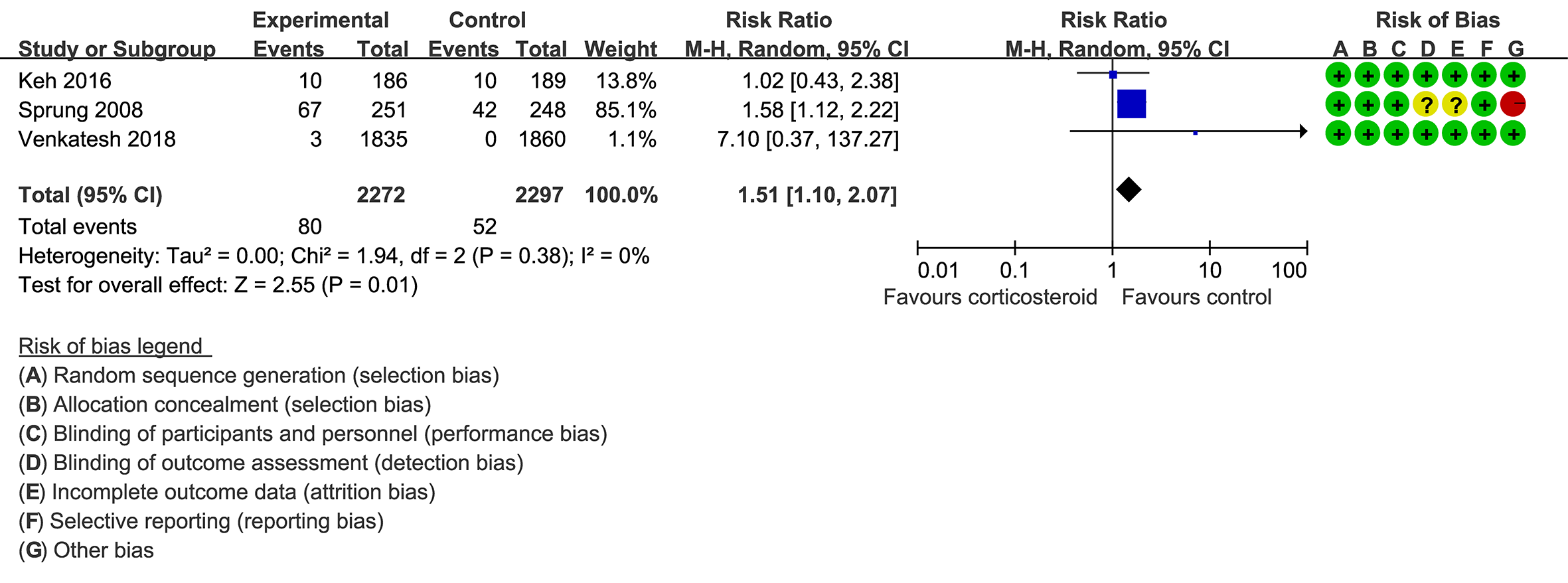

Supplement: Supplementary Figure 1 — The Funnel plot assessed the potential publication bias of pooled effect in 28-day mortality for corticosteroids vs. placebo treatment in patients with sepsis. [file DataSheet_1.zip › Data Sheet 2/All supplemental figures/Supplemental Figure 13.tif]

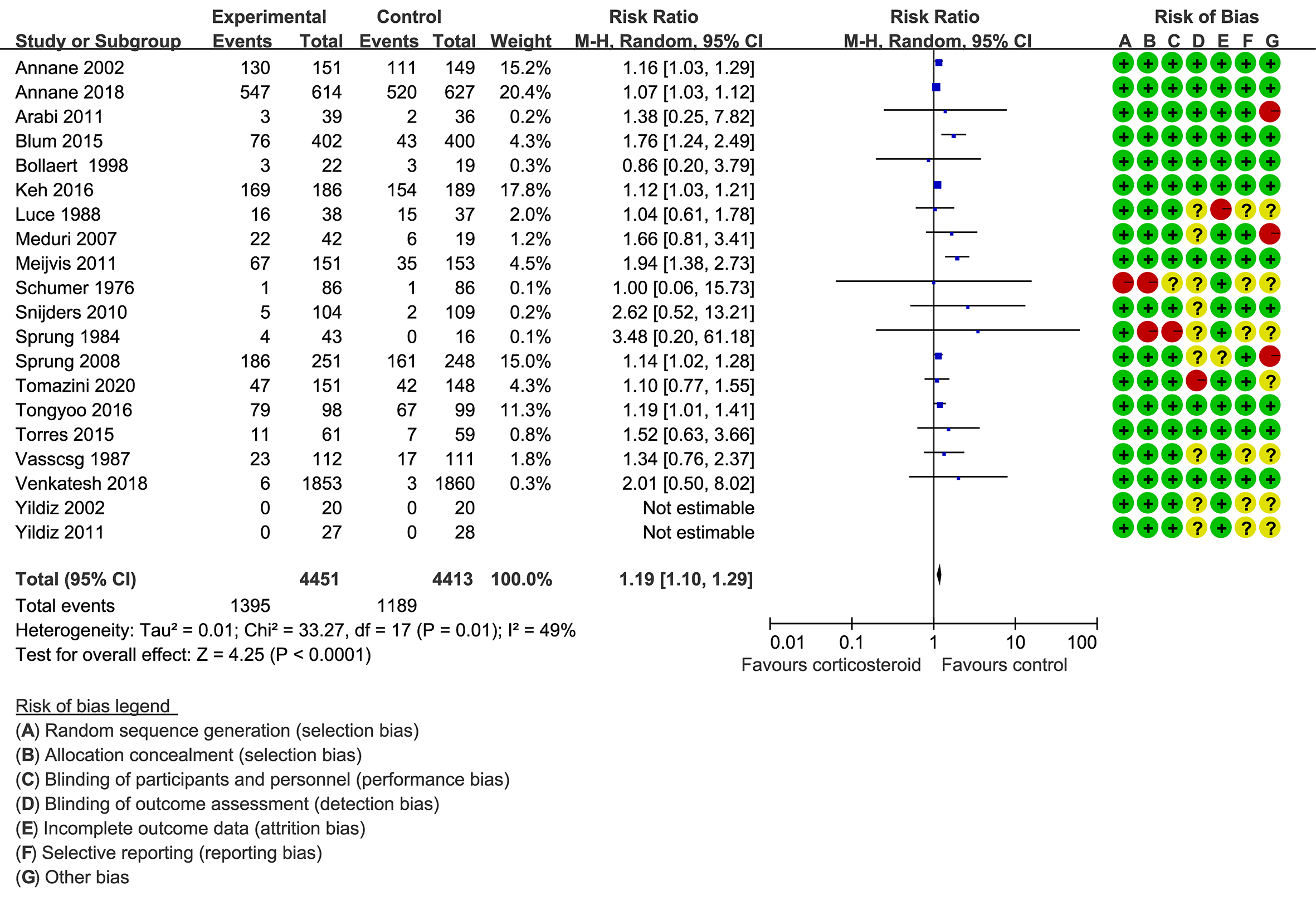

Supplement: Supplementary Figure 1 — The Funnel plot assessed the potential publication bias of pooled effect in 28-day mortality for corticosteroids vs. placebo treatment in patients with sepsis. [file DataSheet_1.zip › Data Sheet 2/All supplemental figures/Supplemental Figure 14.tif]

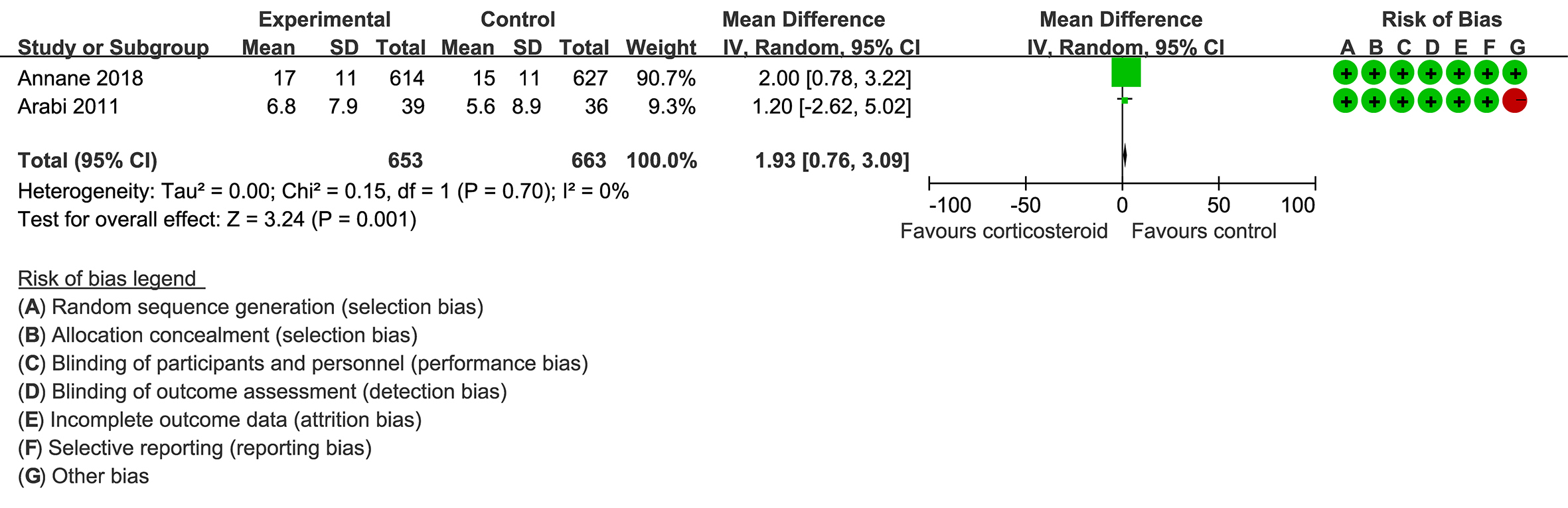

Supplement: Supplementary Figure 1 — The Funnel plot assessed the potential publication bias of pooled effect in 28-day mortality for corticosteroids vs. placebo treatment in patients with sepsis. [file DataSheet_1.zip › Data Sheet 2/All supplemental figures/Supplemental Figure 15.tif]

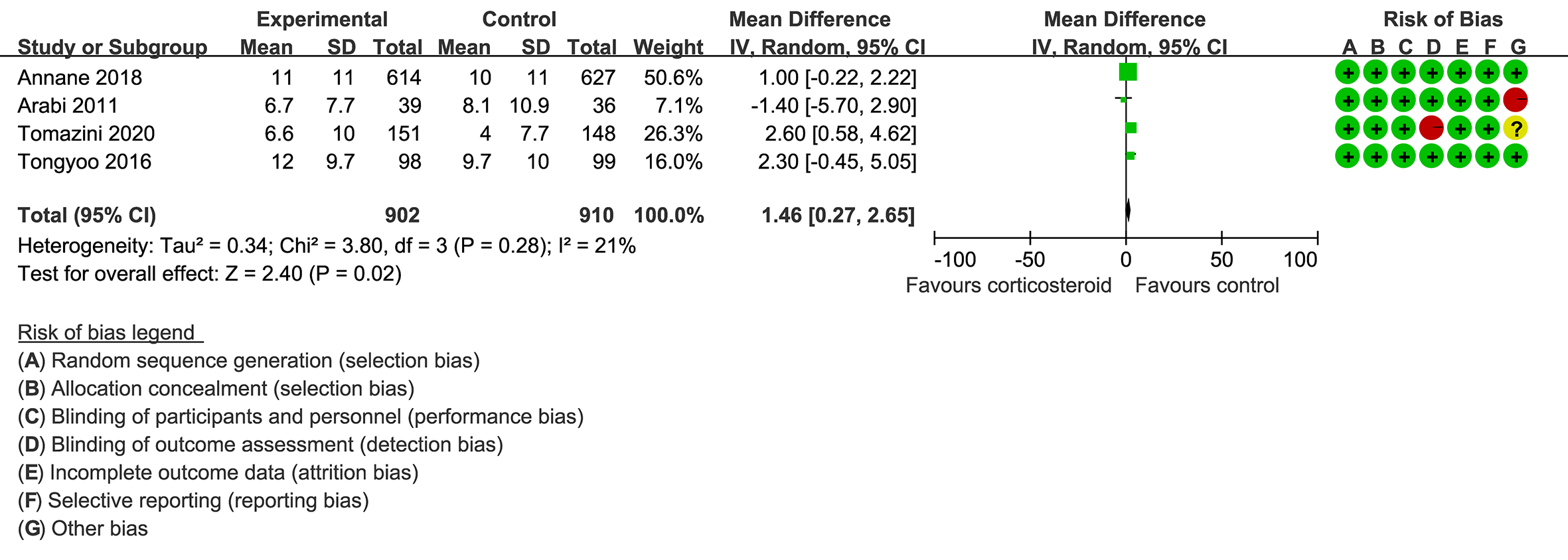

Supplement: Supplementary Figure 1 — The Funnel plot assessed the potential publication bias of pooled effect in 28-day mortality for corticosteroids vs. placebo treatment in patients with sepsis. [file DataSheet_1.zip › Data Sheet 2/All supplemental figures/Supplemental Figure 16.tif]

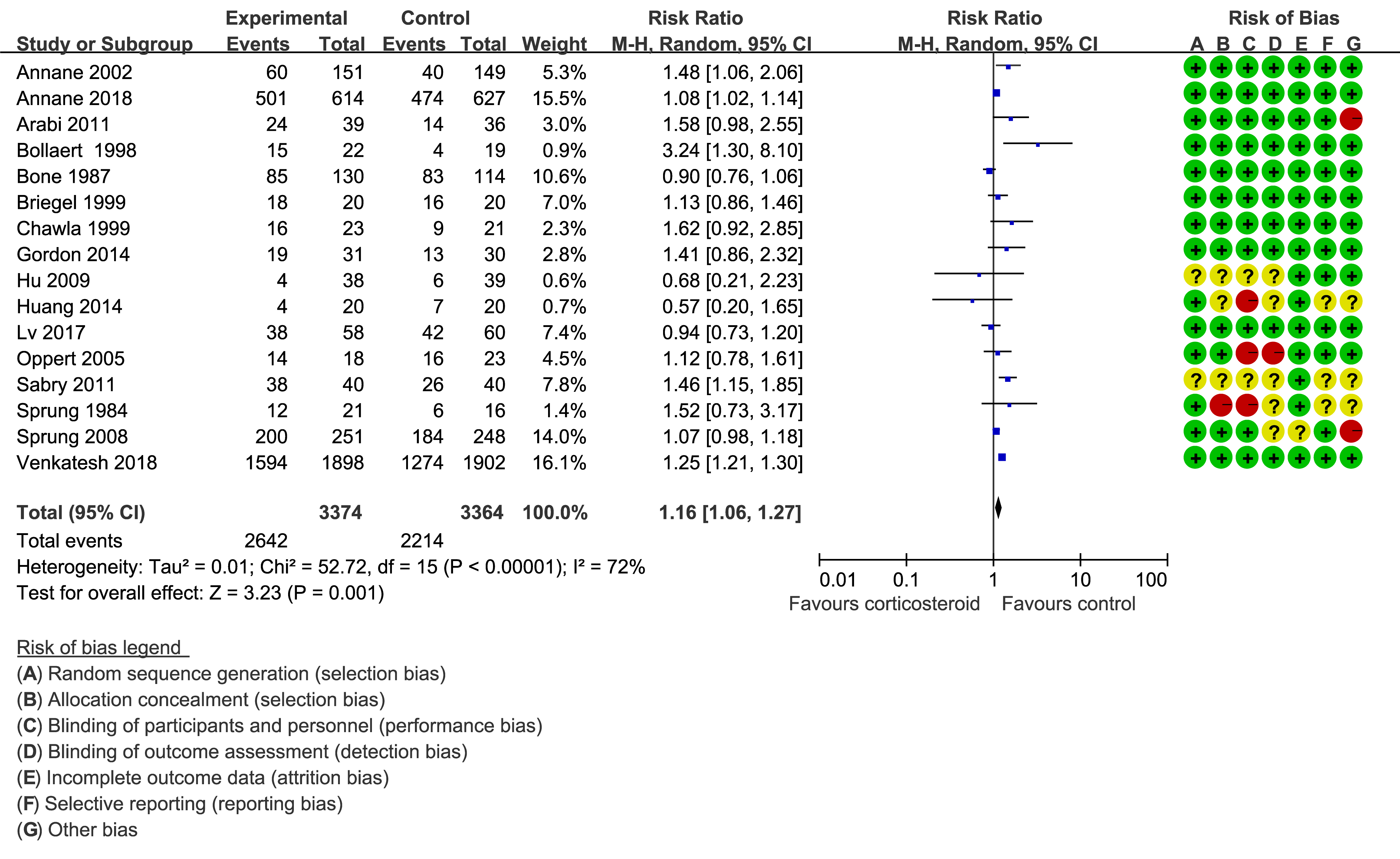

Supplement: Supplementary Figure 1 — The Funnel plot assessed the potential publication bias of pooled effect in 28-day mortality for corticosteroids vs. placebo treatment in patients with sepsis. [file DataSheet_1.zip › Data Sheet 2/All supplemental figures/Supplemental Figure 17.tif]

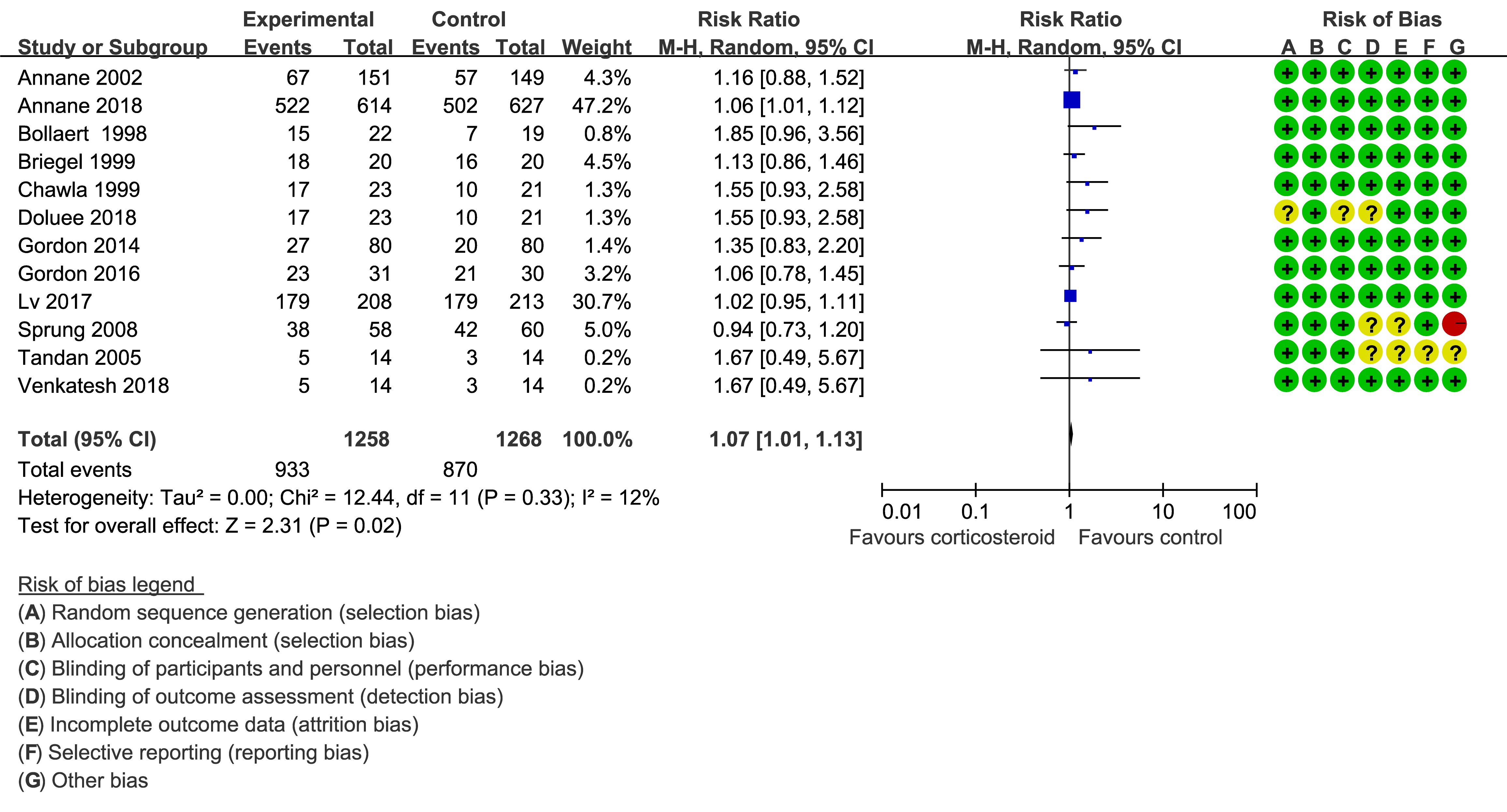

Supplement: Supplementary Figure 1 — The Funnel plot assessed the potential publication bias of pooled effect in 28-day mortality for corticosteroids vs. placebo treatment in patients with sepsis. [file DataSheet_1.zip › Data Sheet 2/All supplemental figures/Supplemental Figure 18.tif]

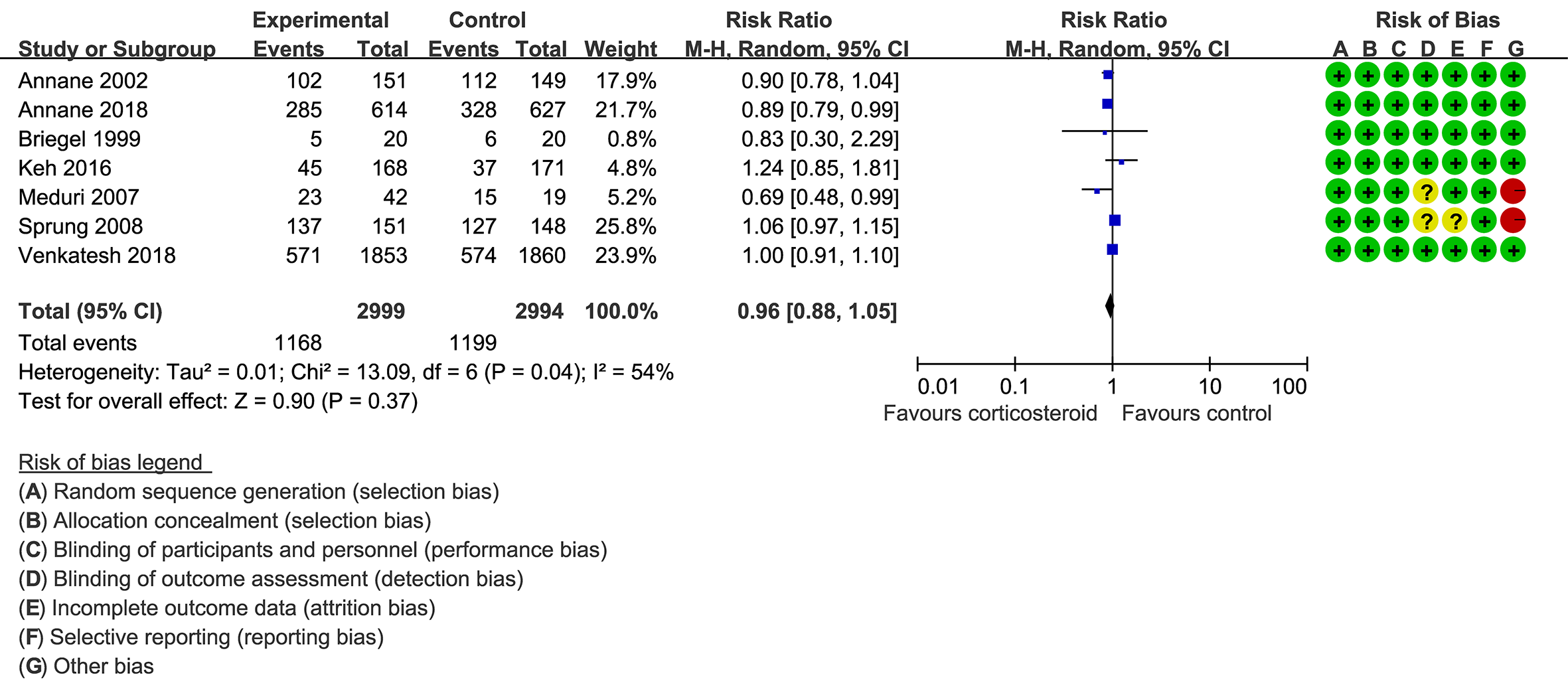

Supplement: Supplementary Figure 1 — The Funnel plot assessed the potential publication bias of pooled effect in 28-day mortality for corticosteroids vs. placebo treatment in patients with sepsis. [file DataSheet_1.zip › Data Sheet 2/All supplemental figures/Supplemental Figure 19.tif]

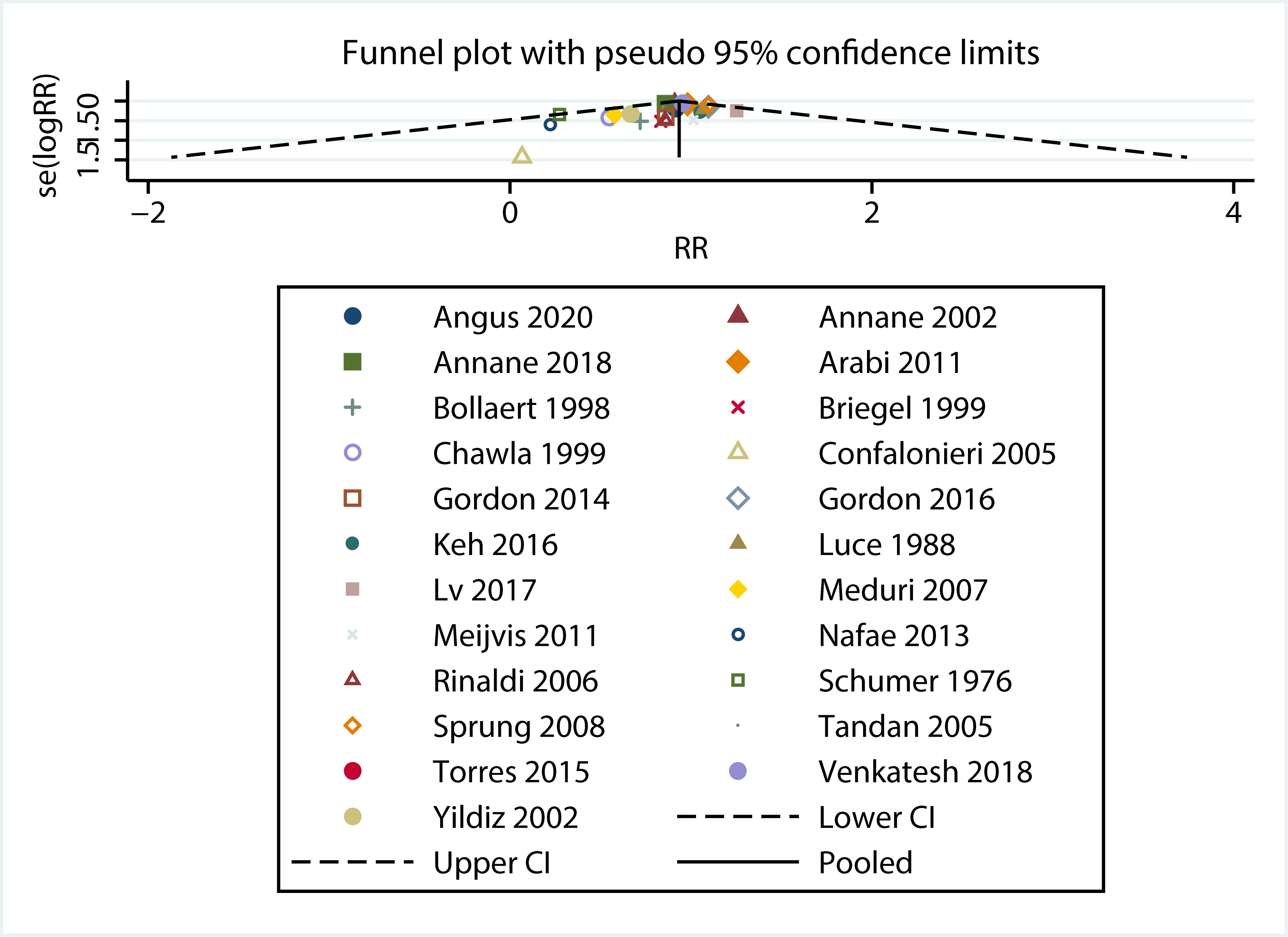

Supplement: Supplementary Figure 1 — The Funnel plot assessed the potential publication bias of pooled effect in 28-day mortality for corticosteroids vs. placebo treatment in patients with sepsis. [file DataSheet_1.zip › Data Sheet 2/All supplemental figures/Supplemental Figure 2.tif]

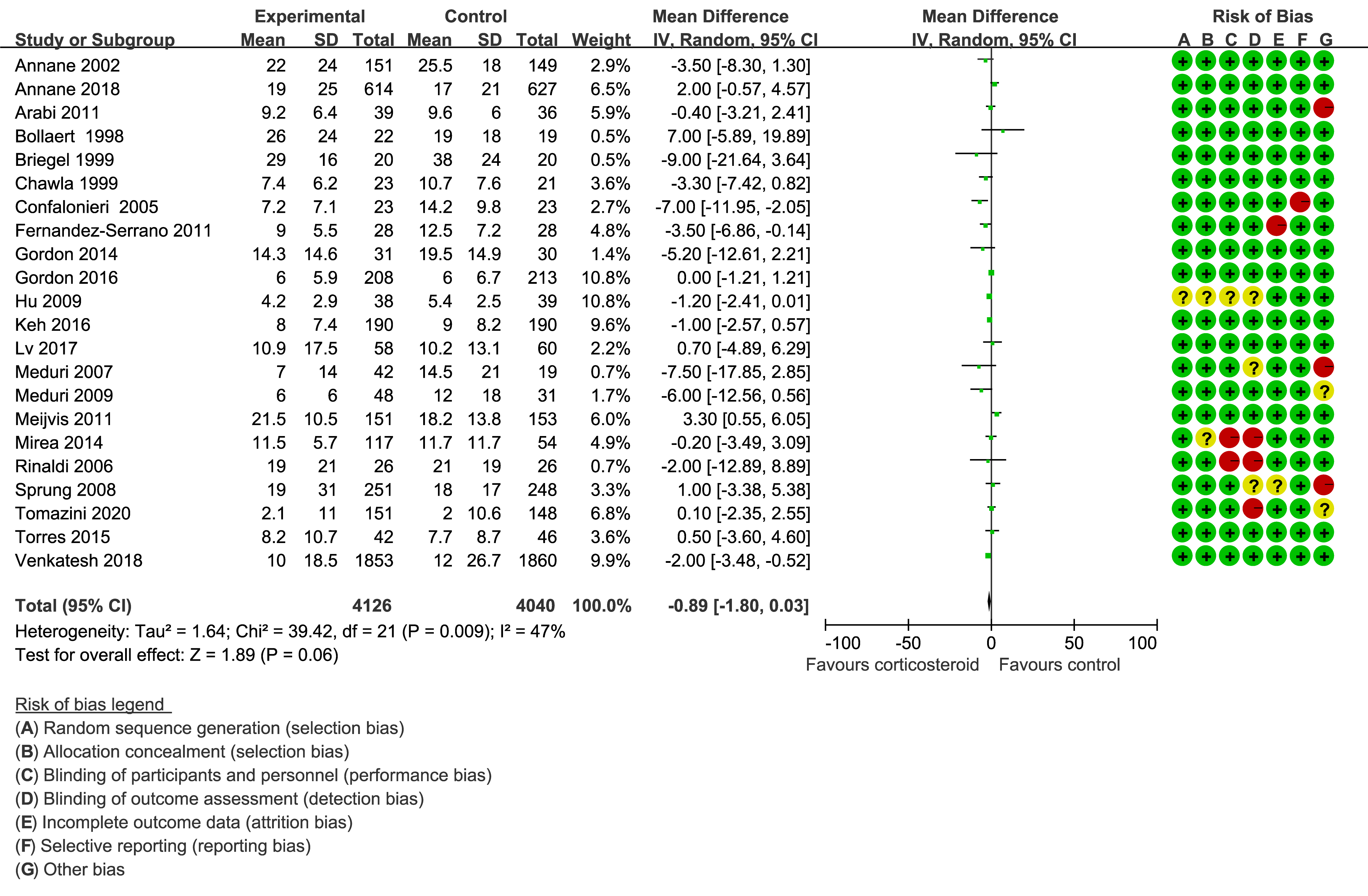

Supplement: Supplementary Figure 1 — The Funnel plot assessed the potential publication bias of pooled effect in 28-day mortality for corticosteroids vs. placebo treatment in patients with sepsis. [file DataSheet_1.zip › Data Sheet 2/All supplemental figures/Supplemental Figure 20.tif]

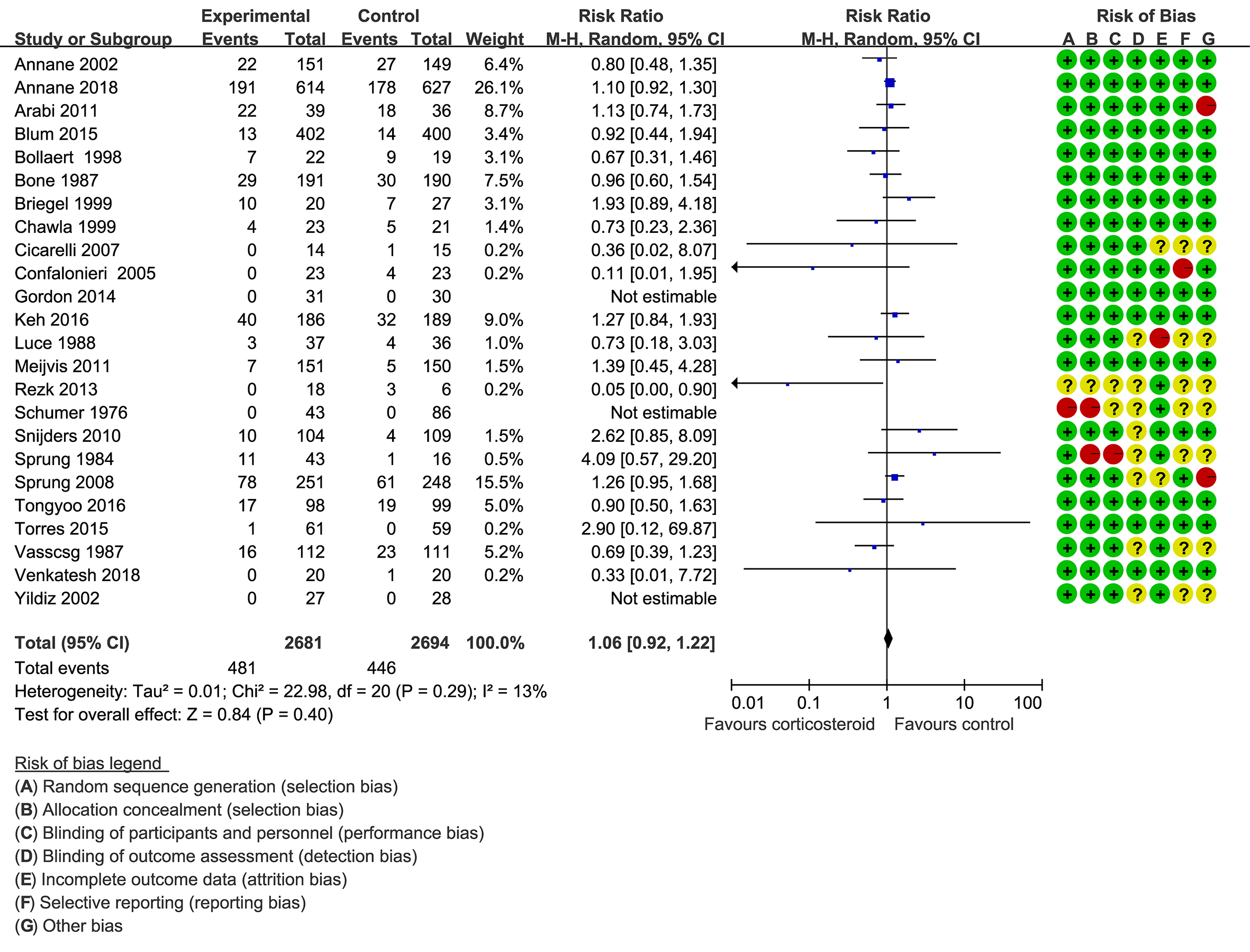

Supplement: Supplementary Figure 1 — The Funnel plot assessed the potential publication bias of pooled effect in 28-day mortality for corticosteroids vs. placebo treatment in patients with sepsis. [file DataSheet_1.zip › Data Sheet 2/All supplemental figures/Supplemental Figure 21.tif]

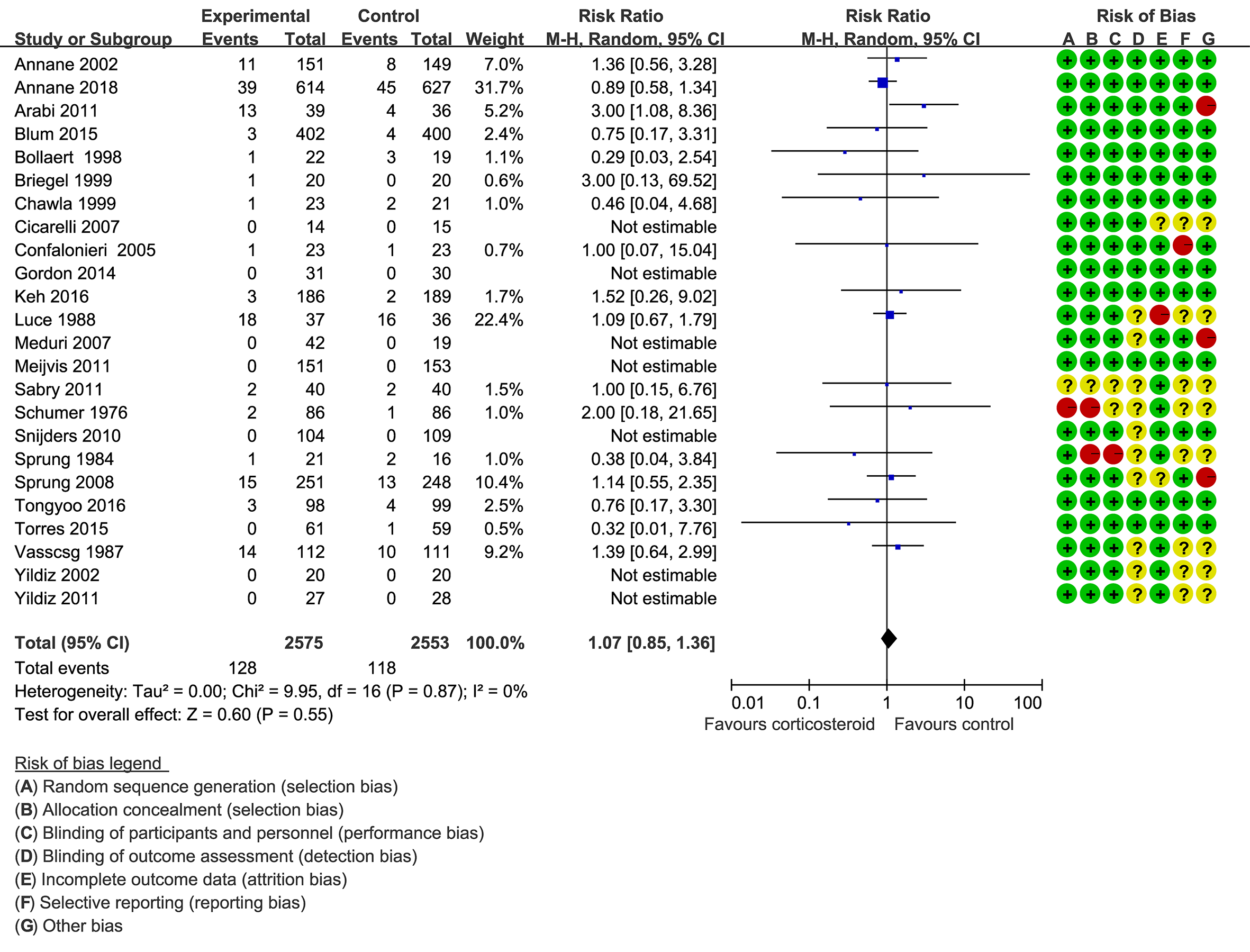

Supplement: Supplementary Figure 1 — The Funnel plot assessed the potential publication bias of pooled effect in 28-day mortality for corticosteroids vs. placebo treatment in patients with sepsis. [file DataSheet_1.zip › Data Sheet 2/All supplemental figures/Supplemental Figure 22.tif]

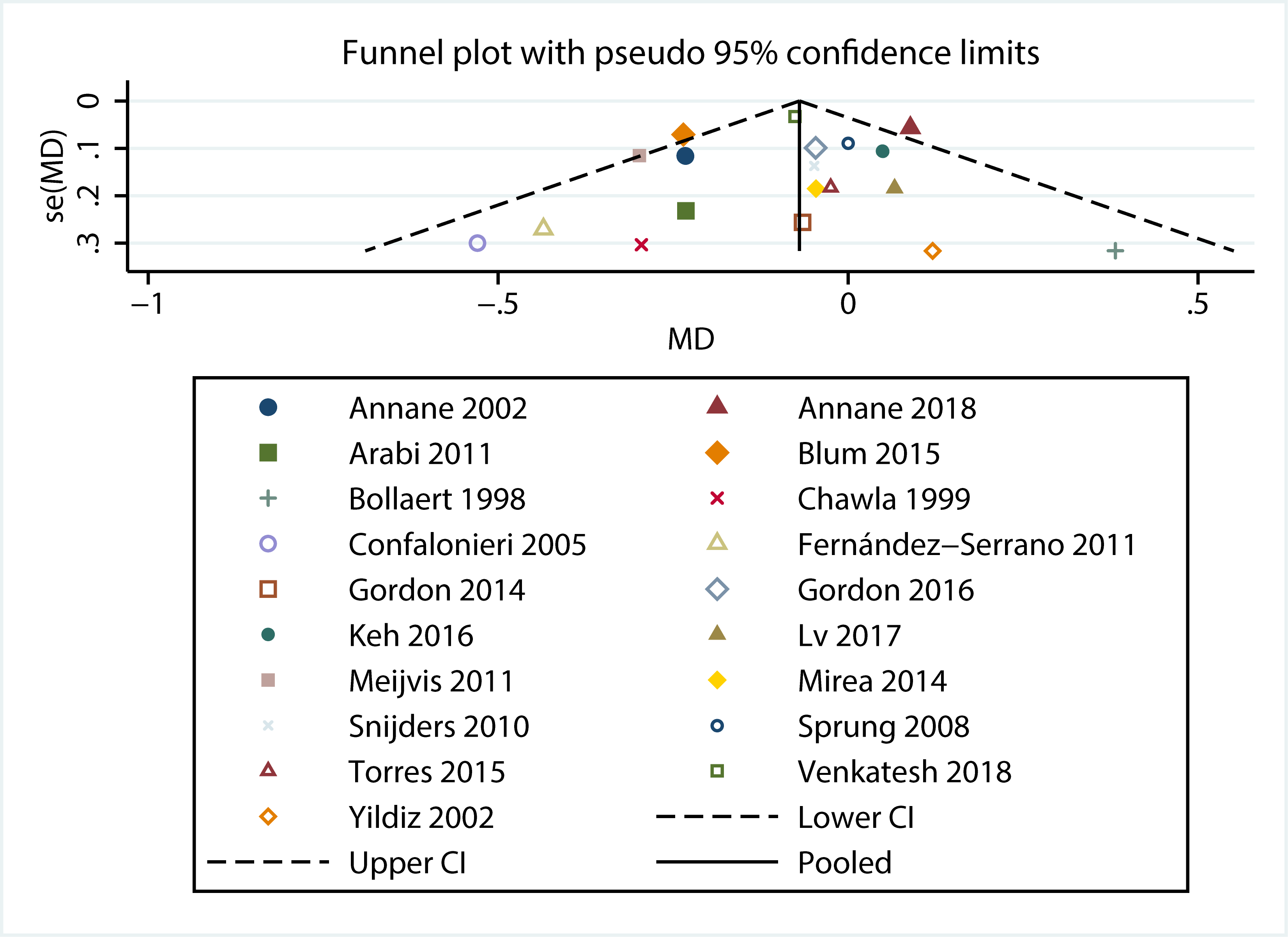

Supplement: Supplementary Figure 1 — The Funnel plot assessed the potential publication bias of pooled effect in 28-day mortality for corticosteroids vs. placebo treatment in patients with sepsis. [file DataSheet_1.zip › Data Sheet 2/All supplemental figures/Supplemental Figure 23.tif]

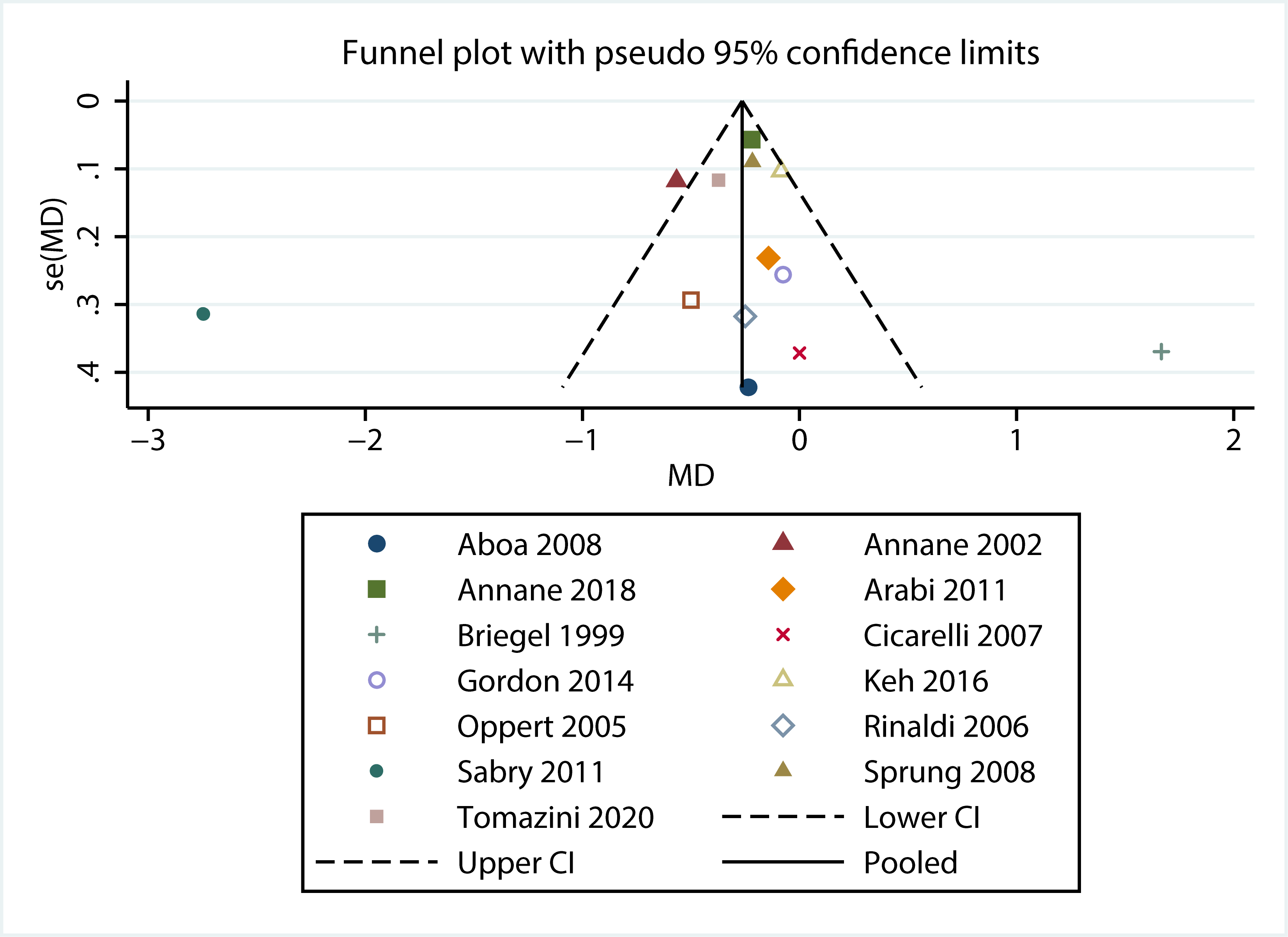

Supplement: Supplementary Figure 1 — The Funnel plot assessed the potential publication bias of pooled effect in 28-day mortality for corticosteroids vs. placebo treatment in patients with sepsis. [file DataSheet_1.zip › Data Sheet 2/All supplemental figures/Supplemental Figure 24.tif]

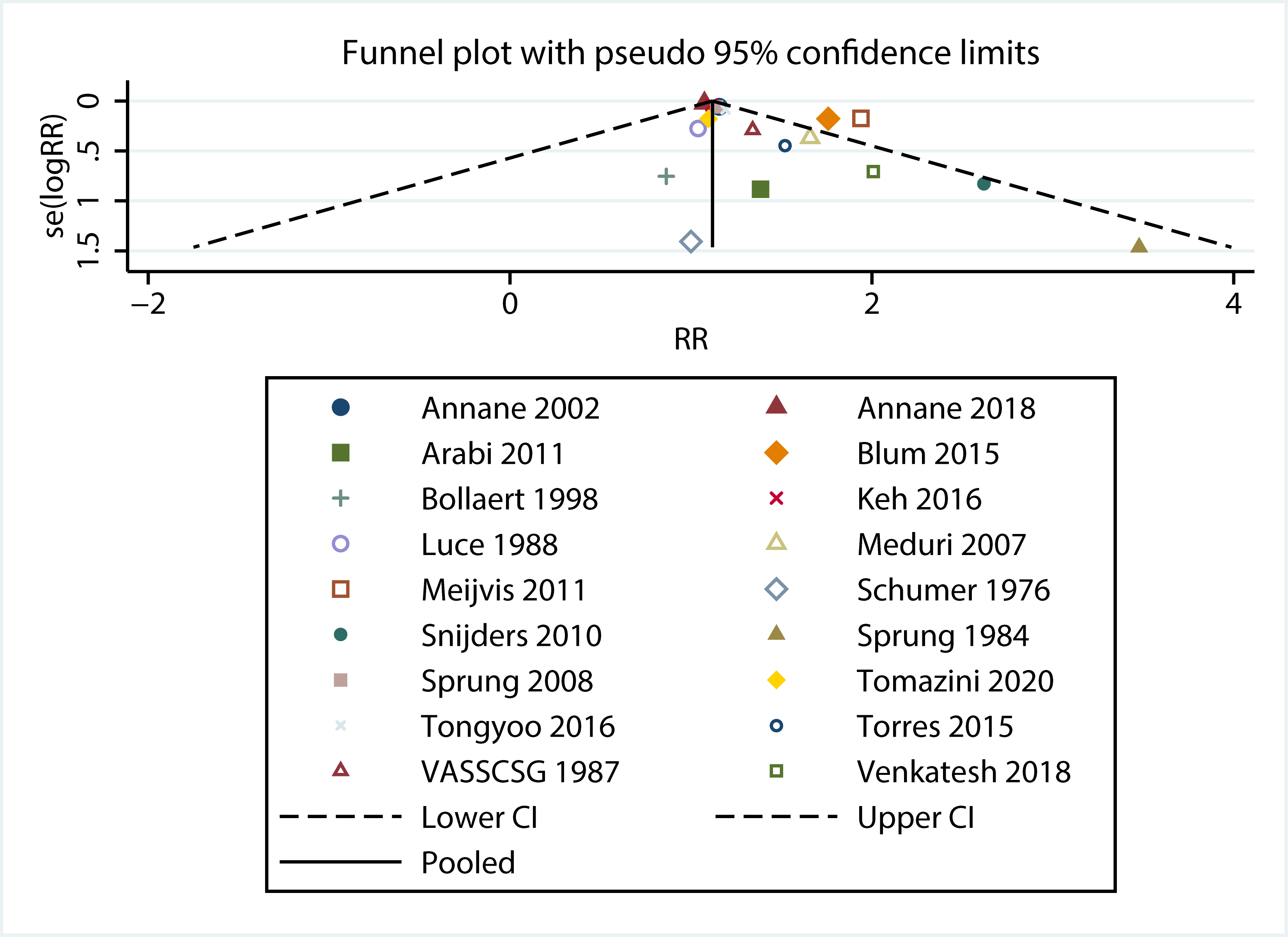

Supplement: Supplementary Figure 1 — The Funnel plot assessed the potential publication bias of pooled effect in 28-day mortality for corticosteroids vs. placebo treatment in patients with sepsis. [file DataSheet_1.zip › Data Sheet 2/All supplemental figures/Supplemental Figure 25.tif]

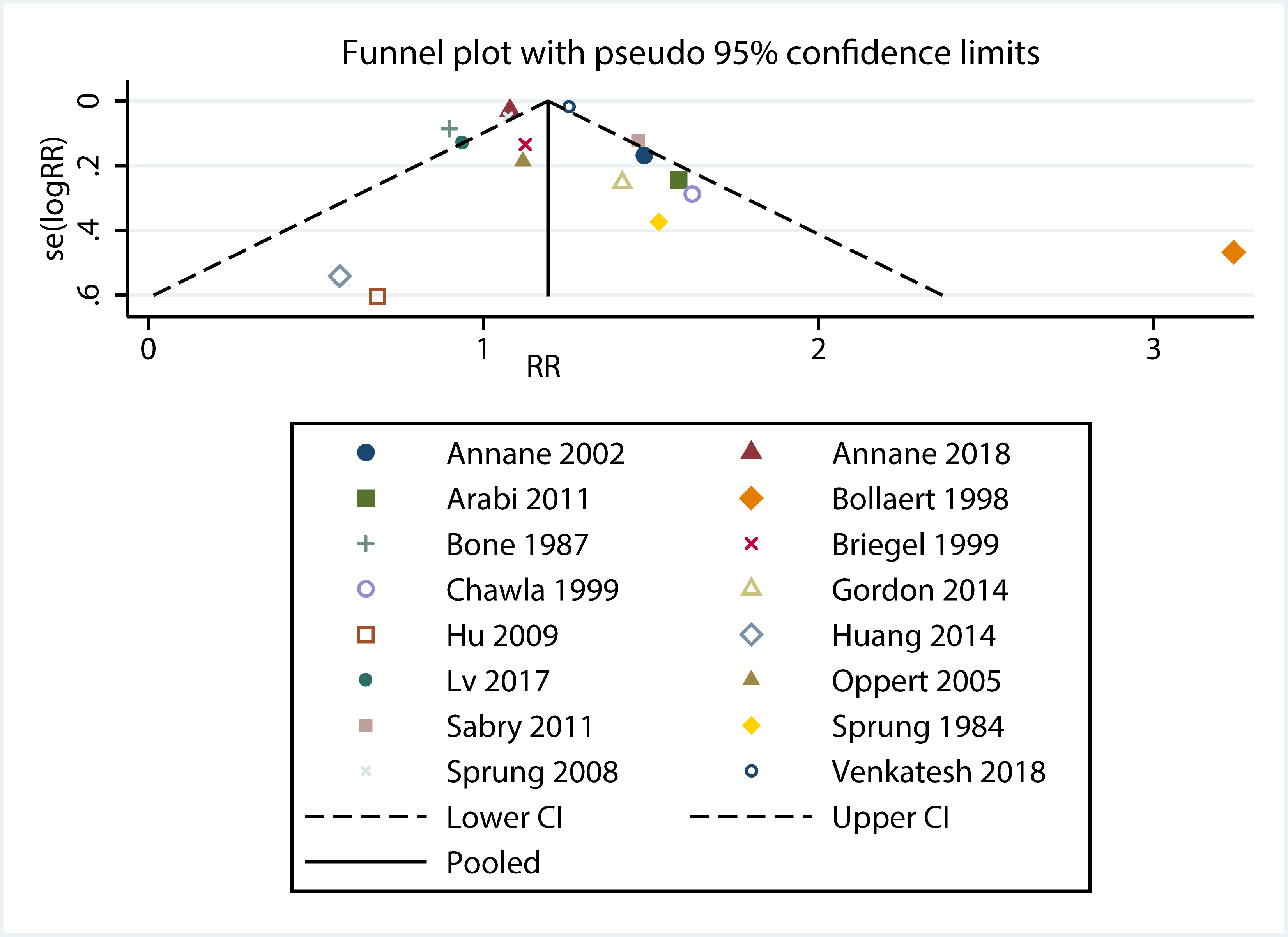

Supplement: Supplementary Figure 1 — The Funnel plot assessed the potential publication bias of pooled effect in 28-day mortality for corticosteroids vs. placebo treatment in patients with sepsis. [file DataSheet_1.zip › Data Sheet 2/All supplemental figures/Supplemental Figure 26.tif]

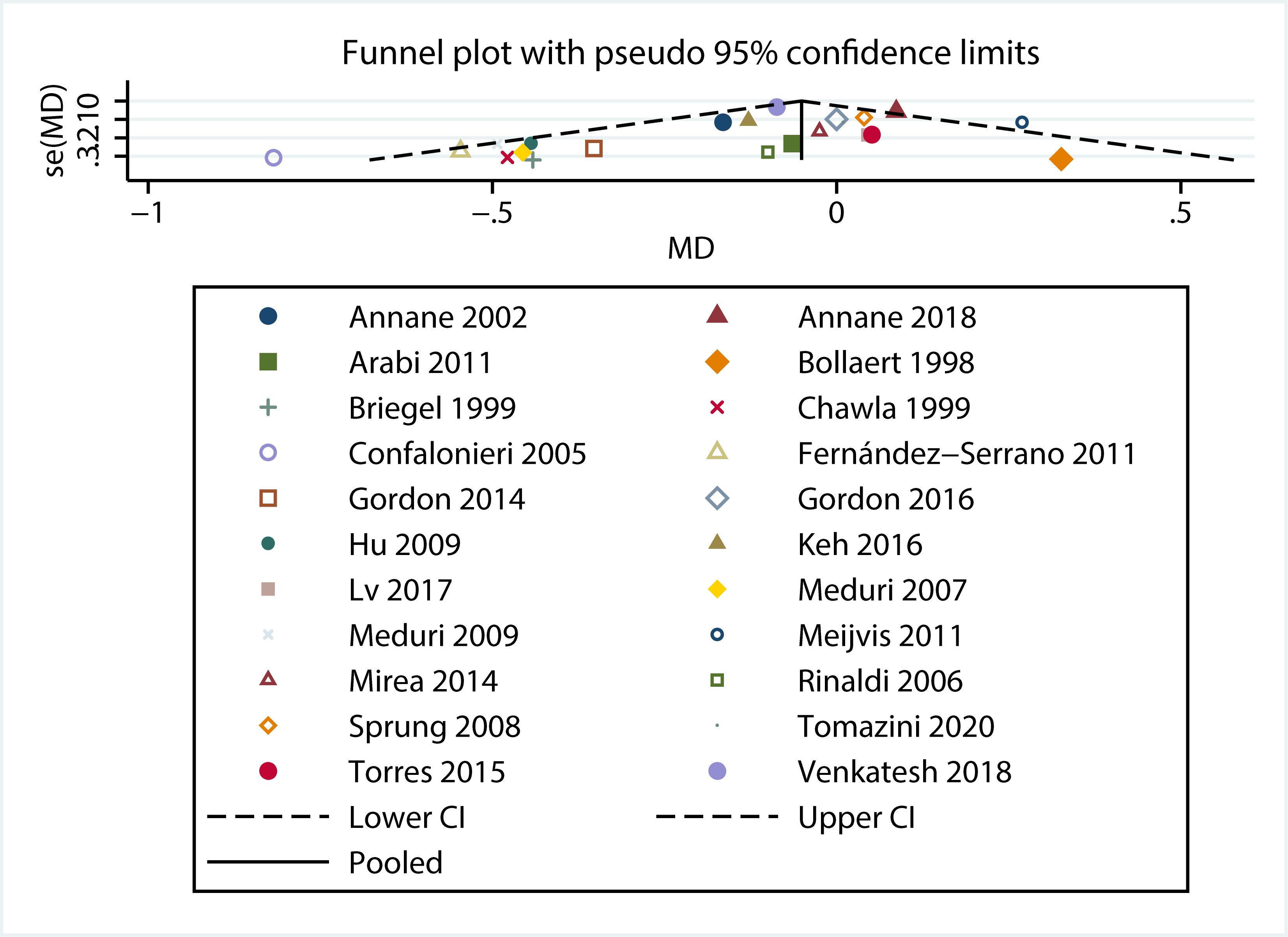

Supplement: Supplementary Figure 1 — The Funnel plot assessed the potential publication bias of pooled effect in 28-day mortality for corticosteroids vs. placebo treatment in patients with sepsis. [file DataSheet_1.zip › Data Sheet 2/All supplemental figures/Supplemental Figure 27.tif]

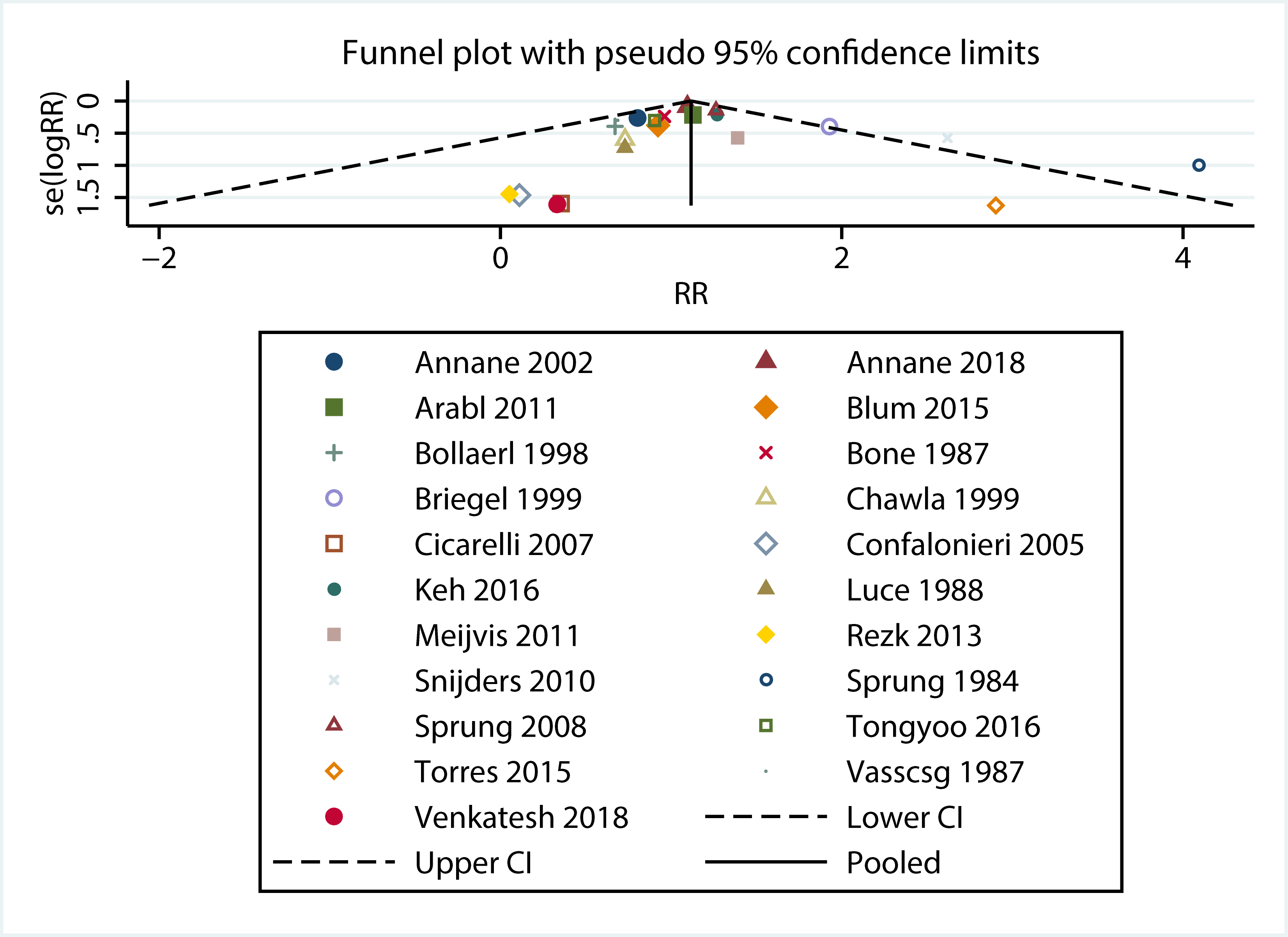

Supplement: Supplementary Figure 1 — The Funnel plot assessed the potential publication bias of pooled effect in 28-day mortality for corticosteroids vs. placebo treatment in patients with sepsis. [file DataSheet_1.zip › Data Sheet 2/All supplemental figures/Supplemental Figure 28.tif]

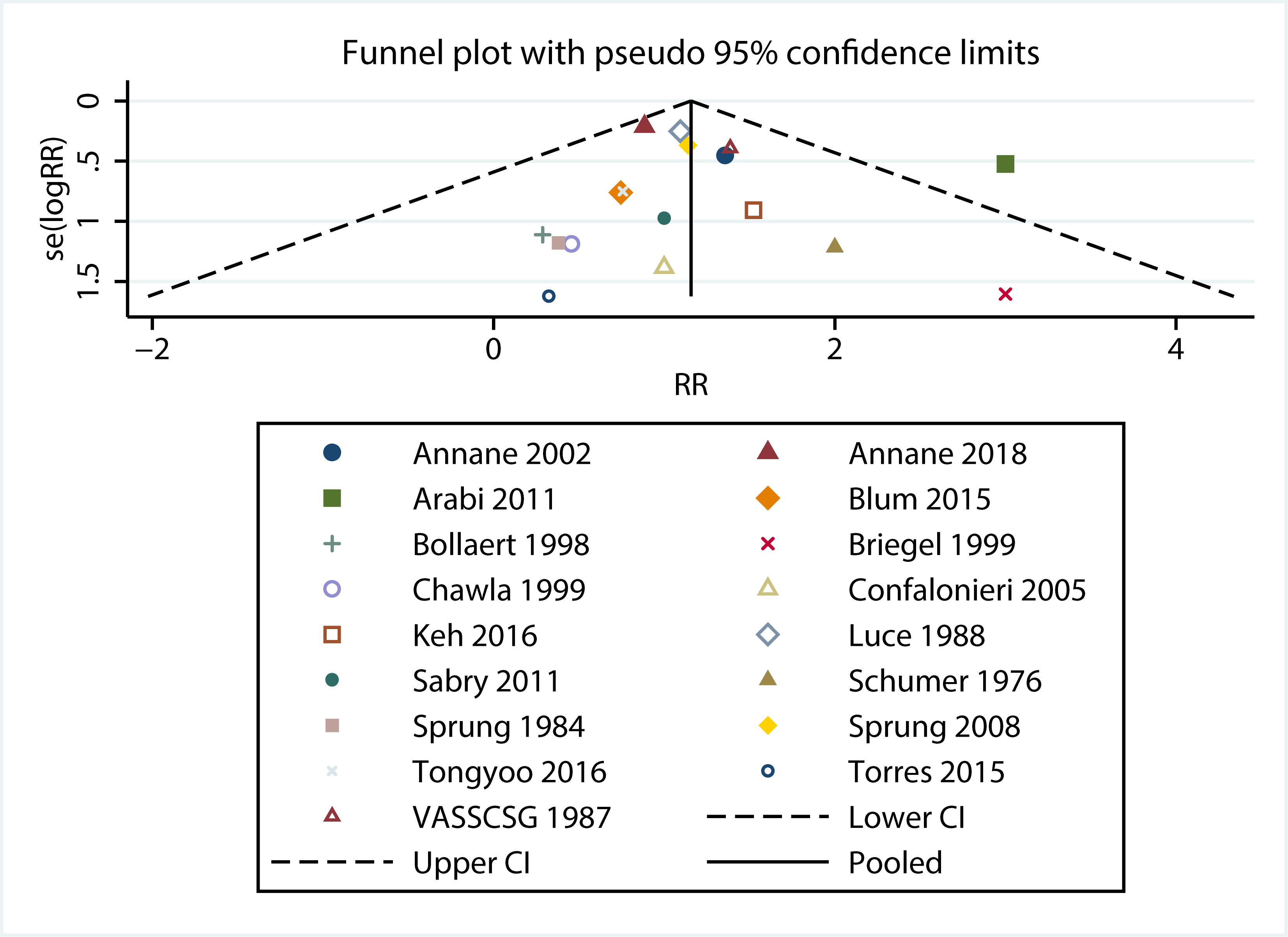

Supplement: Supplementary Figure 1 — The Funnel plot assessed the potential publication bias of pooled effect in 28-day mortality for corticosteroids vs. placebo treatment in patients with sepsis. [file DataSheet_1.zip › Data Sheet 2/All supplemental figures/Supplemental Figure 29.tif]

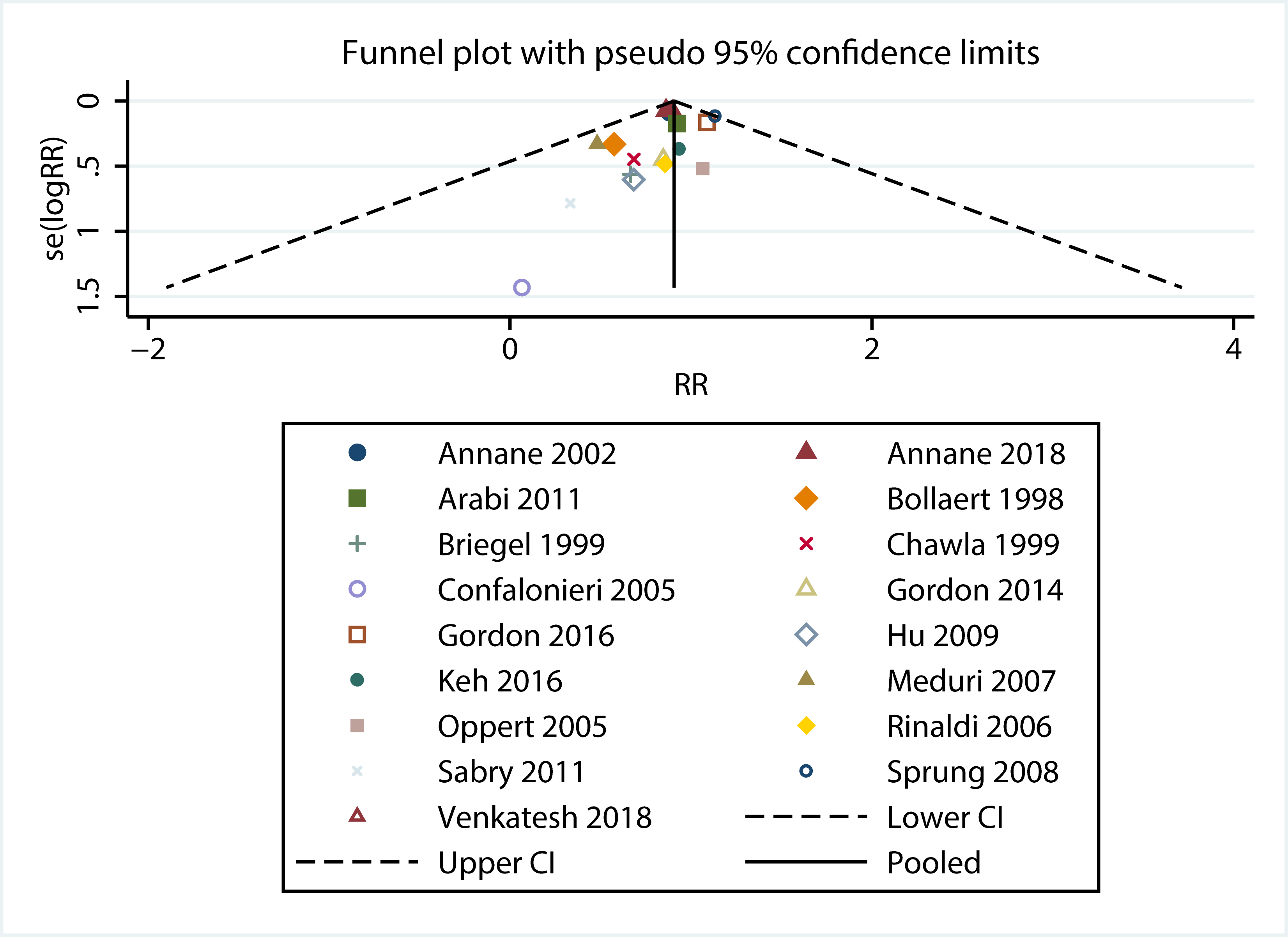

Supplement: Supplementary Figure 1 — The Funnel plot assessed the potential publication bias of pooled effect in 28-day mortality for corticosteroids vs. placebo treatment in patients with sepsis. [file DataSheet_1.zip › Data Sheet 2/All supplemental figures/Supplemental Figure 3.tif]

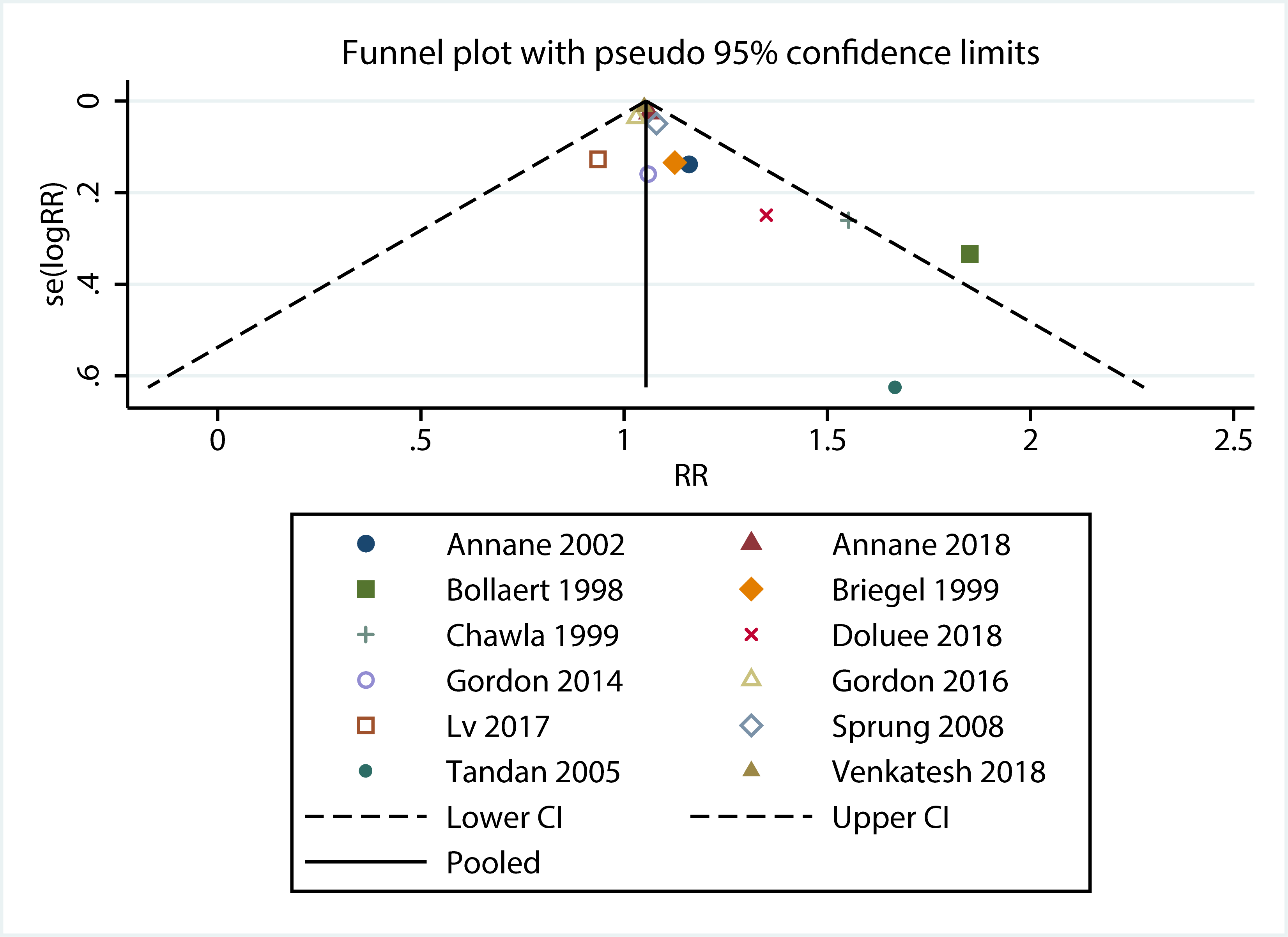

Supplement: Supplementary Figure 1 — The Funnel plot assessed the potential publication bias of pooled effect in 28-day mortality for corticosteroids vs. placebo treatment in patients with sepsis. [file DataSheet_1.zip › Data Sheet 2/All supplemental figures/Supplemental Figure 30.tif]

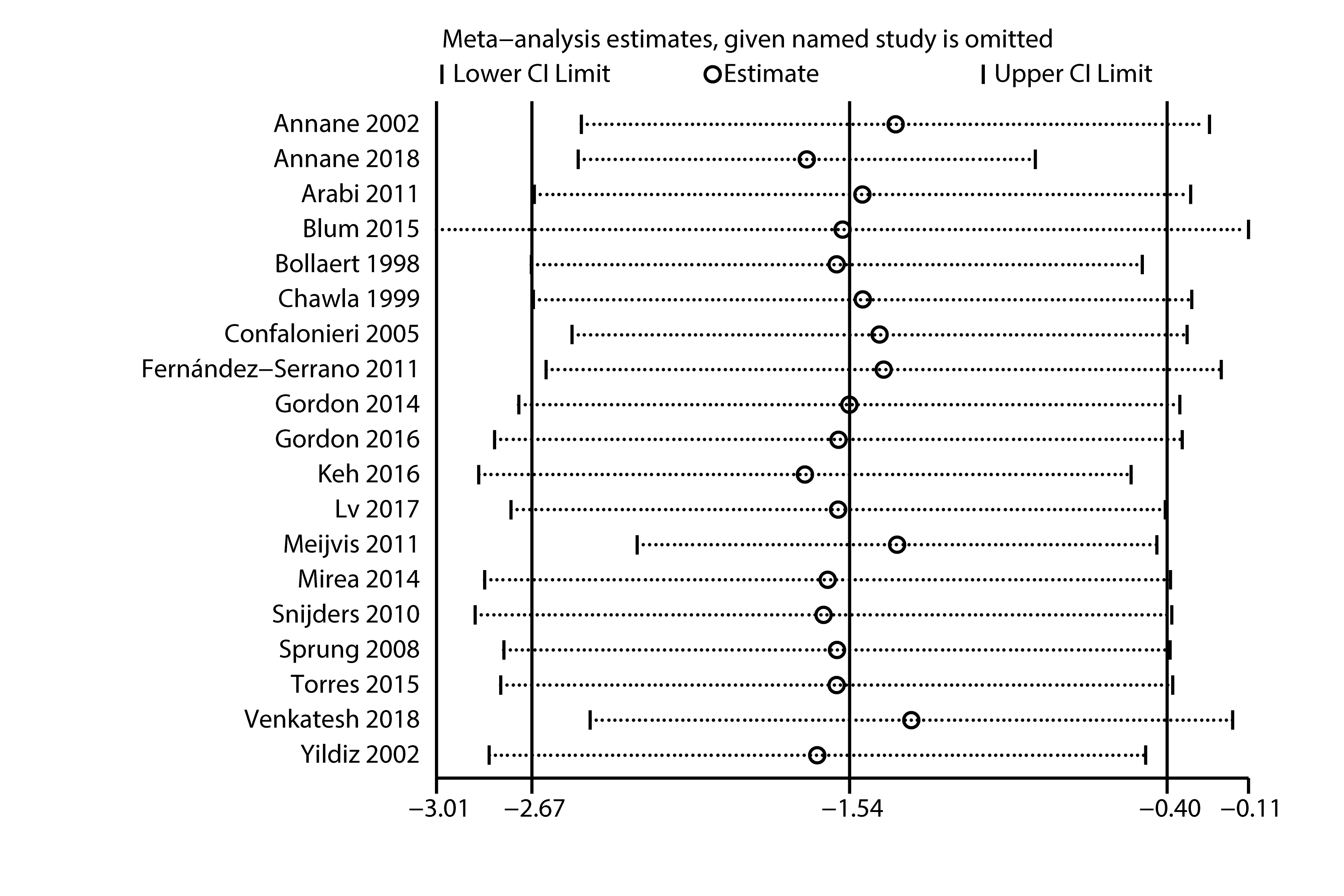

Supplement: Supplementary Figure 1 — The Funnel plot assessed the potential publication bias of pooled effect in 28-day mortality for corticosteroids vs. placebo treatment in patients with sepsis. [file DataSheet_1.zip › Data Sheet 2/All supplemental figures/Supplemental Figure 31.tif]

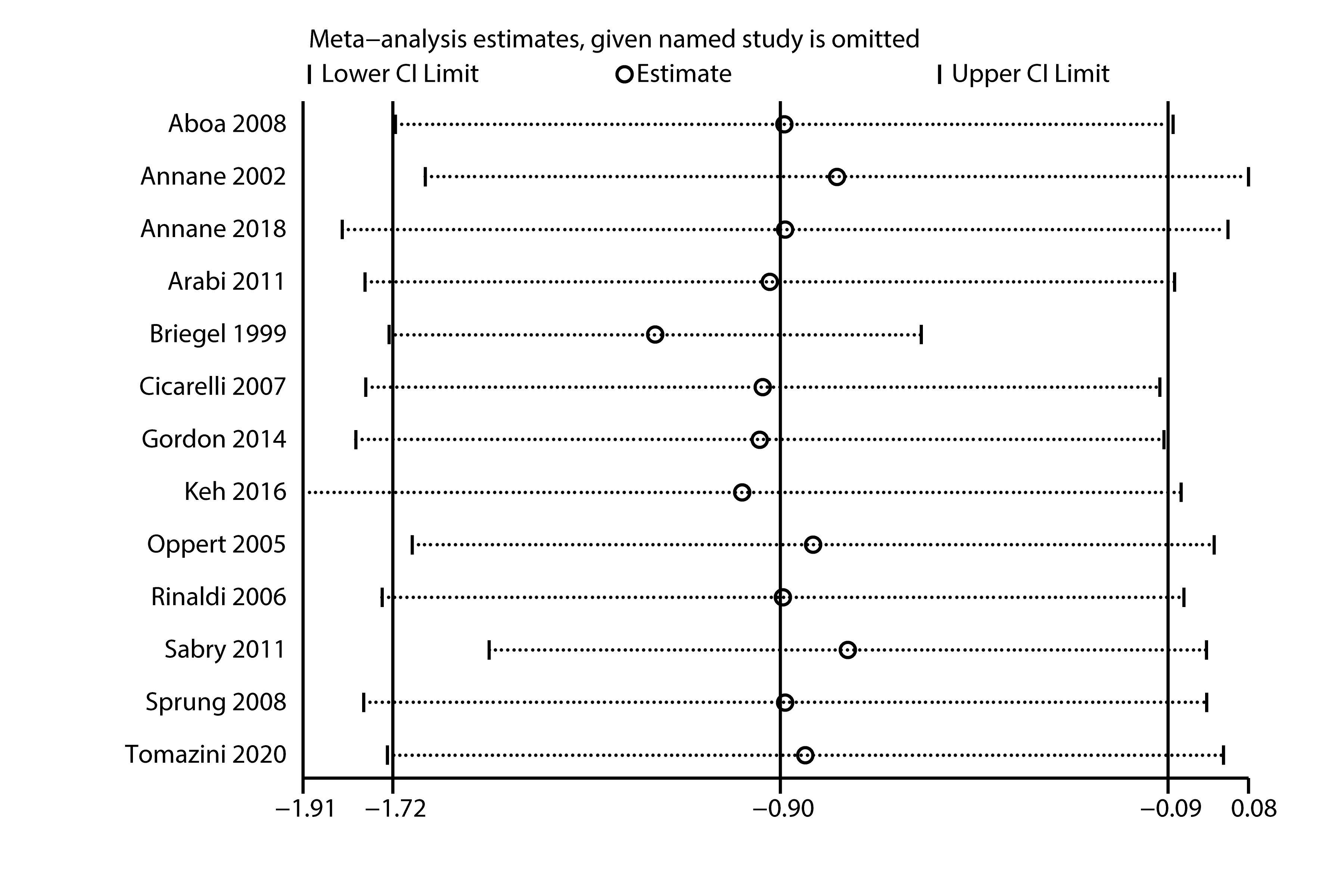

Supplement: Supplementary Figure 1 — The Funnel plot assessed the potential publication bias of pooled effect in 28-day mortality for corticosteroids vs. placebo treatment in patients with sepsis. [file DataSheet_1.zip › Data Sheet 2/All supplemental figures/Supplemental Figure 32.tif]

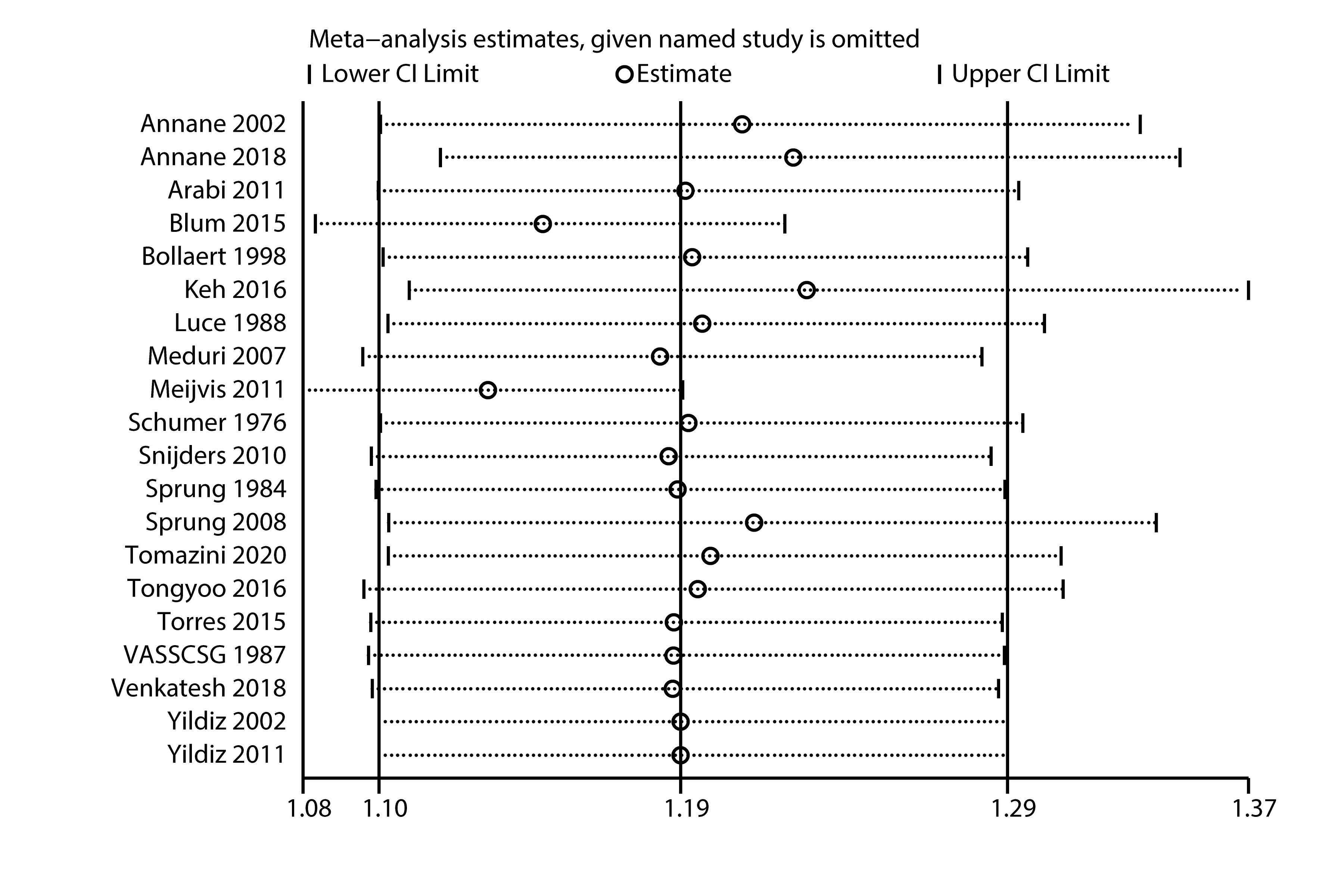

Supplement: Supplementary Figure 1 — The Funnel plot assessed the potential publication bias of pooled effect in 28-day mortality for corticosteroids vs. placebo treatment in patients with sepsis. [file DataSheet_1.zip › Data Sheet 2/All supplemental figures/Supplemental Figure 33.tif]

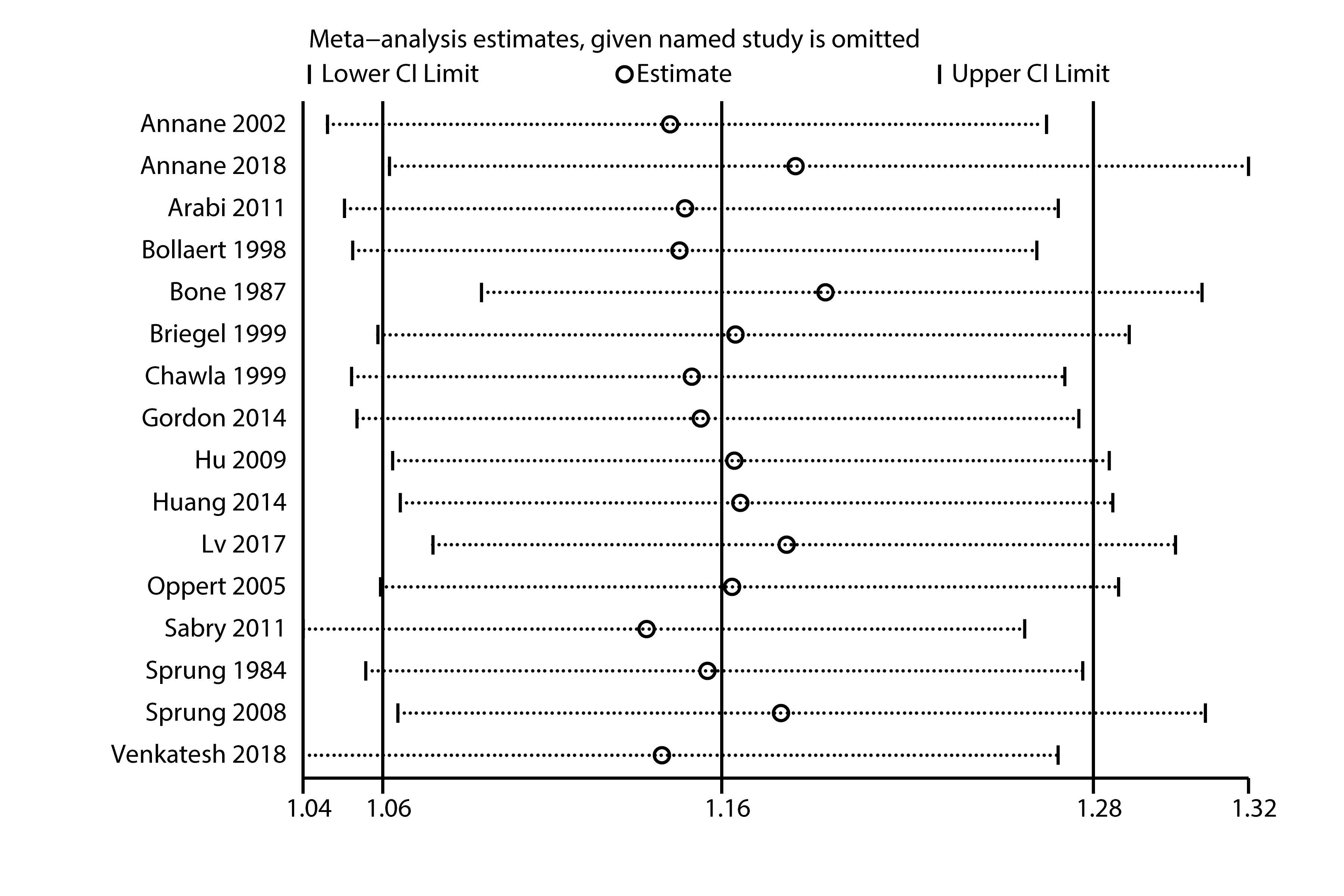

Supplement: Supplementary Figure 1 — The Funnel plot assessed the potential publication bias of pooled effect in 28-day mortality for corticosteroids vs. placebo treatment in patients with sepsis. [file DataSheet_1.zip › Data Sheet 2/All supplemental figures/Supplemental Figure 34.tif]

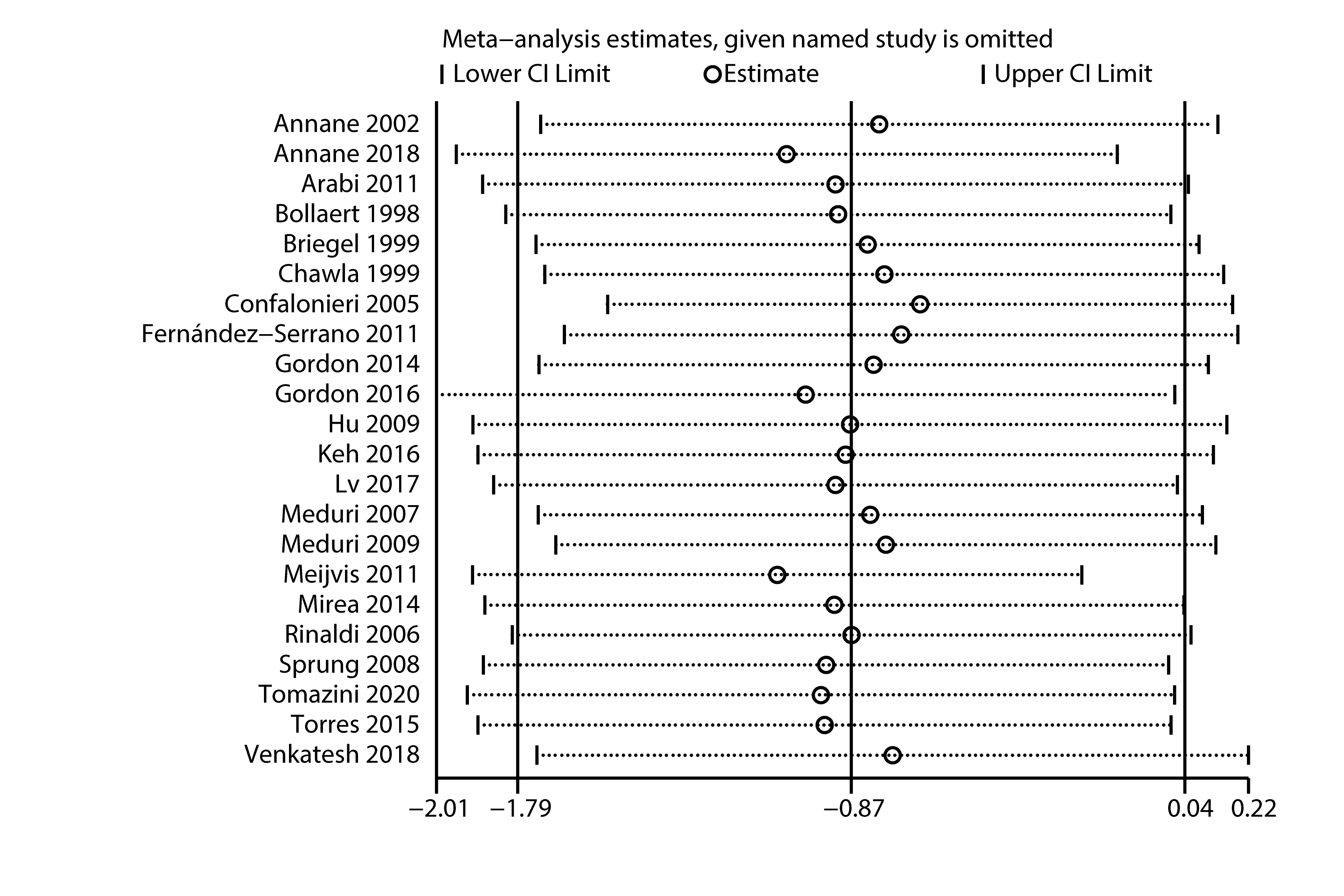

Supplement: Supplementary Figure 1 — The Funnel plot assessed the potential publication bias of pooled effect in 28-day mortality for corticosteroids vs. placebo treatment in patients with sepsis. [file DataSheet_1.zip › Data Sheet 2/All supplemental figures/Supplemental Figure 35.tif]

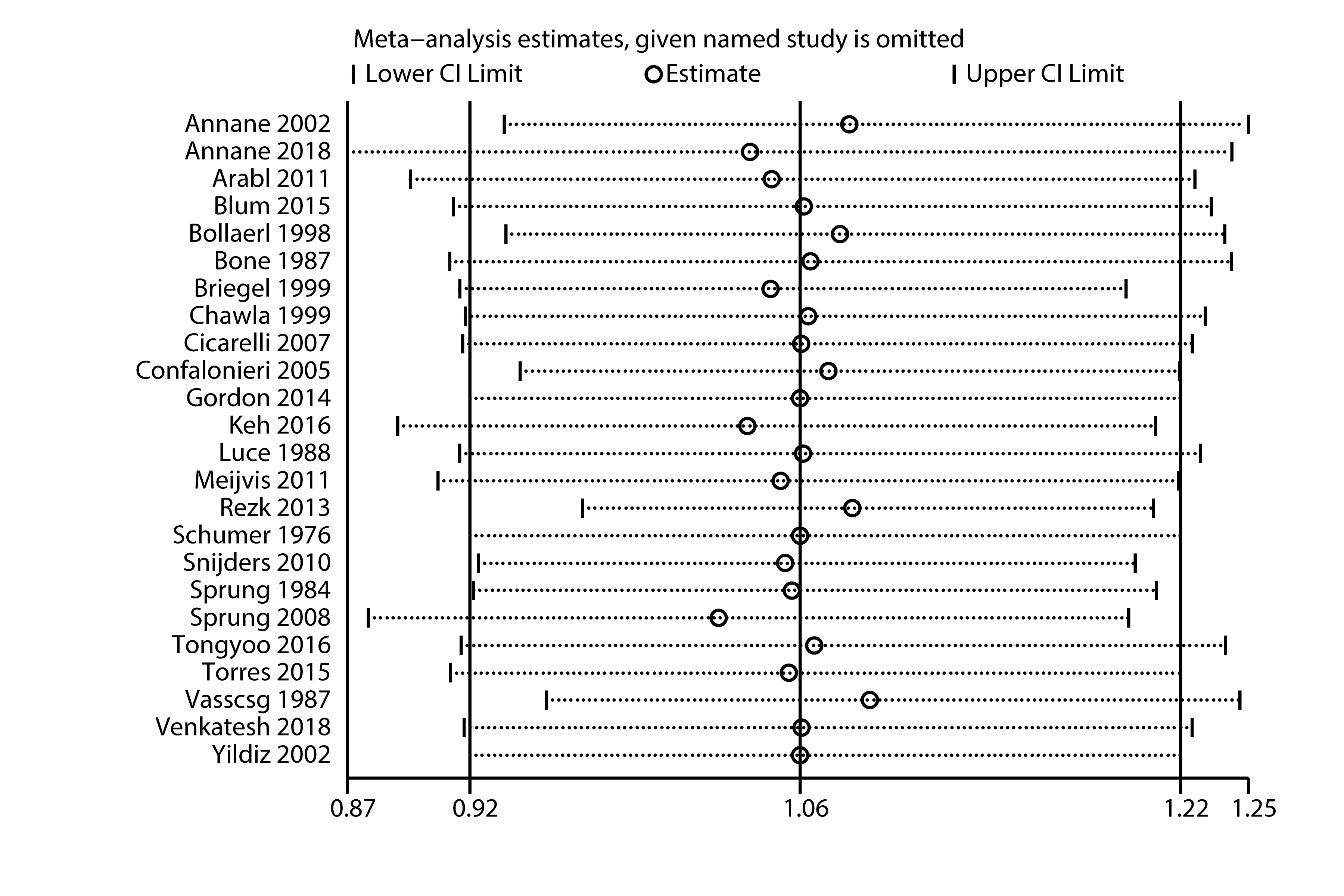

Supplement: Supplementary Figure 1 — The Funnel plot assessed the potential publication bias of pooled effect in 28-day mortality for corticosteroids vs. placebo treatment in patients with sepsis. [file DataSheet_1.zip › Data Sheet 2/All supplemental figures/Supplemental Figure 36.tif]

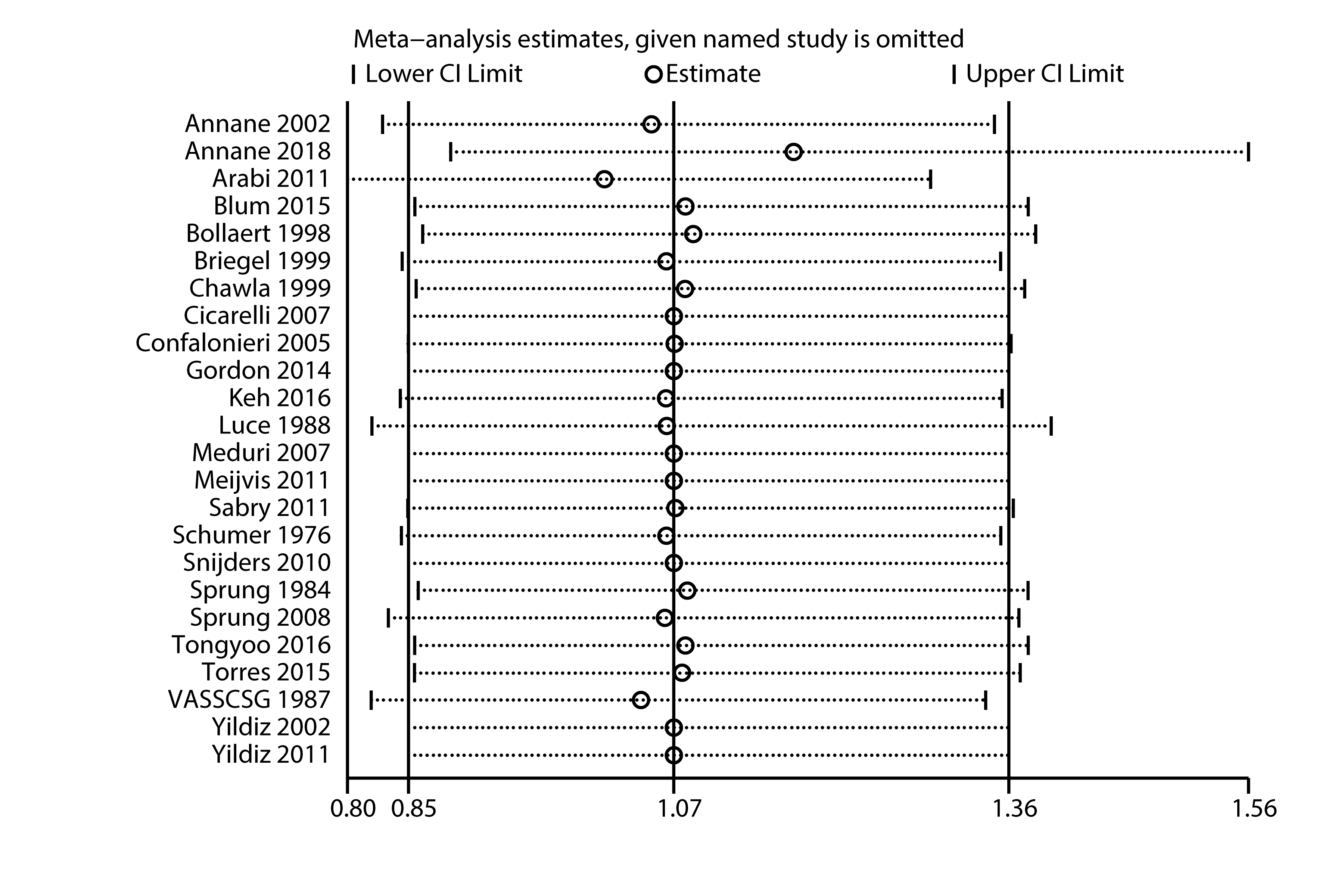

Supplement: Supplementary Figure 1 — The Funnel plot assessed the potential publication bias of pooled effect in 28-day mortality for corticosteroids vs. placebo treatment in patients with sepsis. [file DataSheet_1.zip › Data Sheet 2/All supplemental figures/Supplemental Figure 37.tif]

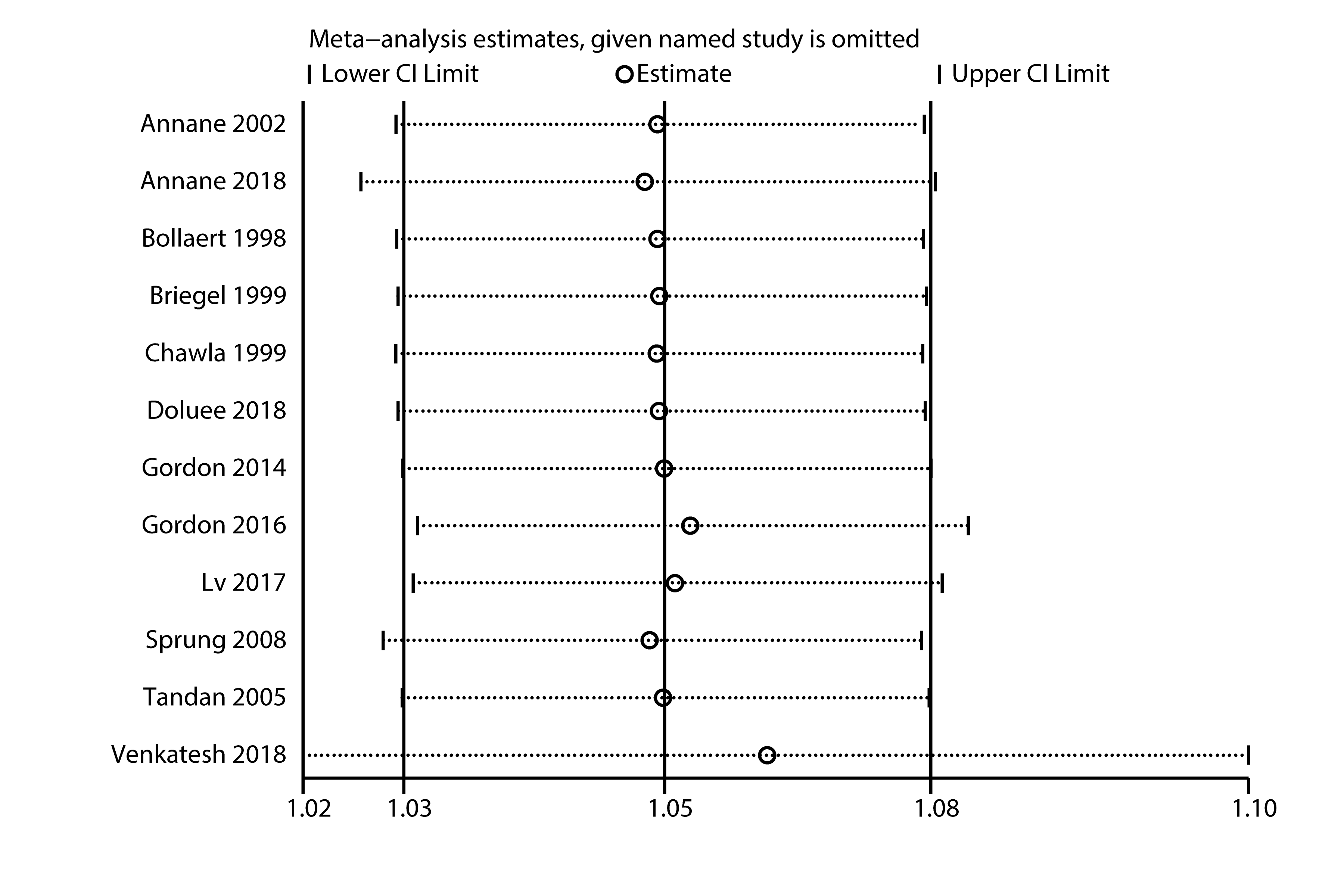

Supplement: Supplementary Figure 1 — The Funnel plot assessed the potential publication bias of pooled effect in 28-day mortality for corticosteroids vs. placebo treatment in patients with sepsis. [file DataSheet_1.zip › Data Sheet 2/All supplemental figures/Supplemental Figure 38.tif]

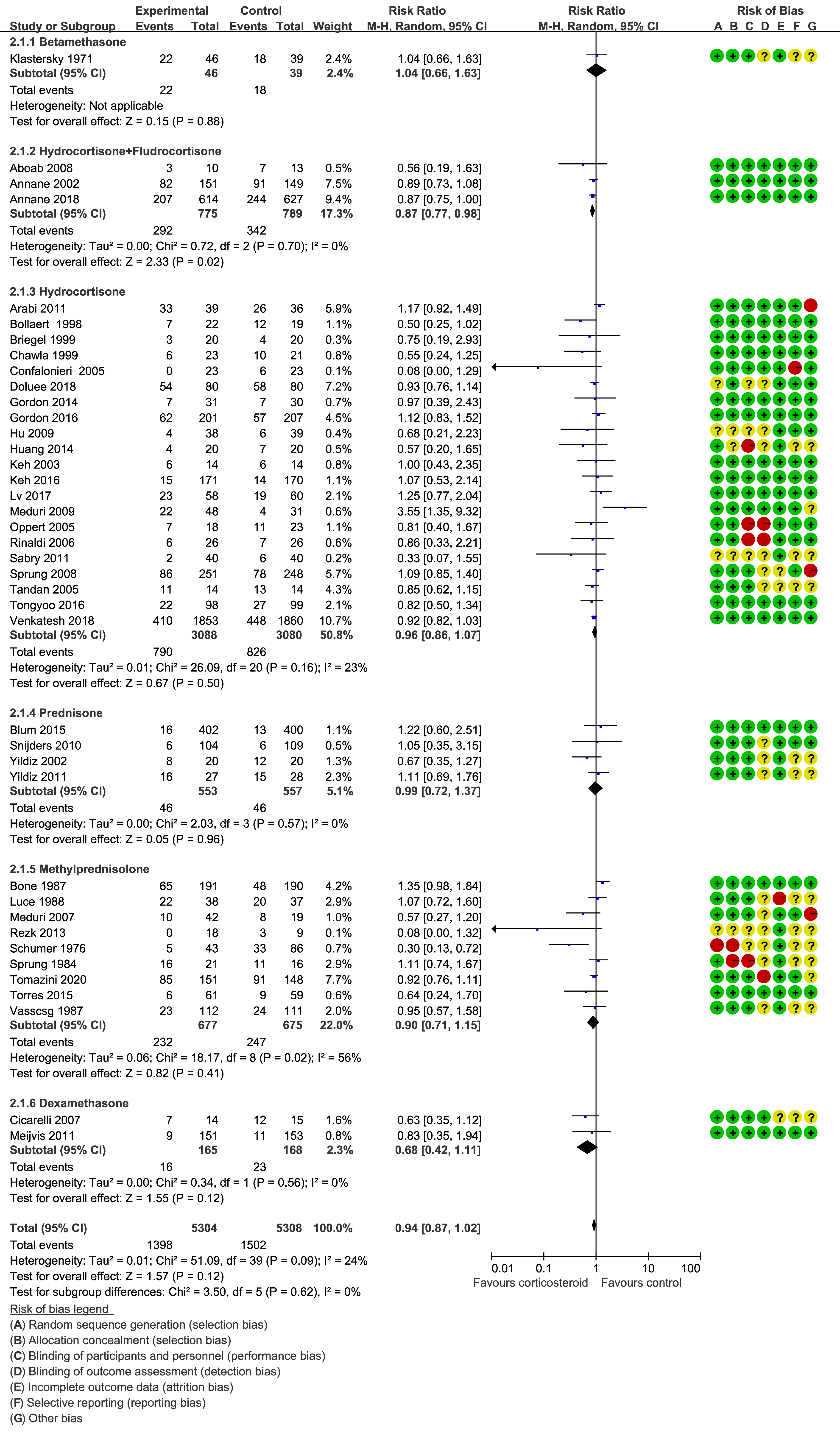

Supplement: Supplementary Figure 1 — The Funnel plot assessed the potential publication bias of pooled effect in 28-day mortality for corticosteroids vs. placebo treatment in patients with sepsis. [file DataSheet_1.zip › Data Sheet 2/All supplemental figures/Supplemental Figure 39.tif]

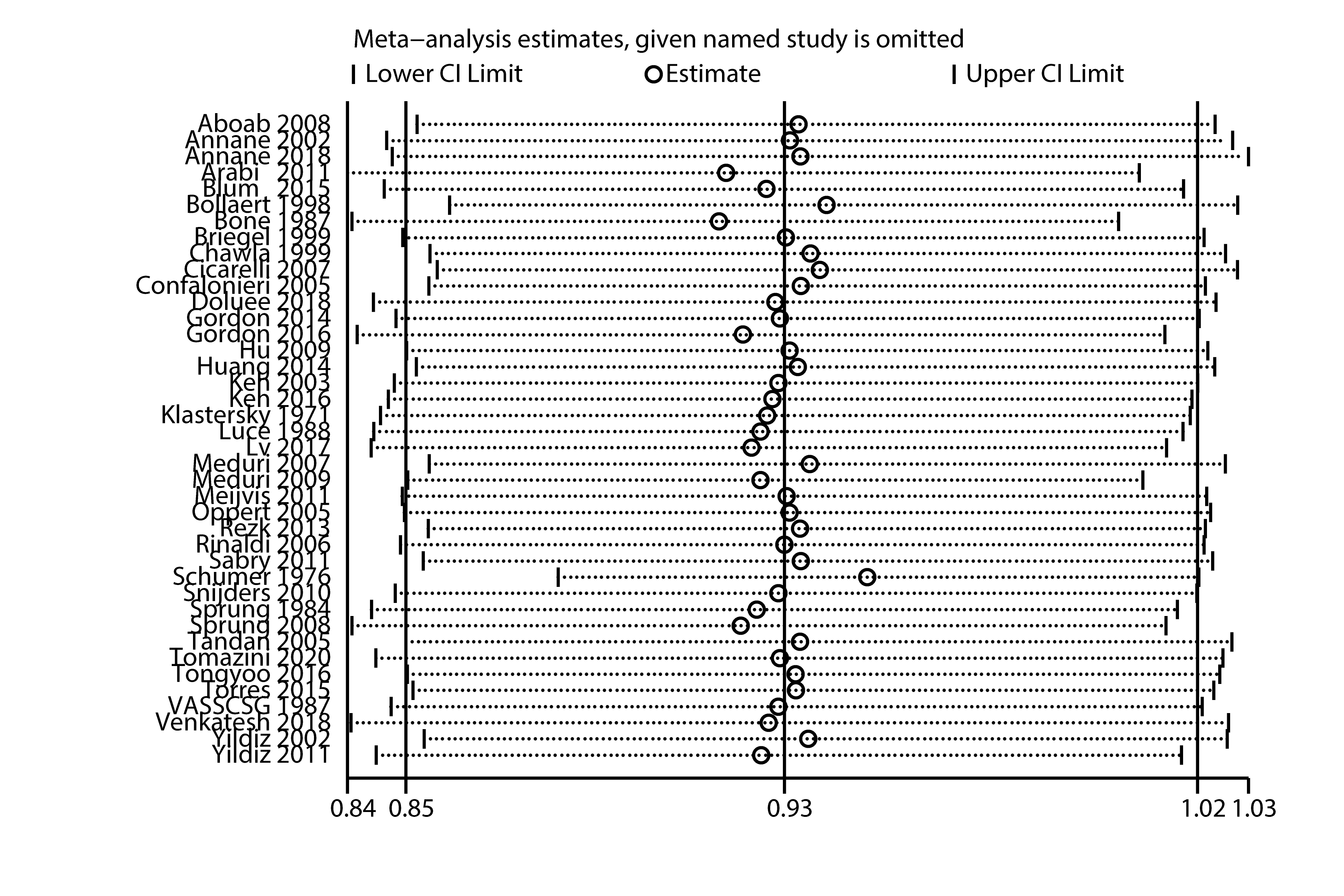

Supplement: Supplementary Figure 1 — The Funnel plot assessed the potential publication bias of pooled effect in 28-day mortality for corticosteroids vs. placebo treatment in patients with sepsis. [file DataSheet_1.zip › Data Sheet 2/All supplemental figures/Supplemental Figure 4.tif]

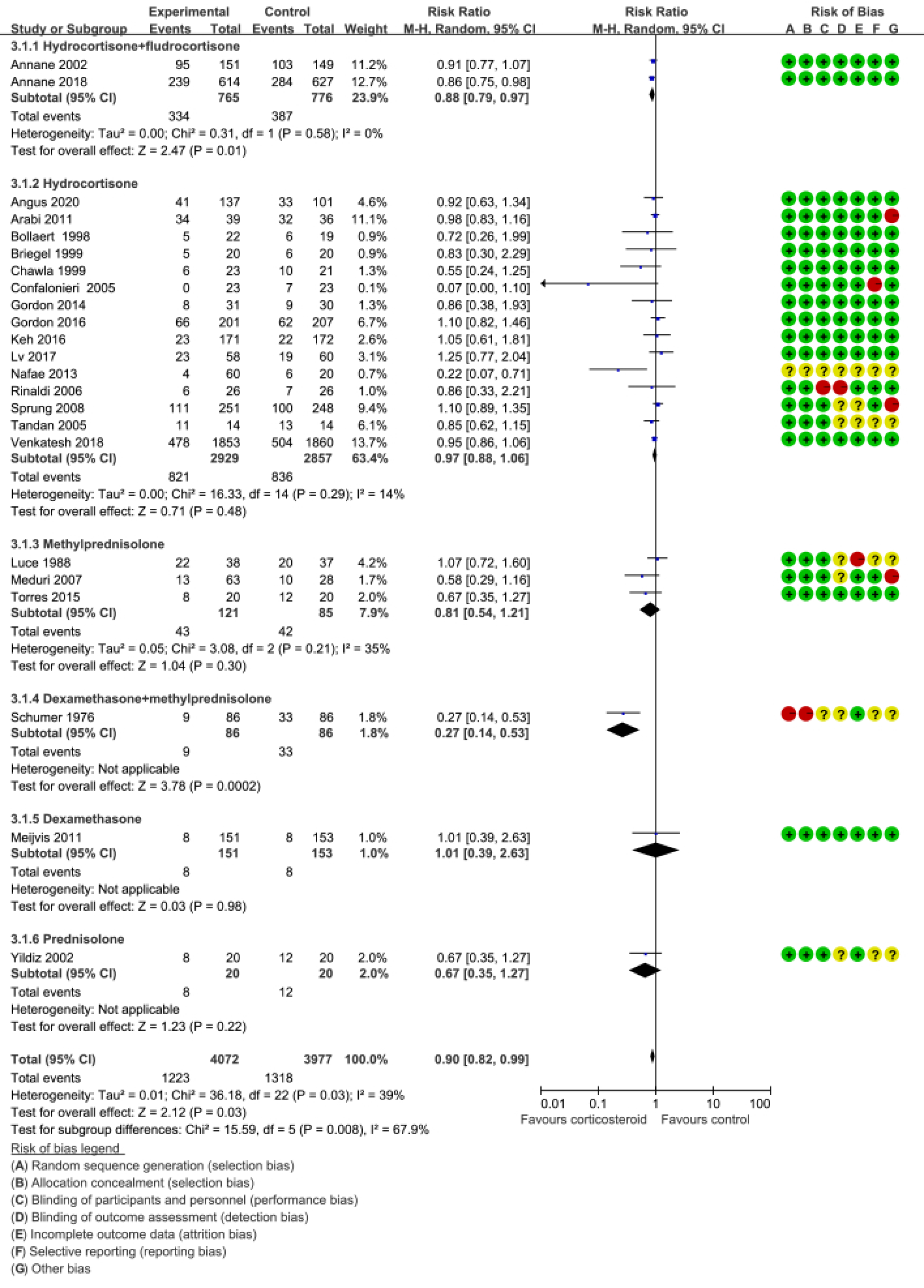

Supplement: Supplementary Figure 1 — The Funnel plot assessed the potential publication bias of pooled effect in 28-day mortality for corticosteroids vs. placebo treatment in patients with sepsis. [file DataSheet_1.zip › Data Sheet 2/All supplemental figures/Supplemental Figure 40.tif]

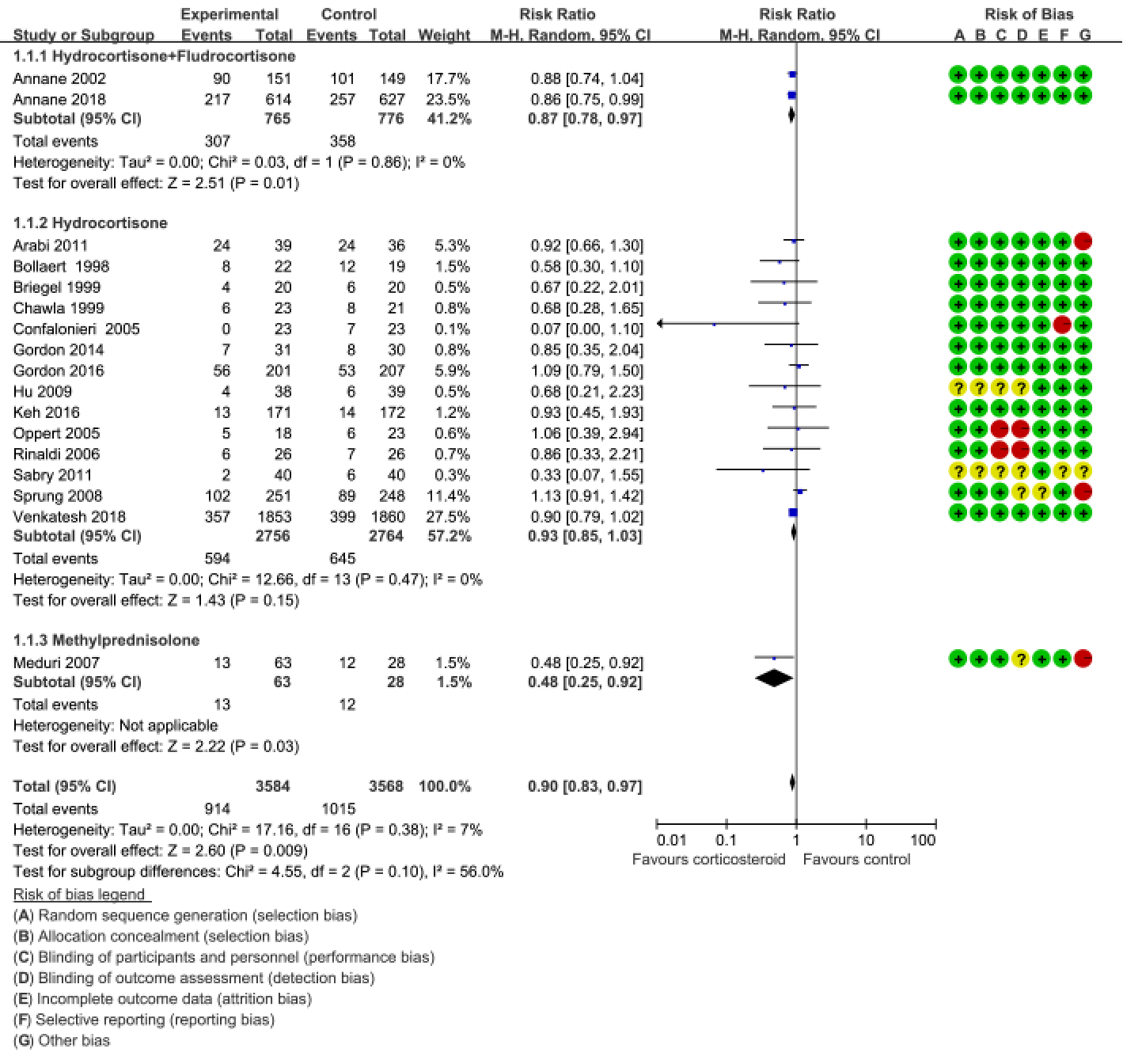

Supplement: Supplementary Figure 1 — The Funnel plot assessed the potential publication bias of pooled effect in 28-day mortality for corticosteroids vs. placebo treatment in patients with sepsis. [file DataSheet_1.zip › Data Sheet 2/All supplemental figures/Supplemental Figure 41.tif]

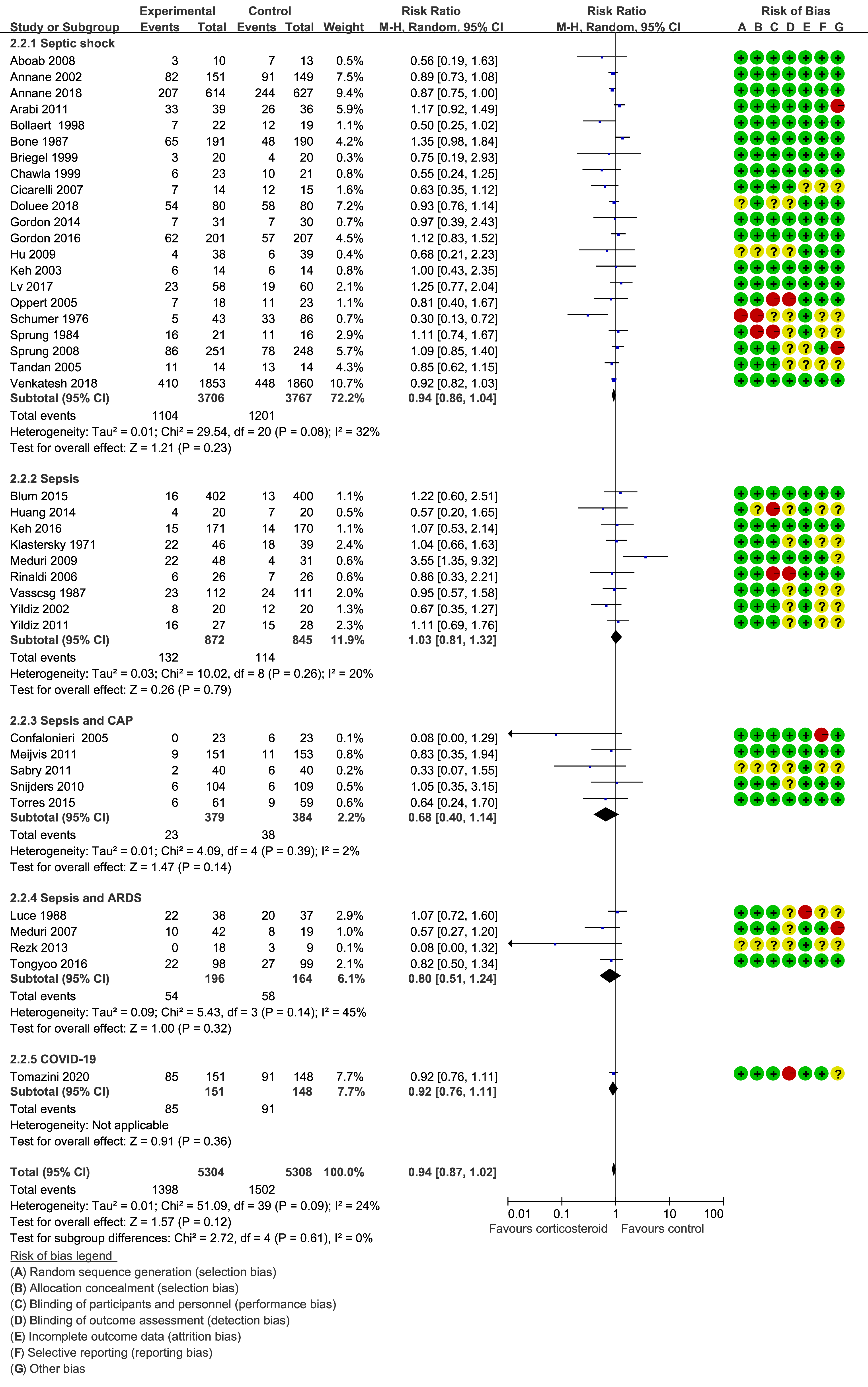

Supplement: Supplementary Figure 1 — The Funnel plot assessed the potential publication bias of pooled effect in 28-day mortality for corticosteroids vs. placebo treatment in patients with sepsis. [file DataSheet_1.zip › Data Sheet 2/All supplemental figures/Supplemental Figure 42.tif]

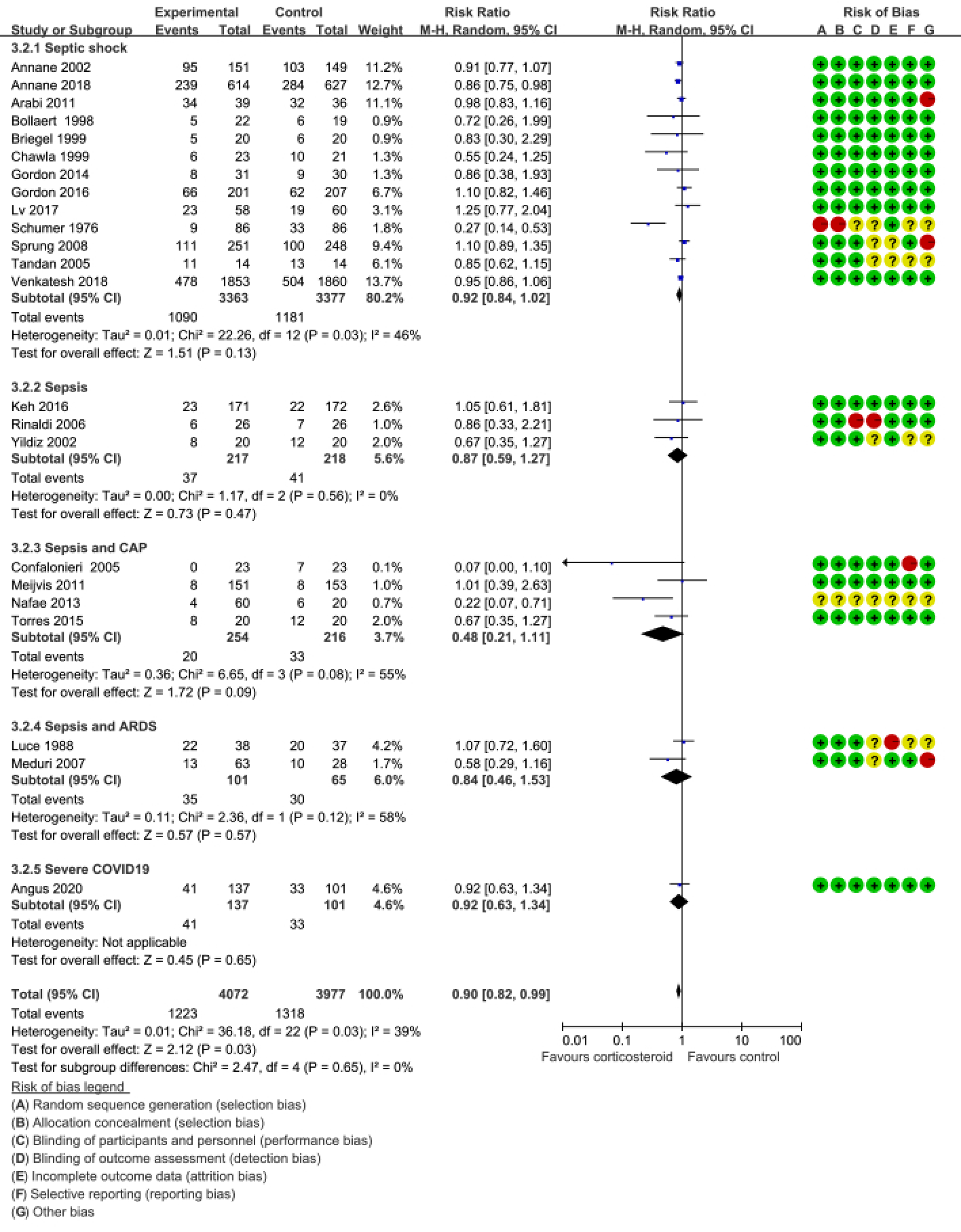

Supplement: Supplementary Figure 1 — The Funnel plot assessed the potential publication bias of pooled effect in 28-day mortality for corticosteroids vs. placebo treatment in patients with sepsis. [file DataSheet_1.zip › Data Sheet 2/All supplemental figures/Supplemental Figure 43.tif]

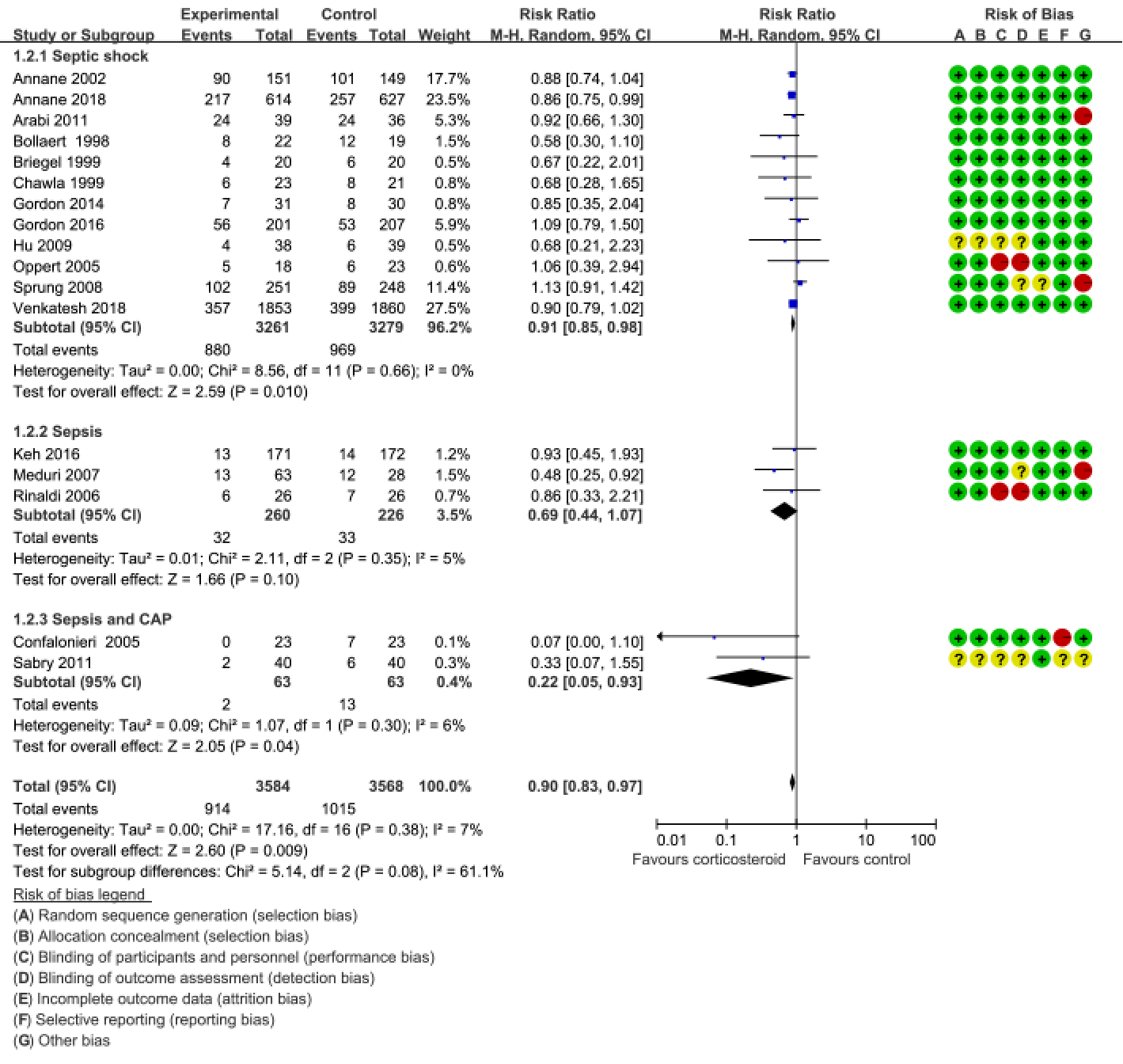

Supplement: Supplementary Figure 1 — The Funnel plot assessed the potential publication bias of pooled effect in 28-day mortality for corticosteroids vs. placebo treatment in patients with sepsis. [file DataSheet_1.zip › Data Sheet 2/All supplemental figures/Supplemental Figure 44.tif]

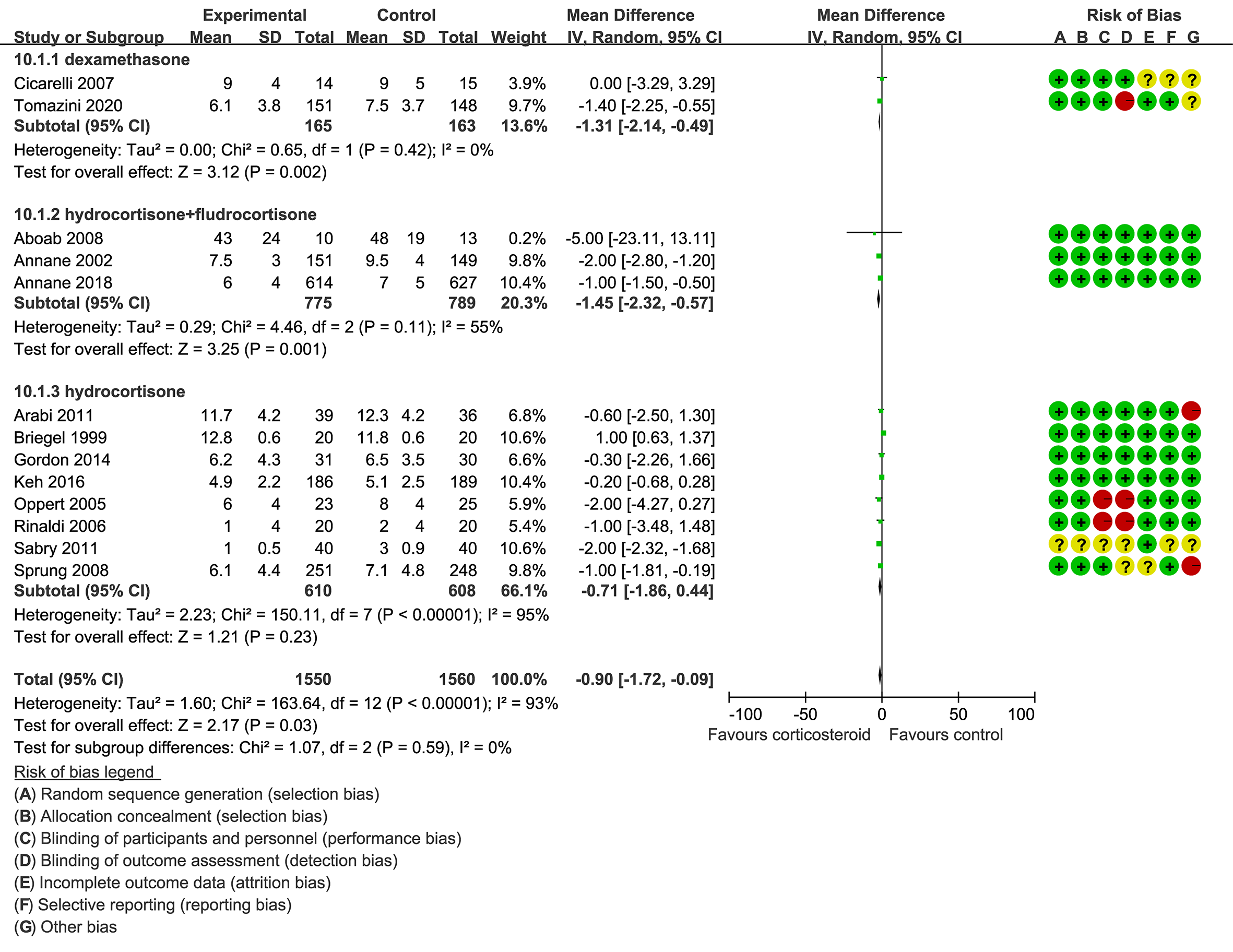

Supplement: Supplementary Figure 1 — The Funnel plot assessed the potential publication bias of pooled effect in 28-day mortality for corticosteroids vs. placebo treatment in patients with sepsis. [file DataSheet_1.zip › Data Sheet 2/All supplemental figures/Supplemental Figure 45.tif]

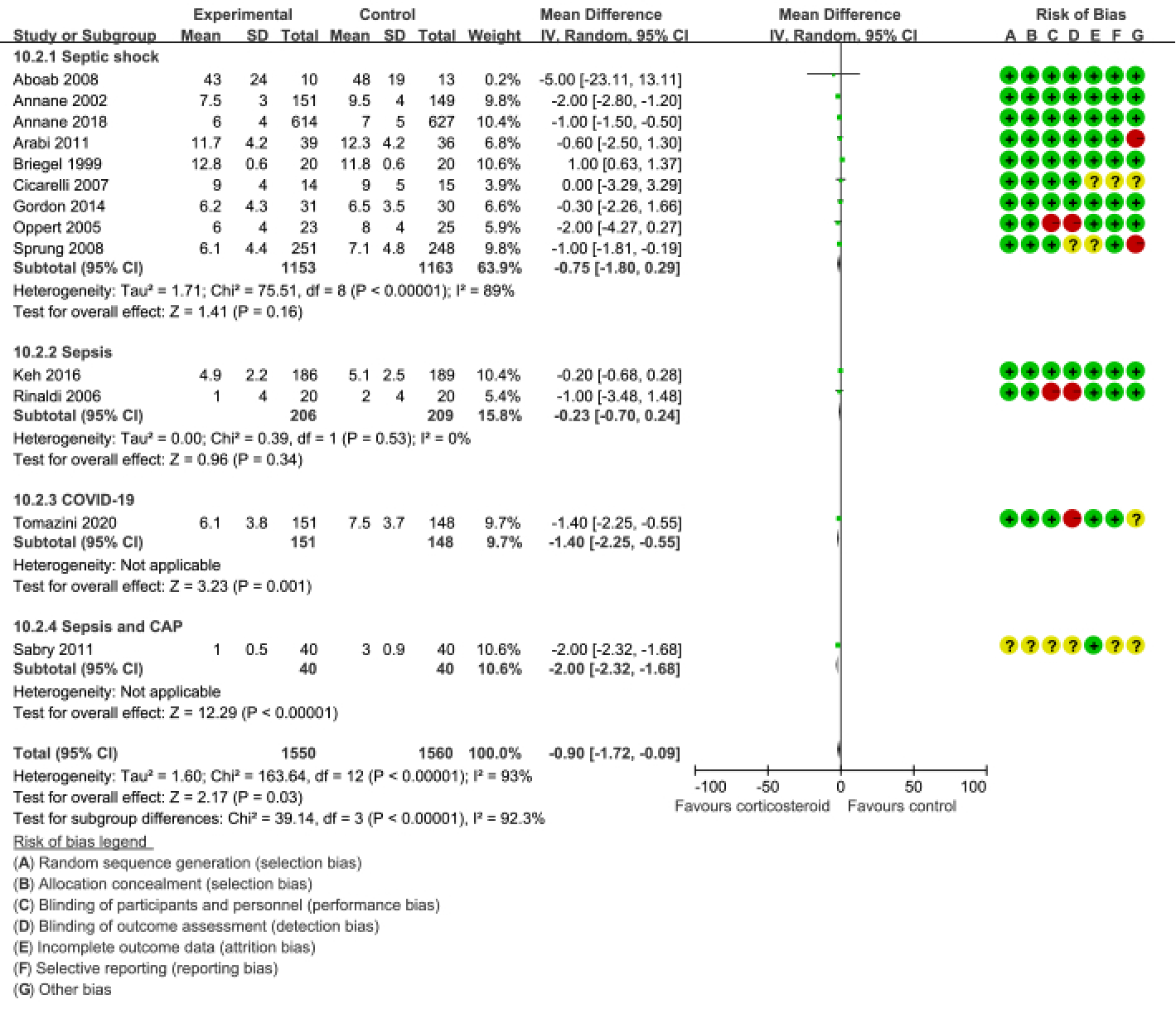

Supplement: Supplementary Figure 1 — The Funnel plot assessed the potential publication bias of pooled effect in 28-day mortality for corticosteroids vs. placebo treatment in patients with sepsis. [file DataSheet_1.zip › Data Sheet 2/All supplemental figures/Supplemental Figure 46.tif]

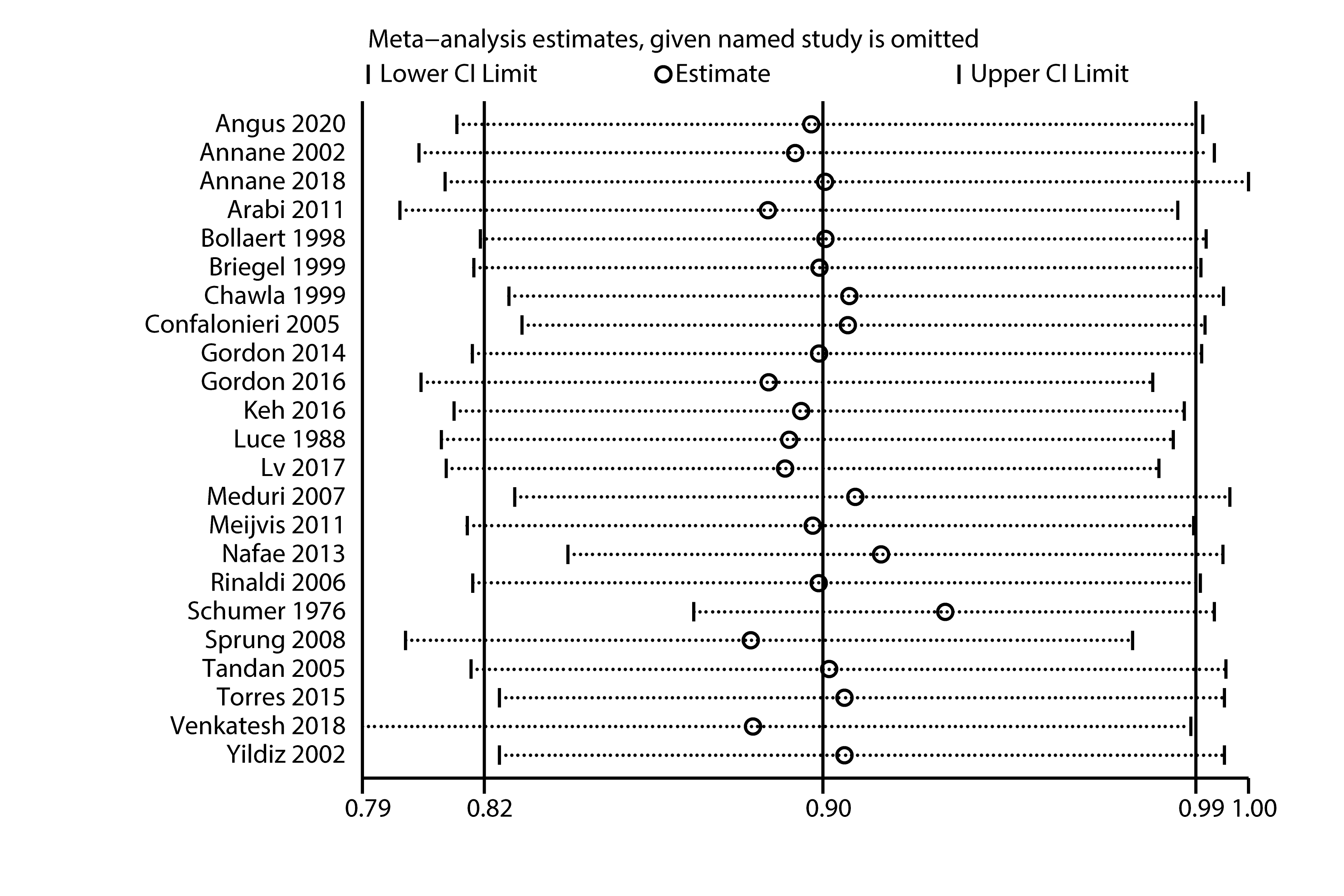

Supplement: Supplementary Figure 1 — The Funnel plot assessed the potential publication bias of pooled effect in 28-day mortality for corticosteroids vs. placebo treatment in patients with sepsis. [file DataSheet_1.zip › Data Sheet 2/All supplemental figures/Supplemental Figure 5.tif]

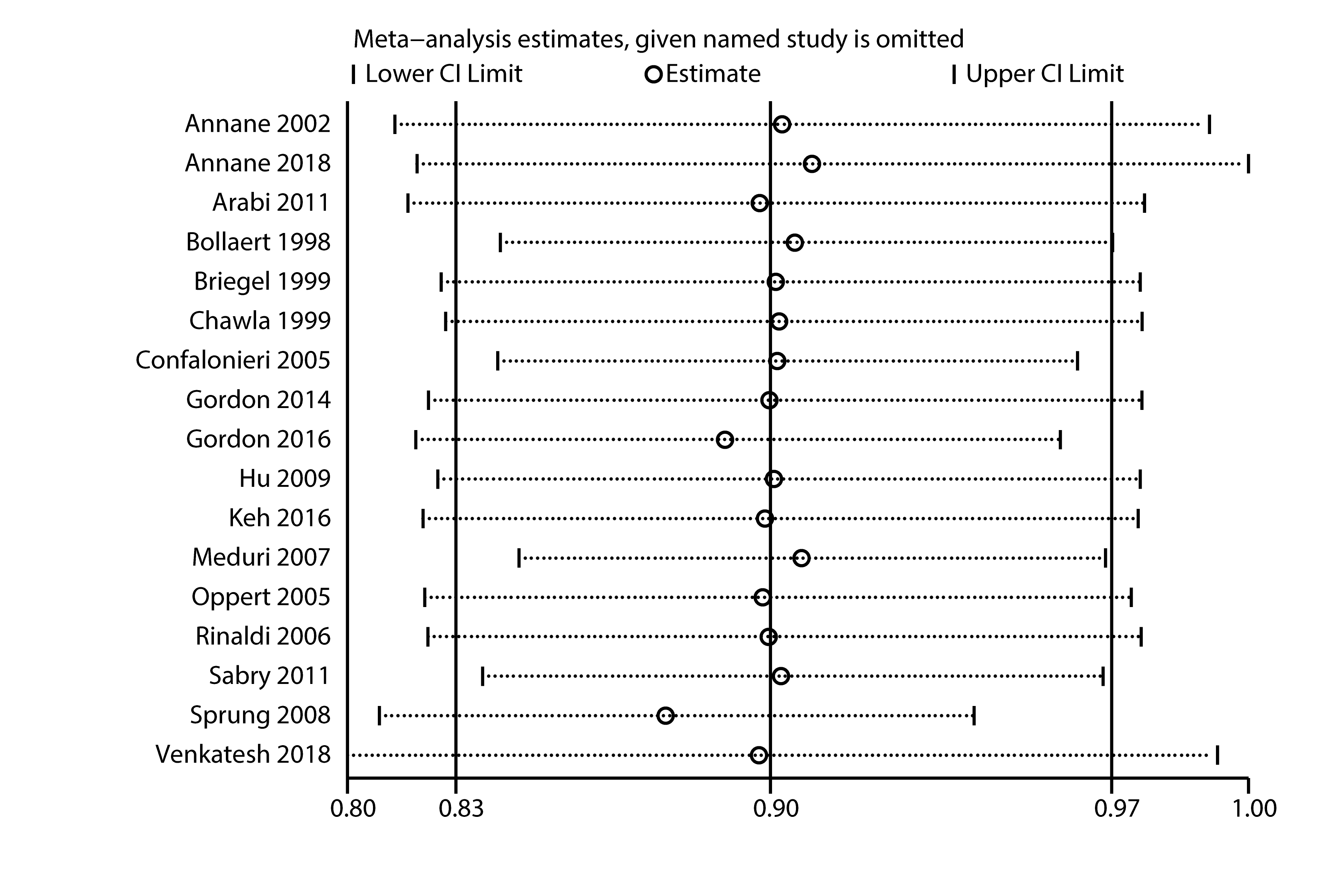

Supplement: Supplementary Figure 1 — The Funnel plot assessed the potential publication bias of pooled effect in 28-day mortality for corticosteroids vs. placebo treatment in patients with sepsis. [file DataSheet_1.zip › Data Sheet 2/All supplemental figures/Supplemental Figure 6.tif]

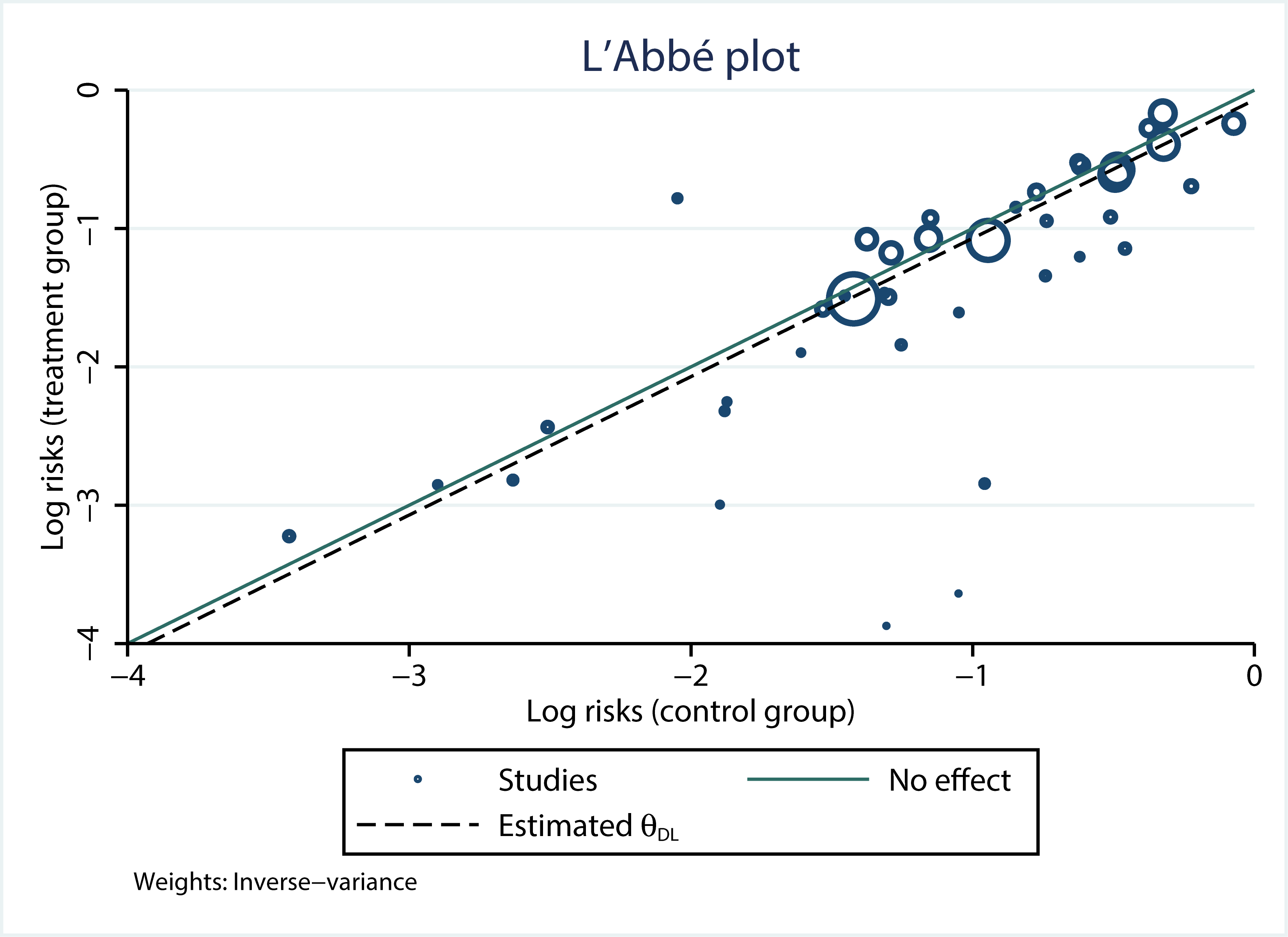

Supplement: Supplementary Figure 1 — The Funnel plot assessed the potential publication bias of pooled effect in 28-day mortality for corticosteroids vs. placebo treatment in patients with sepsis. [file DataSheet_1.zip › Data Sheet 2/All supplemental figures/Supplemental Figure 7.tif]

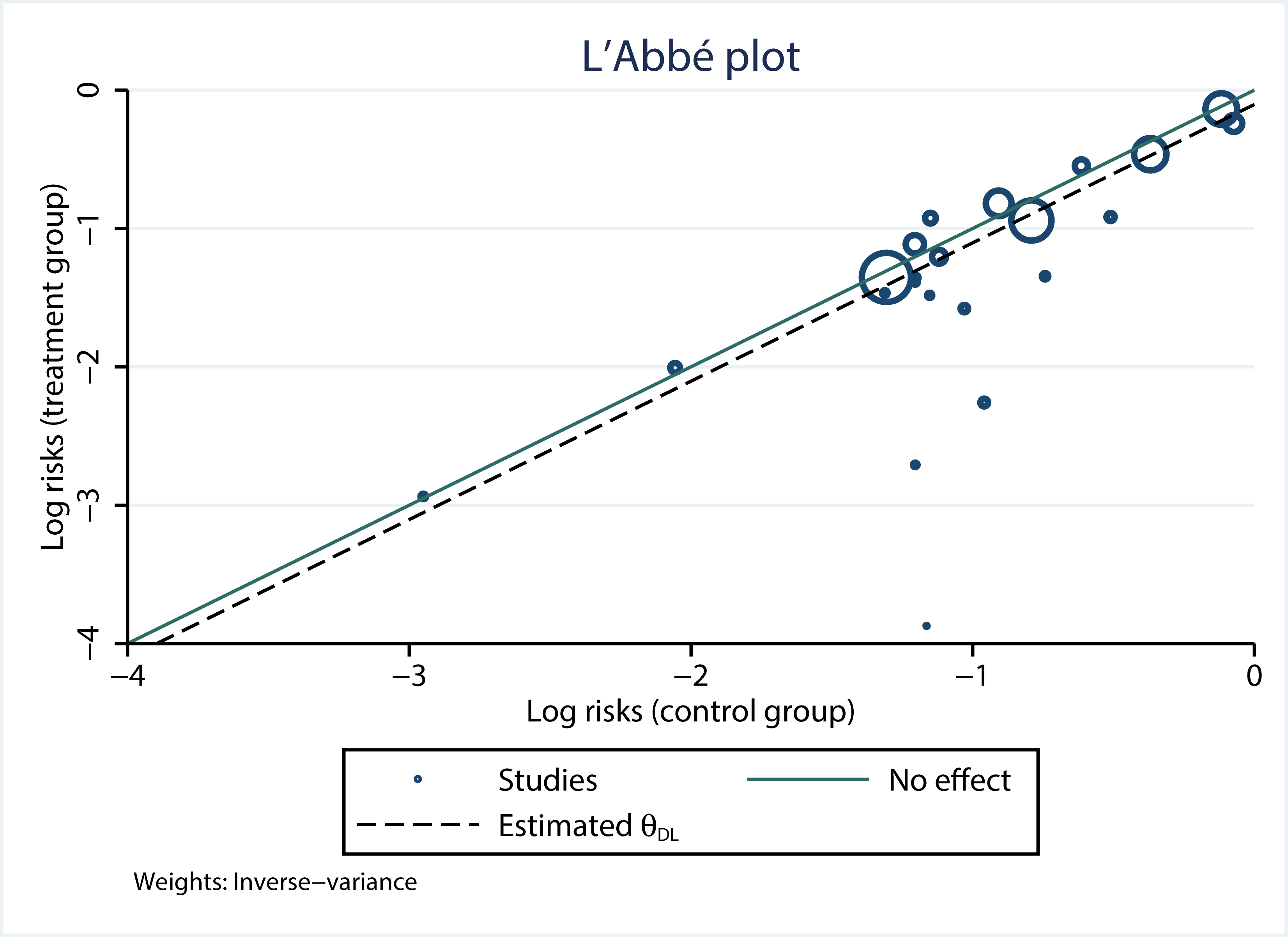

Supplement: Supplementary Figure 1 — The Funnel plot assessed the potential publication bias of pooled effect in 28-day mortality for corticosteroids vs. placebo treatment in patients with sepsis. [file DataSheet_1.zip › Data Sheet 2/All supplemental figures/Supplemental Figure 8.tif]

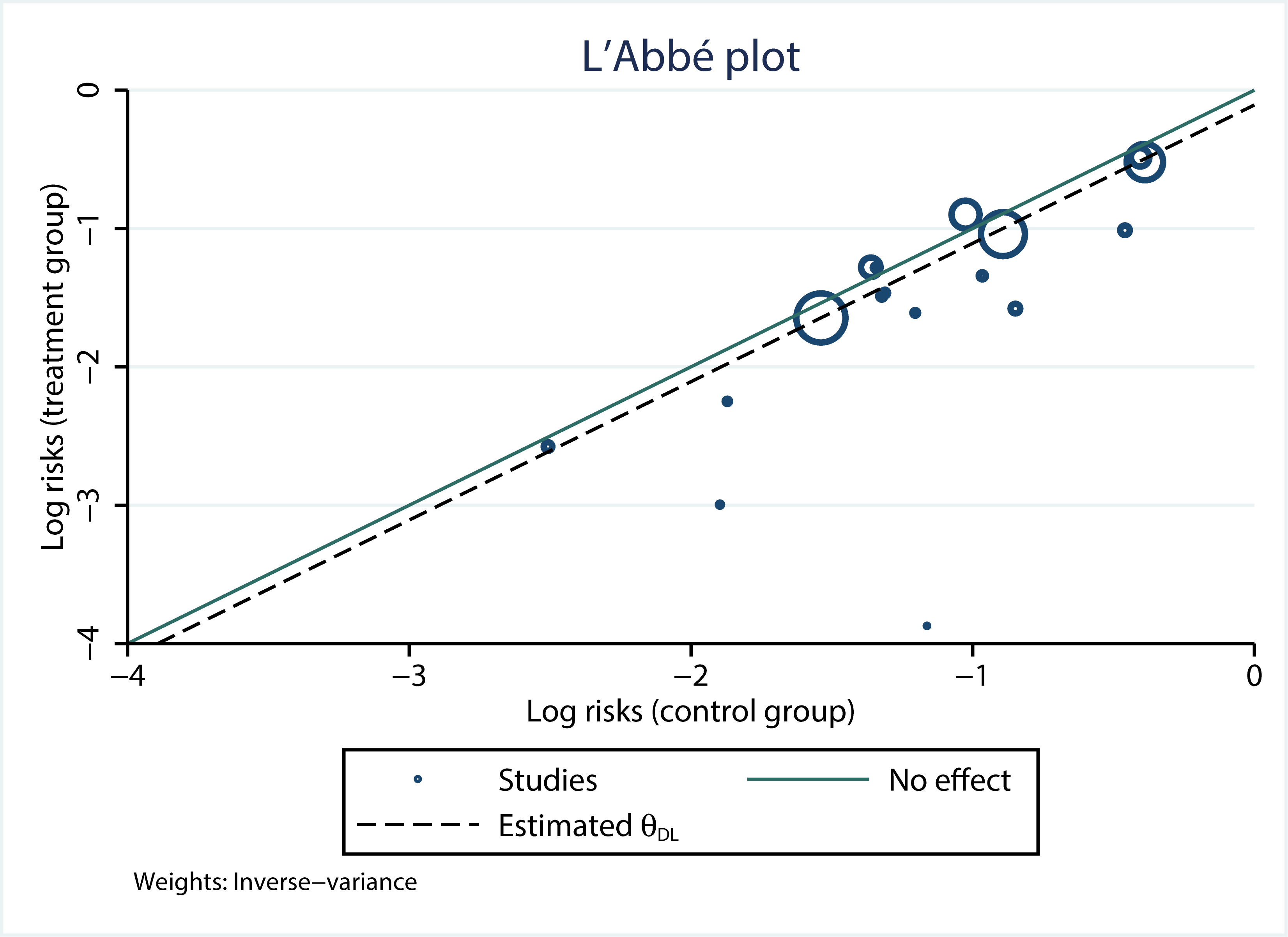

Supplement: Supplementary Figure 1 — The Funnel plot assessed the potential publication bias of pooled effect in 28-day mortality for corticosteroids vs. placebo treatment in patients with sepsis. [file DataSheet_1.zip › Data Sheet 2/All supplemental figures/Supplemental Figure 9.tif]
