# Supplementary material for: Corticosteroids for Treating Sepsis in Adult Patients: A Systematic Review and Meta-Analysis
Source: Front Immunol. 2021 Aug 16;12:709155. doi: 10.3389/fimmu.2021.709155 (PMC8415513; doi:10.3389/fimmu.2021.709155)
Supplement: Supplementary Figure 1 — The Funnel plot assessed the potential publication bias of pooled effect in 28-day mortality for corticosteroids vs. placebo treatment in patients with sepsis. [file DataSheet_1.zip › Data Sheet 2/Supplemental Table 3.DOC]

**Supplemental Table 3. The subgroup analysis based on the ICU type.**

| **Subgroup** | **Included study, n** | **Patients, n** | **RR (95% CI)** | ***I*^2^ (%)** | ***P* value in Heterogeneity** | ***P* value in statistical** |
| --- | --- | --- | --- | --- | --- | --- |
| **ICU mortality** |  |  |  |  |  |  |
| Surgical/Medical ICU | 15 | 7036 | 0.89[0.81,0.98] | 18% | 0.25 | 0.02 |
| Surgical ICU | 1 | 75 | 0.92[0.66,1.30] | NA | NA | 0.64 |
| Medical ICU | 1 | 41 | 1.06[0.39,2.94] | NA | NA | 0.90 |
| **28-day mortality** |  |  |  |  |  |  |
| Surgical/Medical ICU | 33 | 10078 | 0.95[0.88,1.02] | 14 | 0.24 | 0.14 |
| Surgical ICU | 3 | 276 | 0.50[0.13,1.90] | 95 | ＜0.00001 | 0.31 |
| Medical ICU | 4 | 333 | 0.88[0.67,1.16] | 0 | 0.62 | 0.35 |
| **In-hospital mortality** |  |  |  |  |  |  |
| Surgical/Medical ICU | 20 | 7762 | 0.93[0.86,1.00] | 13% | 0.29 | 0.06 |
| Surgical ICU | 2 | 247 | 0.53[0.07,3.89] | 97% | ＜0.00001 | 0.53 |
| Medical ICU | 1 | 40 | 0.67[0.35,1.27] | NA | NA | 0.22 |

Abbreviations: ICU, Intensive Care Unit; NA, Not acquired; RR, Risk ratio.
