# Supplementary figures and images for: Corrigendum: Corticosteroids for Treating Sepsis in Adult Patients: A Systematic Review and Meta-Analysis
Source: Front Immunol. 2021 Nov 5;12:771779. doi: 10.3389/fimmu.2021.771779 (PMC8603401; doi:10.3389/fimmu.2021.771779)

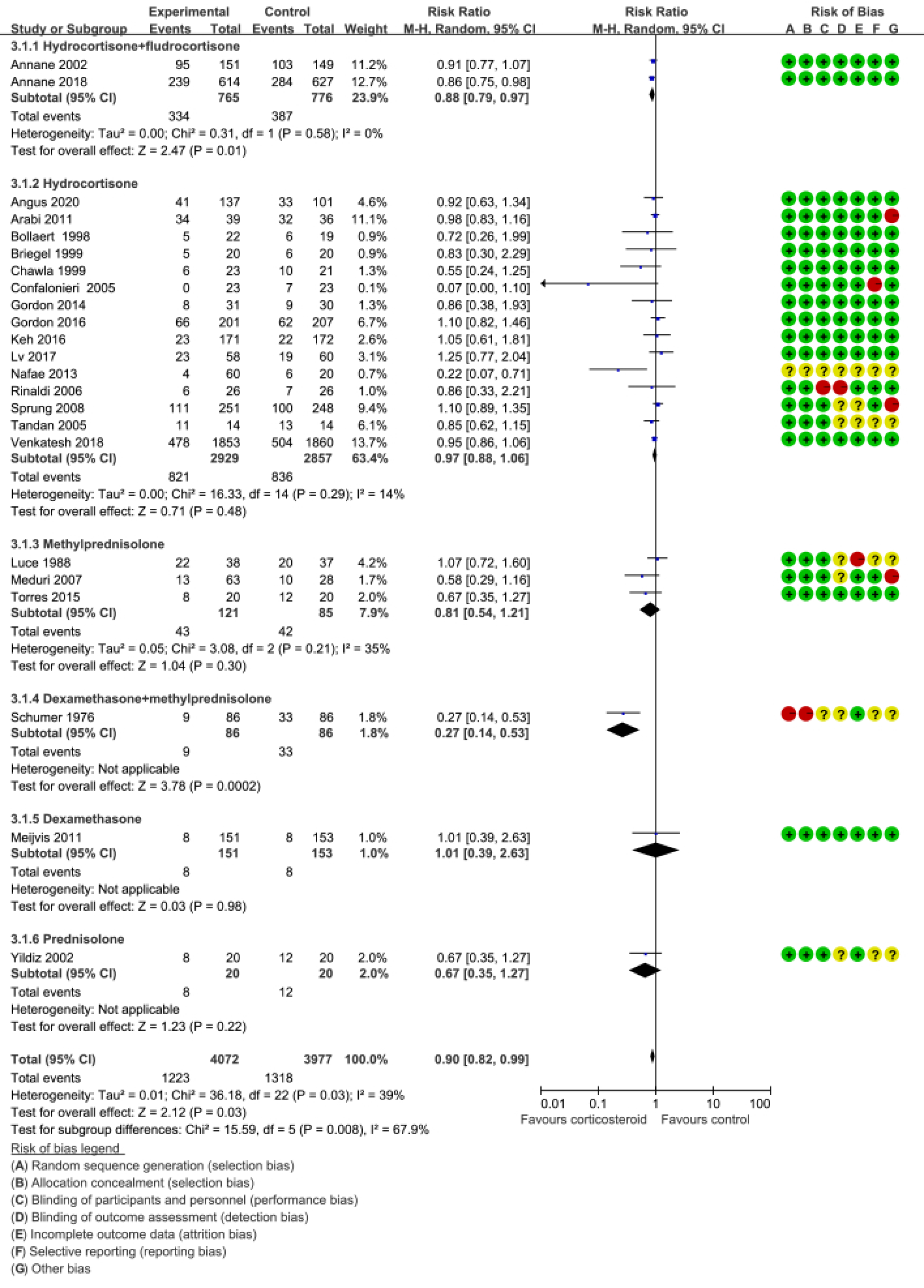

Supplement: Supplementary file 1 [file DataSheet_1.zip › The correct supllemental figures/The correct figures/Supplemental Figure 40.tif]

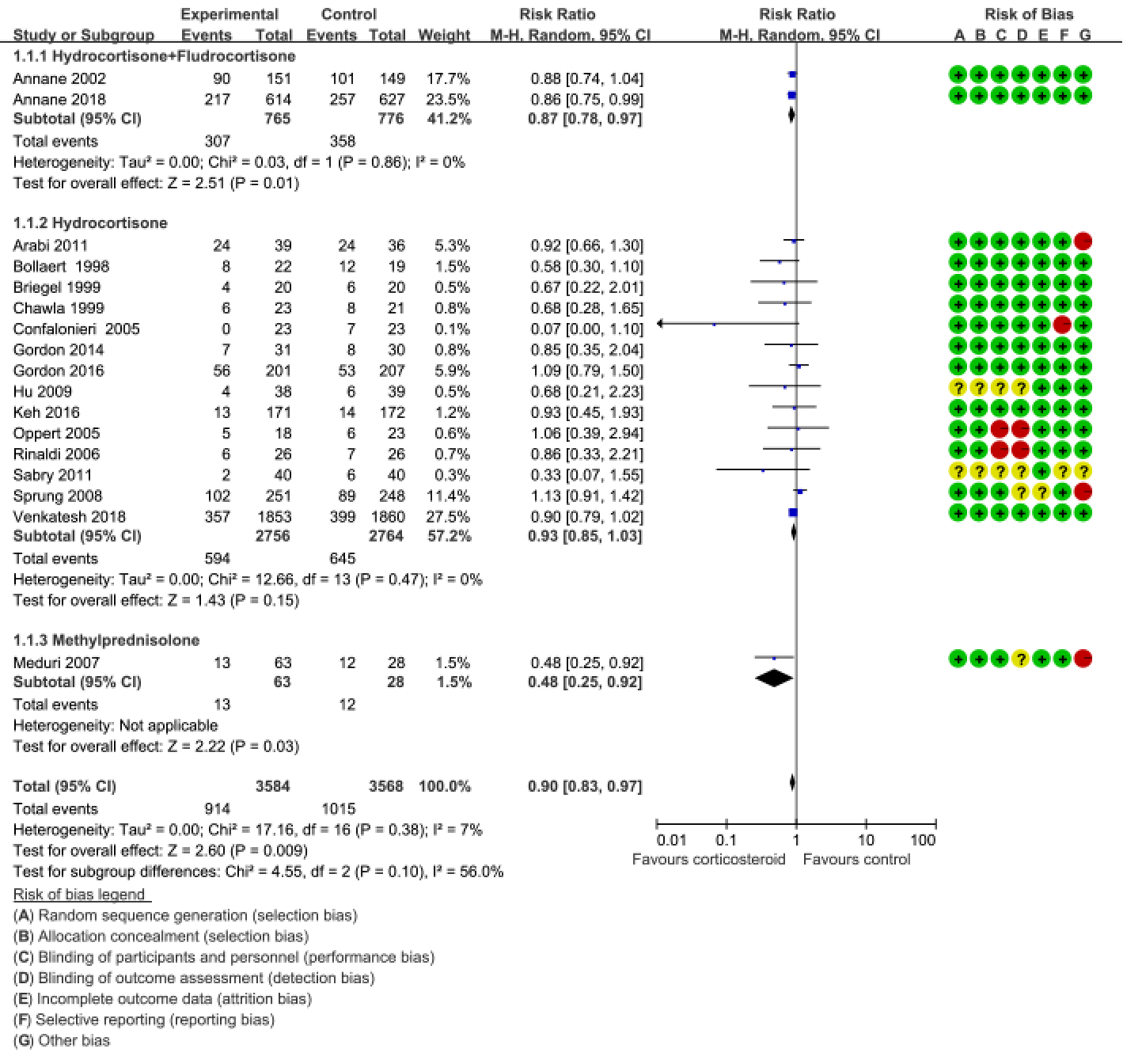

Supplement: Supplementary file 1 [file DataSheet_1.zip › The correct supllemental figures/The correct figures/Supplemental Figure 41.tif]

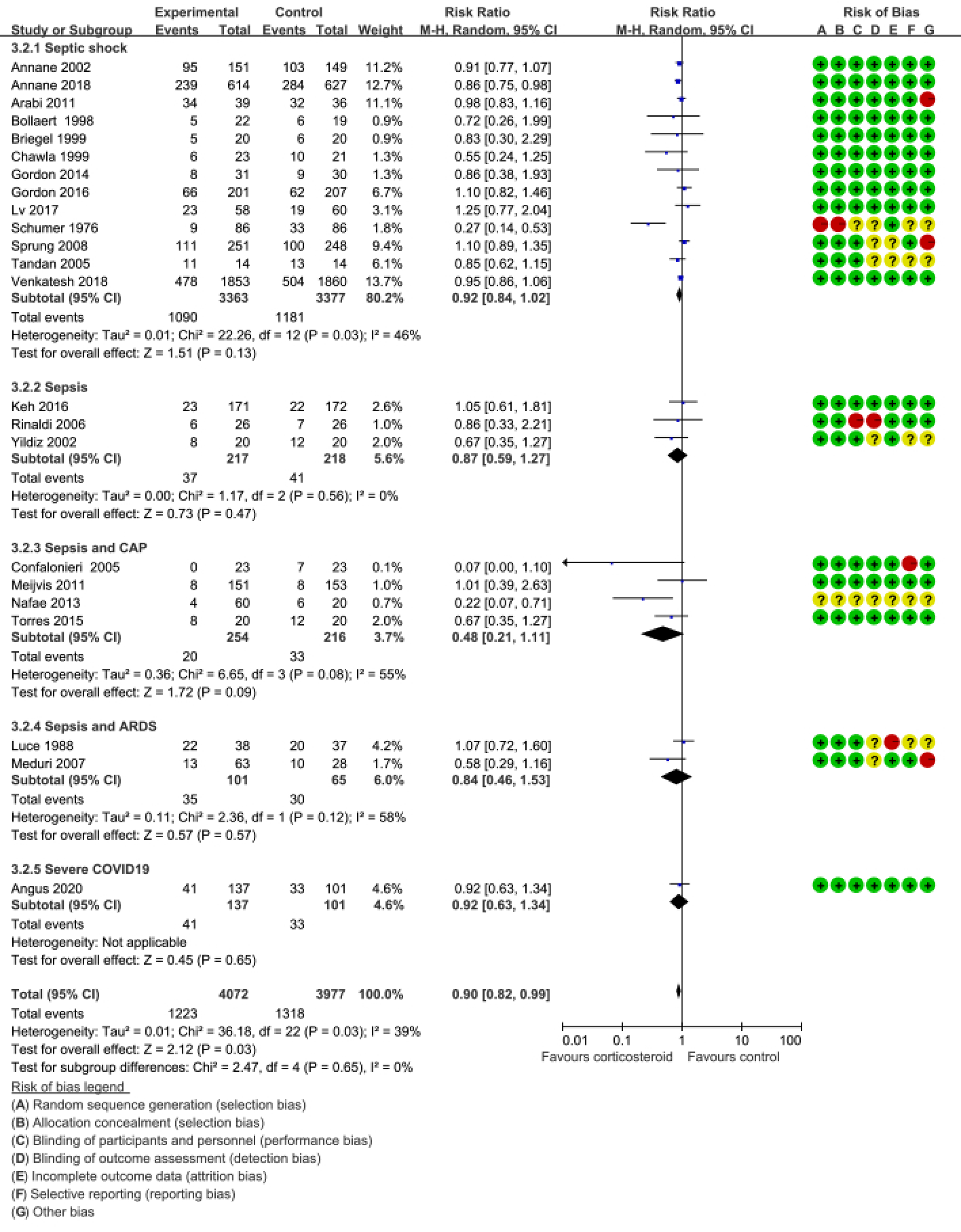

Supplement: Supplementary file 1 [file DataSheet_1.zip › The correct supllemental figures/The correct figures/Supplemental Figure 43.tif]

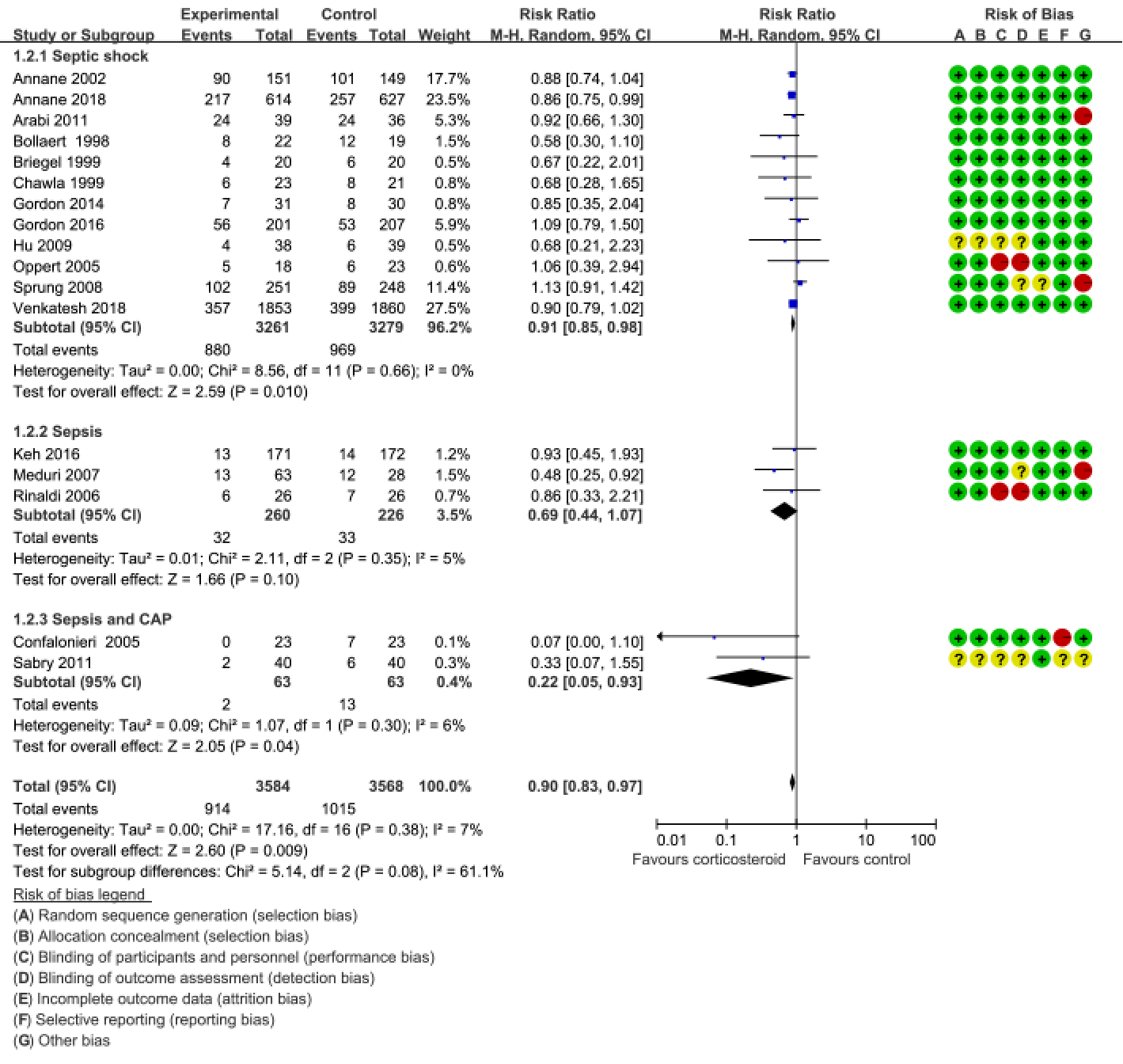

Supplement: Supplementary file 1 [file DataSheet_1.zip › The correct supllemental figures/The correct figures/Supplemental Figure 44.tif]

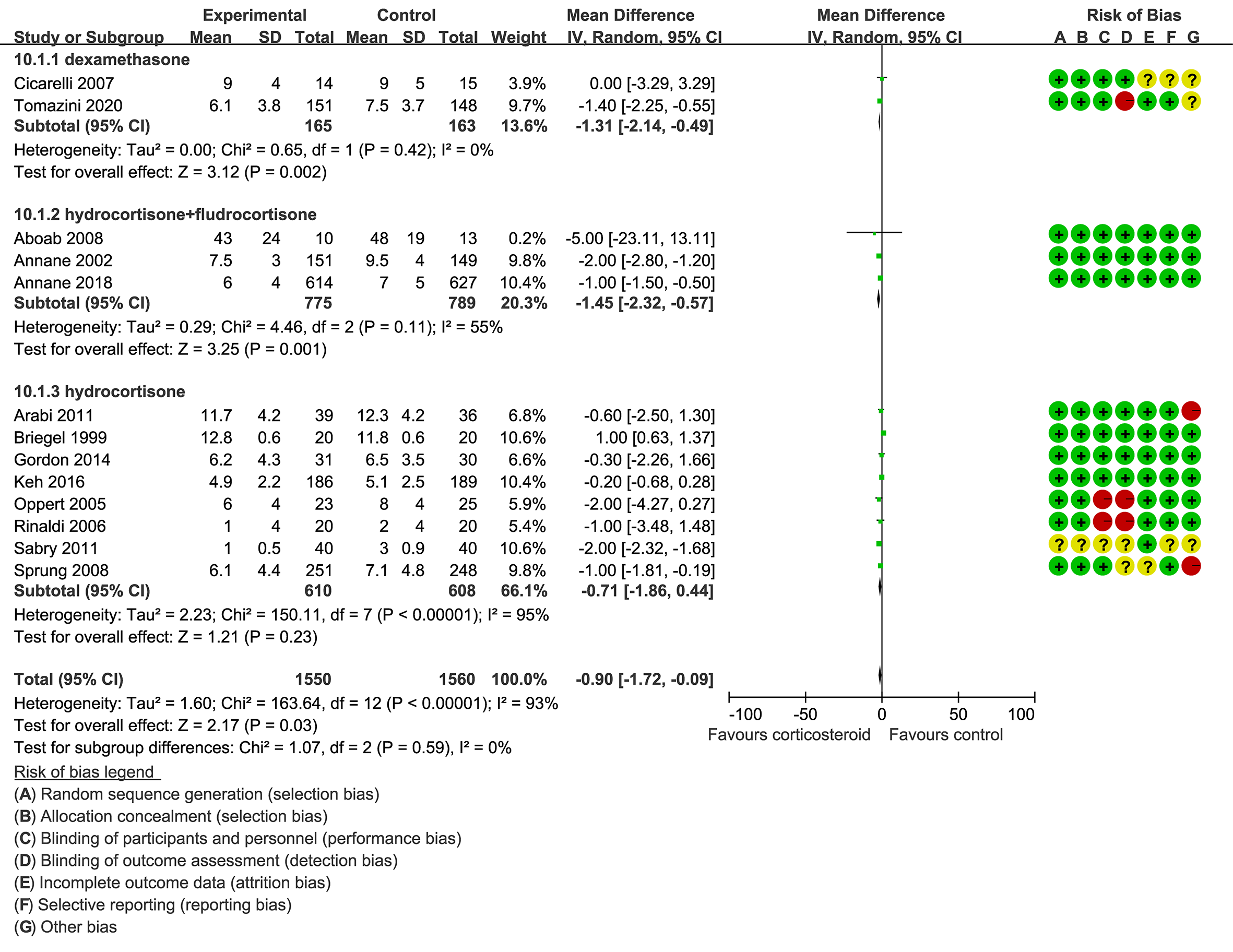

Supplement: Supplementary file 1 [file DataSheet_1.zip › The correct supllemental figures/The correct figures/Supplemental Figure 45.tif]

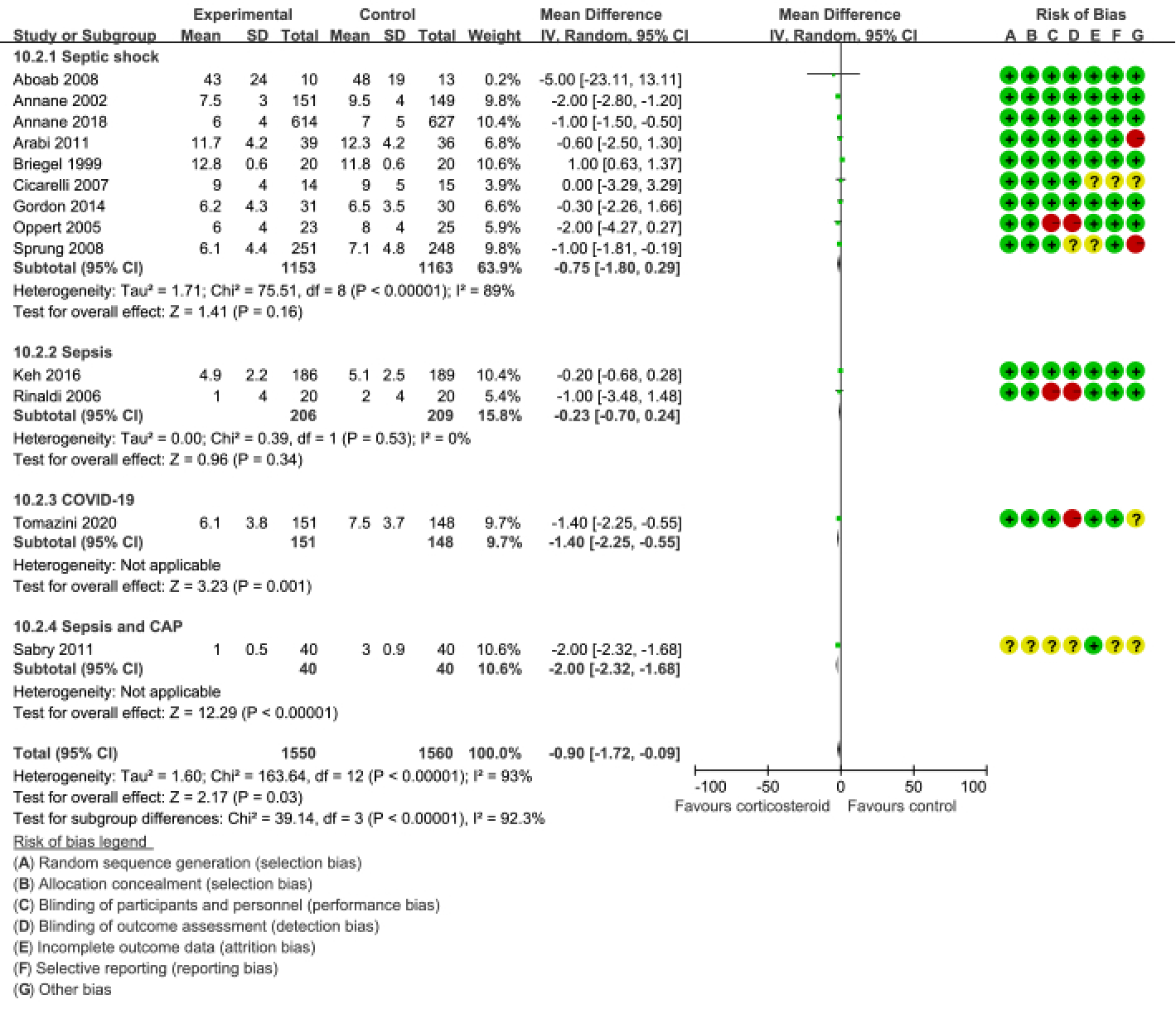

Supplement: Supplementary file 1 [file DataSheet_1.zip › The correct supllemental figures/The correct figures/Supplemental Figure 46.tif]
